# Leveraging multiple data types to estimate the size of the Zika epidemic in the Americas

**DOI:** 10.1371/journal.pntd.0008640

**Published:** 2020-09-28

**Authors:** Sean M. Moore, Rachel J. Oidtman, K. James Soda, Amir S. Siraj, Robert C. Reiner, Christopher M. Barker, T. Alex Perkins

**Affiliations:** 1 Department of Biological Sciences and Eck Institute for Global Health, University of Notre Dame, Notre Dame, Indiana, United States of America; 2 Institute for Health Metrics and Evaluation, Seattle, Washington, United States of America; 3 Department of Pathology, Microbiology, and Immunology, School of Veterinary Medicine, University of California, Davis, Davis, California, United States of America; Australian Red Cross Lifelood, AUSTRALIA

## Abstract

Several hundred thousand Zika cases have been reported across the Americas since 2015. Incidence of infection was likely much higher, however, due to a high frequency of asymptomatic infection and other challenges that surveillance systems faced. Using a hierarchical Bayesian model with empirically-informed priors, we leveraged multiple types of Zika case data from 15 countries to estimate subnational reporting probabilities and infection attack rates (IARs). Zika IAR estimates ranged from 0.084 (95% CrI: 0.067–0.096) in Peru to 0.361 (95% CrI: 0.214–0.514) in Ecuador, with significant subnational variability in every country. Totaling infection estimates across these and 33 other countries and territories, our results suggest that 132.3 million (95% CrI: 111.3-170.2 million) people in the Americas had been infected by the end of 2018. These estimates represent the most extensive attempt to determine the size of the Zika epidemic in the Americas, offering a baseline for assessing the risk of future Zika epidemics in this region.

## Author summary

During the recent Zika epidemic in the Americas millions of people were likely infected, but the true size of the epidemic is unknown because of gaps in the surveillance system. The infection attack rate (IAR)—defined as the proportion of the population that was infected over the course of the epidemic—has important implications for the longer-term epidemiology of Zika in the region, such as the timing, location, and likelihood of future outbreaks. To estimate the IAR and the total number of people infected, we leveraged multiple types of Zika case data from 15 countries and territories where subnational data were publicly available. Datasets included confirmed and suspected Zika cases in pregnant women and in the total population, Zika-associated Guillan-Barré syndrome cases, and cases of congenital Zika syndrome. We used a hierarchical Bayesian model with empirically-informed priors that leveraged the different case report types to simultaneously estimate national and subnational reporting probabilities, the fraction of symptomatic infections, and subnational IARs. In these 15 countries and territories, estimates of Zika IAR ranged from 0.084 (95% CrI: 0.067–0.096) in Peru to 0.361 (95% CrI: 0.214–0.514) in Ecuador. Totaling these infection estimates across these and 33 other countries and territories in the region, our results suggest that 132.3 million (95% CrI: 111.3-170.2 million) people in the Americas were infected with ZIKV by the end of 2018.

## Introduction

Zika virus is a mosquito-borne pathogen that was first identified in Uganda in 1947 [1]. Smaller Zika outbreaks have occurred in Africa, Asia, and the Pacific islands since its discovery, but there had been no confirmed cases in the Americas (excluding Easter Island, Chile) prior to the first confirmation of a Zika case in Brazil in May, 2015 [2]. Subsequent to its discovery in Brazil, the epidemic spread rapidly and cases were reported throughout the Americas over the next two years [3]. The Zika epidemic generated a large amount of concern in the public health community and the general public, leading to a declaration of a Public Health Emergency of International Concern by the World Health Organization (WHO) in February, 2016 [4], because of the discovery of a link between ZIKV infection in pregnant women and congenital Zika syndrome (CZS) in newborns [5–8]. ZIKV infection is also associated with rare but serious neurological disorders, particularly Guillan-Barré syndrome [9]. Following the large epidemic from 2015-2017, substantially fewer Zika cases have been reported in 2018 and 2019 [3].

Now that the initial wave of the ZIKV epidemic in the Americas has passed, there are a number of unanswered questions about what will happen next. If the remaining population at risk is large, then additional outbreaks in the coming years are still possible. On the other hand, modeling has suggested that if a large proportion of the population is now immune, herd immunity will likely prevent another large epidemic for more than a decade assuming life-long immunity following recovery [10]. This scenario could lead to a slow buildup of new susceptible individuals over time, and of particular concern, an eventual buildup of susceptible women of childbearing age if a ZIKV vaccine is not licensed and broadly deployed [10–12]. The number of recent ZIKV infections could also have relevance for the epidemiology of dengue virus (DENV) in the region [10]. There is evidence of an interaction between ZIKV and DENV via the human immune response to infection with either virus [13–17]. If a ZIKV infection provides any temporary cross-protection to DENV, then the reduction in dengue incidence in several Latin American countries over the past few years [18] could be followed by a large dengue epidemic as this temporary cross-protection wanes.

The Pan American Health Organization (PAHO) reported suspected and confirmed Zika cases for every country and territory in the Americas, but these reported cases vastly underestimate the total number of ZIKV infections due to inadequate surveillance, the non-specificity of ZIKV symptoms, and the high proportion of asymptomatic infections [11, 19, 20]. Underreporting is particularly an issue for pathogens such as ZIKV where the majority of infections are asymptomatic or produce only mild symptoms [21, 22]. Estimates of ZIKV infections from blood donors in Puerto Rico through 2016 suggest that almost 470,000 people might have been infected in Puerto Rico alone [23]. High ZIKV seroprevalence estimates from several major cities with populations of more than one million—46% in Managua, Nicaragua [24], 63-68% in Salvador, Brazil [25, 26], and 64% in Recife, Brazil [27]—also suggest that the 806,928 suspected and confirmed cases reported by PAHO represent only a small fraction of the total number of ZIKV infections.

In this study, we take advantage of several features of the recent Zika epidemic that allow us to estimate national and subnational IARs throughout the Americas. First, due to the WHO emergency declaration, surveillance for Zika began in most countries relatively early in the epidemic, and all countries and territories in the Americas reported case data to PAHO. Second, in addition to reporting suspected and confirmed Zika cases in the entire population, many countries also reported Zika cases among pregnant women, CZS or microcephaly cases, and Guillan-Barré syndrome (GBS) cases, providing additional information about the underlying IAR. Third, a number of countries have published subnational Zika surveillance data, which increases the sample sizes for estimation purposes. The subnational data also allows us to capture spatial heterogeneity in ZIKV IAR within these countries. Using a hierarchical Bayesian model fit to multiple data types we estimated the subnational IARs and reporting probabilities for each data type. Estimated IAR and reporting probabilities were then used to extrapolate national-level case data from the rest of the American countries and territories to provide an estimate of the total number of ZIKV infections across this region.

## Methods

### Data

The cumulative numbers of suspected and confirmed Zika cases in each country and territory in the Americas, as well as the number of confirmed CZS cases, were reported by PAHO on a weekly basis through the first week of 2018 [3]. In addition, PAHO also published country reports following epidemiological week 35 in 2017 that contained additional information, including the total number of cases of microcephaly and Guillan-Barré syndrome (GBS) associated with Zika, where available [28]. Because ZIKV infection attack rates could vary significantly within a country, we restricted our primary analysis to countries and territories where we were able to obtain Zika data at a subnational level for at least one data type. In total, we were able to estimate subnational and national ZIKV IARs for 15 countries and territories (Additional details in S1 Appendix and S1 Table). The included countries were Mexico, all of the countries of Central America (Belize, Costa Rica, El Salvador, Guatemala, Honduras, Nicaragua, Panama), Bolivia, Brazil, Colombia, Ecuador, and Peru in South America, and the Domican Republic and Puerto Rico from the Caribbean. These 15 countries and territories have a combined population of 507.1 million out of a total population of 641.9 million in the Americas (excluding the United States and Canada) and therefore represent a signficant fraction of the population at risk.

The data types considered were confirmed Zika cases, suspected Zika cases, microcephaly cases associated with a ZIKV infection in the mother, and Zika-associated cases of GBS. In addition, due to the risk of CZS in newborns, many countries also reported the number of pregnant women with either a suspected or confirmed ZIKV infection, which we treated as seperate data points from the confirmed and suspected cases in the entire population due to the differences in the surveillance and reporting systems for these case types. Suspected Zika cases were defined by WHO/PAHO as a patient with a rash and two or more of the following symptoms: fever, conjunctivitis, arthralgia, myalgia, or peri-articular edema [29]. Reporting of Zika-associated microcephaly and GBS cases varied by country, with some reporting only cases associated with a lab-confirmed ZIKV infection and others both confirmed and suspected cases.

Where available, we obtained Zika data at the first administrative level (e.g., province or state) within a country or territory. Lower level data were aggregated to the first administrative level in cases where they were available. National and subnational population estimates were generated from WorldPop 2015 population rasters for each country or territory [30, 31]. The number of pregnant women in each country potentially at risk of a ZIKV infection was estimated using 2015 pregnancies rasters from WorldPop, and the number of births at risk for microcephaly were estimated using the WorldPop 2015 births rasters [32]. All of the data used in our analysis, along with model code, are located in the Github repository https://github.com/mooresea/Zika_IAR.

### Model

**Fig 1:**
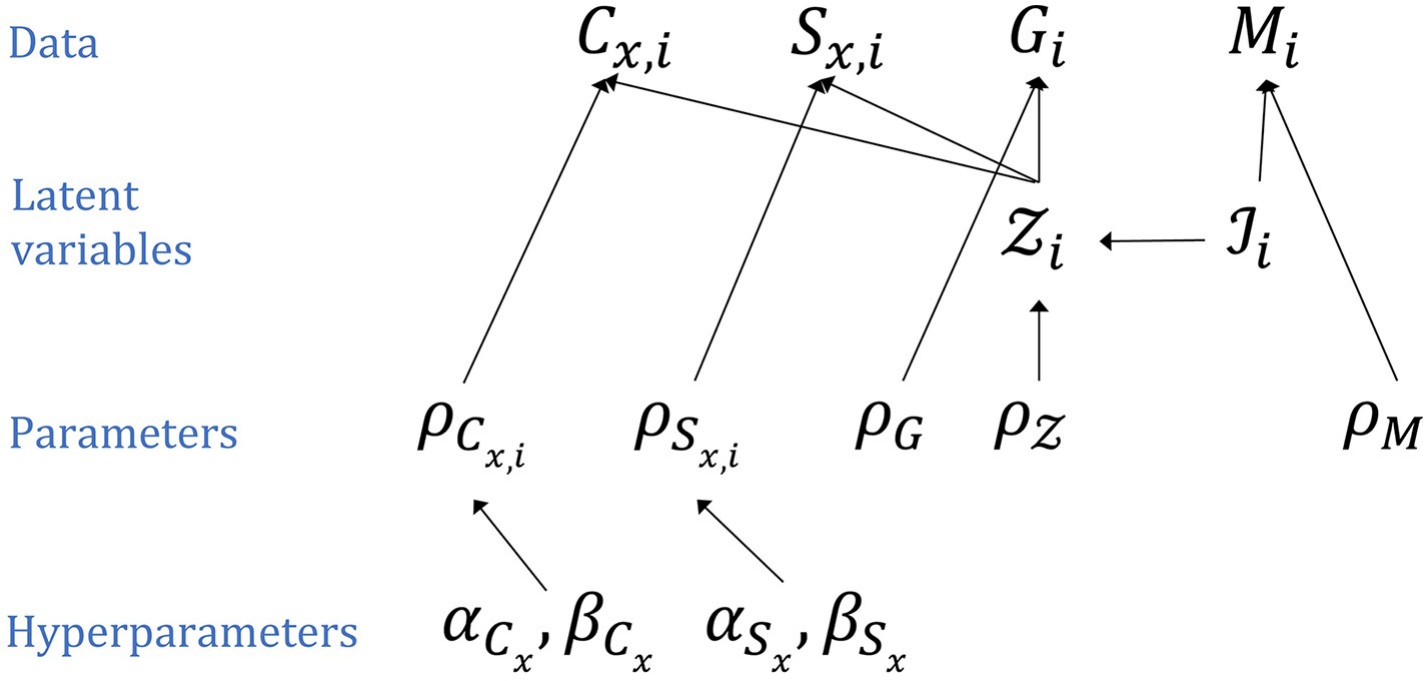
Model schematic. Model fitting was performed separately for each country or territory. The subscript *i* indicates administrative unit *i* within a modeled country. The subscript *x* represents either the total population (*x* = *T*) or pregnant women only (*x* = *P*). The top row represents the different data types (C = confirmed cases, S = suspected cases, G = Guillan-Barré syndrome cases, M = microcephaly cases). The second row includes the latent variables (Z = Symptomatic infections, and I = Infections). The third row includes the symptompatic probability ($\rho_{Z}$), the reporting probabilities for confirmed cases ($\rho_{C_{x,i}}$) and suspected cases($\rho_{S_{x}}$), the probability that a symptomatic infection leads to a reported GBS case (*ρ*_*G*_), and the probability that a ZIKV infection in a pregnant woman leads to a reported microcephaly case (*ρ*_*M*_). The parameters in the bottom row are the hyperparameters for the reporting probabilities $\rho_{C_{x,i}}$ and $\rho_{S_{x,i}}$. See text and S2 Table for description of model parameters and variables.

We estimated the national and subnational Zika IAR in each country using a hierarchical Bayesian model with the number of total infections and symptomatic infections treated as latent variables (Fig 1). Short descriptions for each of the model variables and parameters are presented in S2 Table. The model was run separately for each country because a single model would have contained over one thousand parameters, making it computationally prohibitive to simultaneously estimate parameters across all countries. Therefore, posterior parameter estimates for each country are independent of the estimates from the other countries. The IAR in a population of size *N*_*i*_ in administrative unit *i* is the proportion of the population that was infected, $\frac{I_{i}}{N_{i}}$, where $I_{i}$ is the number of infections. The number of symptomatic infections in a population, $Z_{i}$, depends on the size of the infected population, $I_{i}$, and the symptomatic probability, $\rho_{Z}$, resulting in $Z_{i}∼\text{Bin}\left(I_{i},\rho_{Z}\right)$. The number of confirmed Zika cases in the entire population (*T*) of administrative unit *i*, *C*_*T*,*i*_, depends on the number of symptomatic infections and the local reporting probability, $\rho_{C_{T,i}}$, resulting in $C_{T,i}∼\text{Bin}\left(Z_{i},\rho_{C_{T,i}}\right)$. The number of suspected Zika cases was similarly dependent on a reporting probability for suspected cases, $\rho_{S_{T,i}}$, and the number of symptomatic infections in the total population, $S_{T,i}∼\text{Bin}\left(Z_{i},\rho_{S_{T,i}}\right)$. Because misdiagnosis could contribute to the number of suspected cases during an epidemic, we also considered the possibility that there were more suspected cases than symptomatic infections by using a Poisson distribution rather than a binomial distribution to represent the reporting process for suspected cases. The results of this analysis are reported in the Supporting Information (S3 Appendix). The numbers of confirmed or suspected cases in pregnant women (*P*), *C*_*P*,*i*_ or *S*_*P*,*i*_, were represented by binomial distributions dependent on the number of symptomatic infections in pregnant women. The infection attack rate and probability of an infection being symptomatic in pregnant women were assumed to be the same as that in the entire population. The probabilities that a symptomatic infection was reported as a suspected or confirmed Zika case, $\rho_{S_{x}}$ and $\rho_{C_{x}}$, where *x* represents either the entire population (*T*) or pregnant women only (*P*), were assumed to differ between administrative units within a country or territory. To estimate this within-country variation in reporting probabilities, we assumed that the probability of a symptomatic infection being reported in administrative unit *i* followed a beta distribution with hyperparameters $\alpha_{Y_{x}}$ and $\beta_{Y_{x}}$ such that $\rho_{Y_{x,i}}∼\text{Beta}\left(\alpha_{Y_{x}},\beta_{Y_{x}}\right)$, where *Y* denotes confirmed (*C*) or suspected (*S*).

The number of reported Zika-associated GBS cases was dependent on the number of symptomatic infections, $G_{i}∼\text{Bin}\left(Z_{i},\rho_{G}\right)$, where *ρ*_*G*_ is the probability that a symptomatic infection results in a reported GBS case. The number of microcephaly cases associated with Zika was dependent on the total number of births in the population, *B*_*i*_, and the IAR, such that $M_{i}∼\text{Bin}\left(B_{i},\rho_{M}\frac{I_{i}}{N_{i}}\right)$, where *ρ*_*M*_ is the probability that an infection during pregnancy results in a reported microcephaly case.

Subnational IARs were estimated for each country and territory using available data types. The national-level IAR was calculated from the total number of subnational infections divided by the national population size, *N*, $\frac{\sum_{i=1}^{n}I_{i}}{N}$. For Puerto Rico, Zika IARs were estimated at the municipality-level (*n* = 78), due to availability of data at that scale. In addition, several datasets there were aggregated at the regional level (*n* = 8), due to their availability at that scale. For these data types, the regional-level IARs were estimated from the total number of infections within all municipalities in a given region.

#### Prior assumptions

The IAR reported in previous Zika outbreaks has been as high as 73% on the Micronesian island of Yap [21]. The IARs of *Aedes*-transmitted viruses in larger geographical areas tend to be lower than in smaller island environments, such as Yap, because spatial heterogeneity in the presence and abundance of *Aedes* limits transmission potential within a portion of the region [33]. Studies from several different Zika outbreaks have estimated basic reproduction numbers (*R*_0_) of 1.4 to 6.0 [34–38]. Based on the theoretical relationship between *R*_0_ and the final epidemic size [39], these *R*_0_ values would correspond to IARs of 0.286–0.833. However, IARs are typically lower at a given *R*_0_ value in populations with heterogeneous contact patterns [40], as is typical with transmission by *Ae*. *aegypti* mosquitoes [41]. To lightly constrain our ZIKV IAR estimates without precluding the possibility of values anywhere between 0 and 1, we used a Beta(1, 2) prior for the probability of an individual being infected (i.e., the IAR). This prior distribution had a median value of 0.292 (95% range: 0.013–0.842). We also performed an analysis with a uniform prior for the IARs. A comparison of posterior IAR estimates with and without the Beta(1, 2) prior is included in the Supporting Information (S3 Appendix).

Estimates of the symptomatic probability for Zika, $\rho_{Z}$, have varied considerably across studies [21, 22, 42]. One recent study estimated the symptomatic probability for three different locations (Yap Island, French Polynesia, and Puerto Rico), taking into account assay senstivity and specificity, as well as the possibility of Zika-like symptoms due to other causes [22]. Median estimates from that study ranged from 27% in Yap to 50% in Puerto Rico. To generate a single prior distribution for $\rho_{Z}$ in our model, we used the model and data provided in [22] to recreate the posterior estimates of $\rho_{Z}$ from their analysis, and then fitted a beta distribution to the combined posteriors using the ‘fitdistrplus’ package in R [43]. The resulting distribution was Beta(3.88, 5.34), which has a median of 0.41 and a 95% range of 0.14–0.73, and was used as a prior for each country. The hyperparameters $\alpha_{Y_{x}}$ and $\beta_{Y_{x}}$ for the reporting probabilities $\rho_{Y_{x}}$, where *Y* = *C* or *S* and *x* = *T* or *P*, were specified as Cauchy(0, 25) priors. This distribution provides a weakly informative prior with the distribution peaked at 0 and a long right tail. We assumed non-informative priors for the probabilities that a symptomatic infection results in a reported Guillan-Barré case or that an infected mother will give birth to a child with reported microcephaly. These probabilities represent not just the probability that an infection leads to a syndromic case, but also that such a case ends up being reported through the surveillance system.

#### Model implementation

Each country or territory model was fitted using the ‘rstan’ version 2.18.2 package in R using a Hamiltonian Monte Carlo algorithm (an MCMC variant) [44]. For each country or territory, four chains of 5,000 iterations each were run with a burn-in interval of 2,500 iterations. Convergence was assessed using the Gelman-Rubin convergence diagnostic [45]. The full model for Peru failed to converge, so the reporting probabilities of confirmed cases ($\rho_{C_{T}}$) and confirmed cases in pregnant women ($\rho_{C_{P}}$) were estimated as single parameters for all administrative units, rather than drawing $\rho_{C_{i}}$ from hyperparameters *α*_*C*_ and *β*_*C*_. We considered this simplification to be justifiable because all ZIKV confirmations were handled by either the national CNS laboratory or one of two regional laboratories, so confirmation rates likely did not vary as widely between administrative units as the reporting probabilities for suspected cases. Model diagnostic results for each country and territory are provided in S2 Appendix.

### Model validation

#### Simulation study

To assess the performance of our estimation method we used the median posterior estimates from Guatemala to simulate new case data for each of the different data types at the first administrative level. Three different simulated datasets were generated with the symptomatic probability ($\rho_{Z}$) set at either the median posterior estimate from Guatemala (3.2%), 25%, or 50%. We then used our statistical model to estimate the IAR and other parameters either with or without a Beta(2, 1) prior on the IAR parameters.

#### Posterior predictive checks

Posterior predictive checks were performed by comparing the empirical data to simulated data from the posterior parameter distributions. Posterior predictive data was generated at each iteration, *k*, of the MCMC, with $Y_{j}^{pre(k)}∼\text{Bin}\left(N_{j},\theta_{j}^{k}\right)$ for data type *j* ∈ {*C*_*T*_, *S*_*T*_, *C*_*P*_, *S*_*P*_, *M*, *G*} and its associated parameters *θ*_*j*_. At each iteration, the observed national total, *Y*_*j*_, was compared to the predicted national total, $Y_{j}^{pre(k)}$. This test statistic was used to calculate a Bayesian p-value $p_{B}=\text{Pr}\left(\left(Y_{j}^{pre},\theta_{j}\right)\geq \left(Y_{j},\theta_{j}\right)|Y_{j}\right)$, which indicates whether the distribution of the model-generated data was more extreme than the observed data [45]. We also compared the observed national totals for each data type to the predicted model output to determine whether the observed data (across all case types and territories) fell within the 95% credible interval (CrI) of the corresponding posterior distributions from the model.

#### Holdout analysis

To determine the sensitivity of model estimates to the inclusion of different data types, we fitted the model while holding out one data type at a time. This analysis was restricted to countries where all data types were available at a subnational level (Guatemala and Dominican Republic) or at most one data type was only available at the national-level (Panama). For these countries, we also fitted the model to one data type at a time to assess the benefit of using multiple data types in the estimation process.

#### Seroprevalence estimates

As an additional check, we compared modeled IAR estimates to published seroprevalence estimates from the Americas, as these quantities should be comparable. Seroprevalence estimates from at least one location were available for five countries or territories from ten published studies (S7 Table). Most of these studies used an NS1-based ELISA test for ZIKV IgG antibodies, and several confirmed all postive tests using plaque-reduction neutralization tests (PRNT) or flow cytometry neutralization tests (FRNT). For studies performing both ELISAs and PRNTs, we compared our estimates to the PRNT results. Two included studies did not test for ZIKV IgG: in Guayaquil, Ecuador Zambrano et al. [46] tested a control group of pregnant women for recent ZIKV infection via RT-PCR, and de Araújo [27] tested a control group of new mothers for ZIKV IgM with confirmation via PRNT in Recife, Brazil. The majority of these studies were conducted in a single city and not across a larger adminstrative area, in which case we compared the seroprevalence data to the IAR estimate from the first-level administrative unit where that city was located.

### Applying the model to settings with no spatially disaggregated data

Estimates from the 15 country-specific models were used to make predictions about IARs in the 33 other countries and territories in Latin America and the Caribbean where subnational data was not available. The January, 4 2018 Zika report from PAHO included cumulative data from 52 countries and territories in the Americas [3]. Canada, Bermuda, and Chile reported no locally-acquired cases, and the United States reported only a few hundred locally-acquired cases in a limited geographic area, so these countries were excluded from the analysis. For the remaining 48 countries and territories (including the 15 modeled territories), the cumulative numbers of confirmed cases, suspected cases, and cases of microcephaly were used to estimate the national IAR. For each of the 48 countries and territories, a national IAR estimate was obtained by drawing from the posterior distributions of the different reporting parameters from each of the 15 country models. This allowed us to draw from across the full range of estimated reporting probabilities from these 15 countries and territories in predicting the IARs in the remaining countries and territories. For a given country or territory model, *k*, the probability of a given IAR value in country or territory *j* was derived from the joint probability for each of the different data types (*C*_*j*_, *S*_*j*_, and/or *M*_*j*_) that were used to fit that model. The combined probability density function for IAR in country *j* was then taken as the sum of the probability density functions using the parameter estimates from all *K* = 15 models. As an alternative to drawing from all 15 modeled countries and territories to estimate infections in the non-modeled territories, we explored a second method where parameters were drawn only from a subset of the modeled countries that shared a border or similar characteristics (e.g., island nations) with the country or territory being estimated. Additional details on this estimation method are provided in S4 Appendix. These IAR estimates were used to calculate the total number of ZIKV infections that may have occurred during the epidemic. Initially, the infections arising in the 15 modeled territories were derived from country- or territory-specific model estimates and only the infections from the remaining 33 countries and territories were estimated from this pooled analysis. The territory-specific model estimates were compared to the pooled model estimates for each of the 15 modeled territories to assess the plausibility of the pooled estimates in the non-modeled countries and territories (S4 Appendix).

## Results

### Infection attack rate estimates

**Fig 2:**
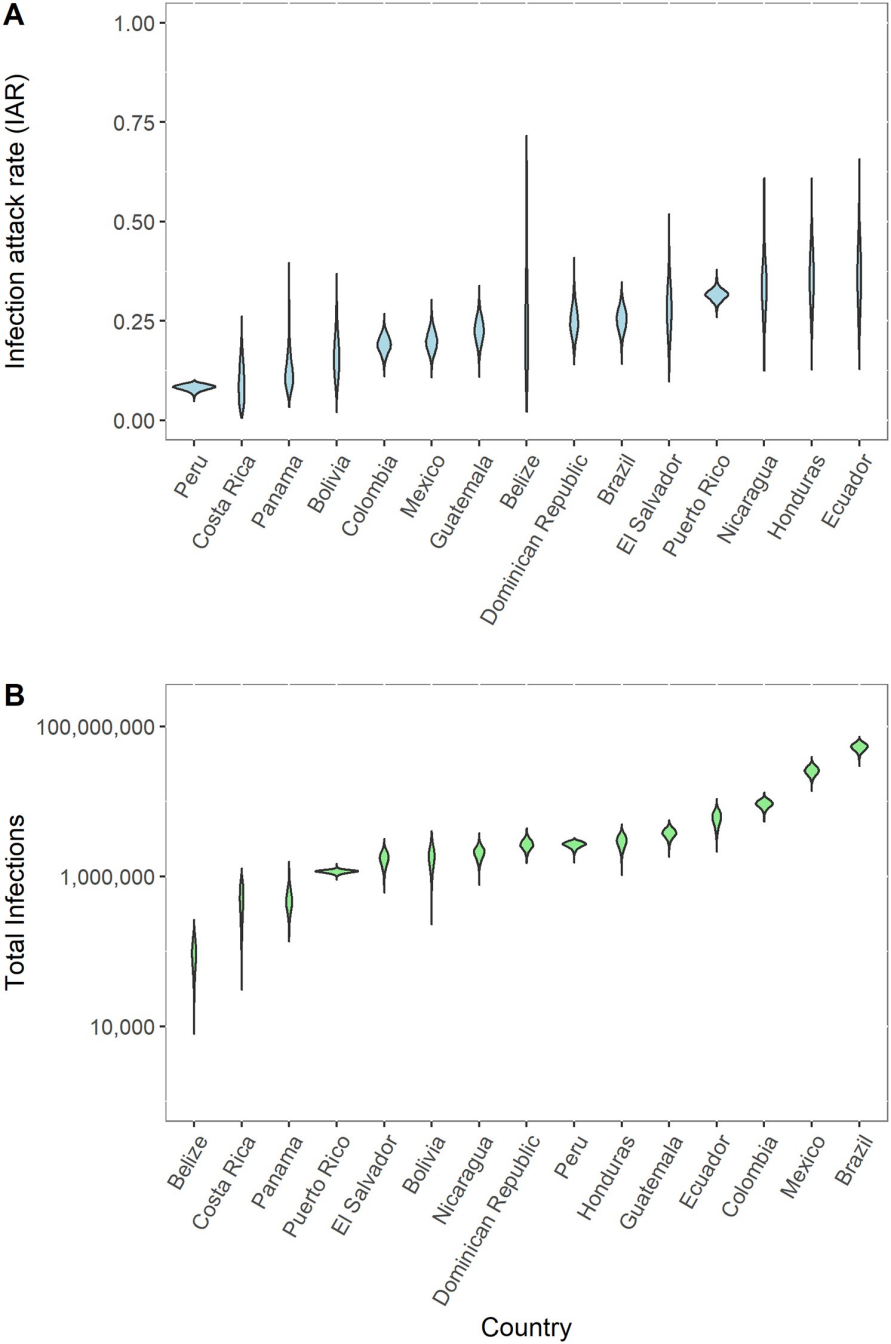
Posterior distributions of national ZIKV infection attack rate (IAR) and total ZIKV infections for 15 different countries and territories. (A) ZIKV IAR for each modeled country or territory ordered by median IAR. (B) Estimated number of ZIKV infections for each country or territory ordered by median number of infections.

**Fig 3:**
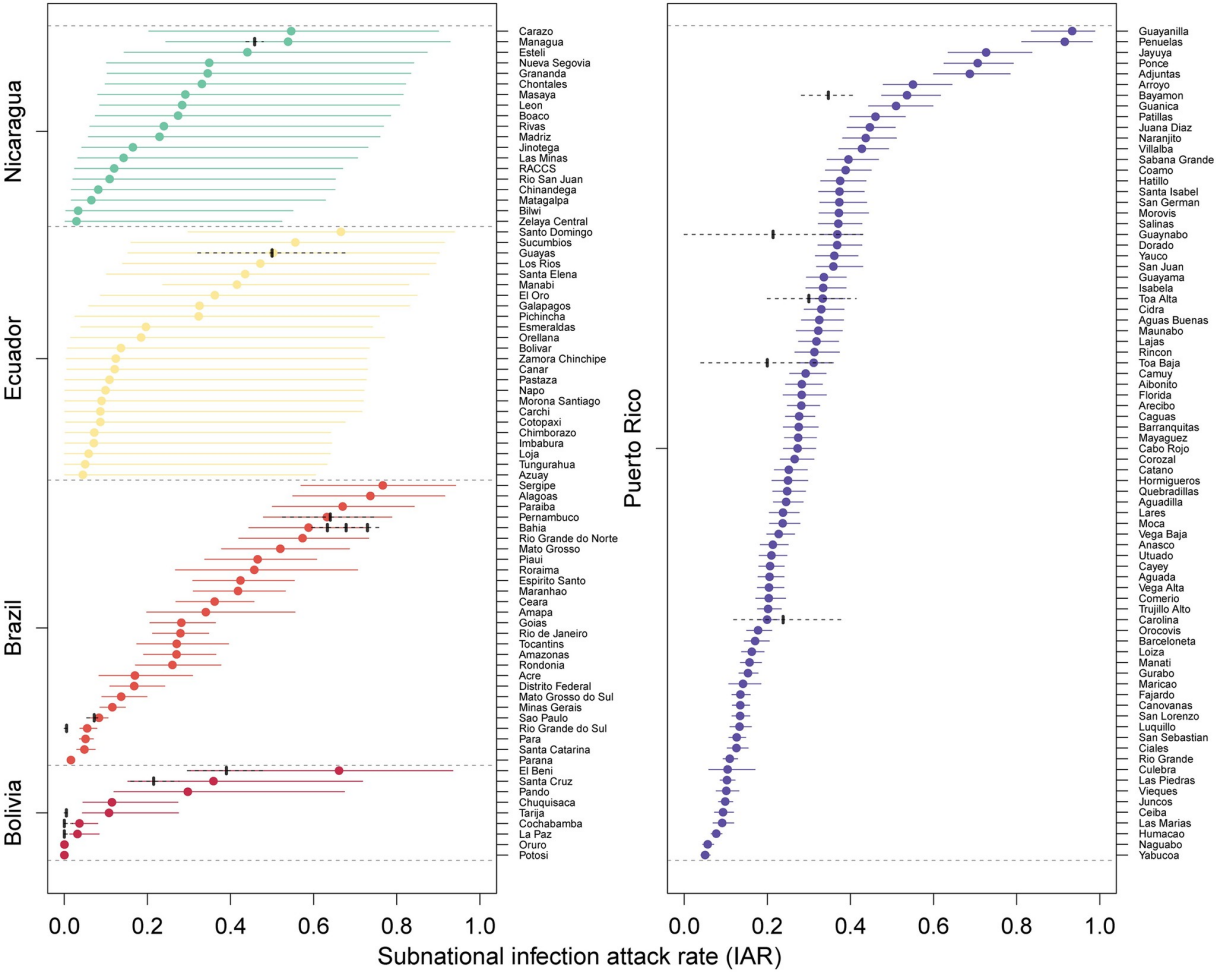
Posterior distribution of subnational ZIKV infection attack rates (IAR) for five different territories (Bolivia, Brazil, Ecuador, Nicaragua, and Puerto Rico). Colored circles and whiskers are the median and 95% credible intervals for each administrative unit. Black circles with dashed lines are seroprevalence estimates from the literature (see S7 Table). The dashed lines are the 95% confidence intervals for the seroprevalence estimates assuming a binomial distribution with the exception of the 95% CI estimate from [17] for Bahia, Brazil which was taken directly from their analysis.

Estimated Zika infection attack rates at the national level ranged from 0.084 (95% CrI: 0.067–0.096) in Peru to 0.361 (95% CrI: 0.214–0.514) in Ecuador (Fig 2A, S4 Table). There was considerable heterogeneity in IARs (Fig 3). In the most populous country, Brazil, the IAR at the subnational level varied from 0.016 (95% CrI: 0.01-0.025) in the State of Paraná to 0.766 (95% CrI: 0.569-0.942) in the State of Sergipe (Fig 3). In the second most populous country, Mexico, the IAR ranged from 0 (95% CrI: 0- 0) in the Federal District to 0.793 (95% CrI: 0.524-0.963) in the State of Yucatán (S17 Fig). Subnational IAR estimates for all 15 modeled countries and territories are presented in S7–S21 Figs.

Mean estimated IARs in countries and territories where subnational data were not available ranged from a low of 0.003 (95% CrI: 0.000-0.013) in Uruguay to a high of 0.979 (95% CrI: 0.591-1.000) in Saint Martin and 0.979 (95% CrI: 0.616-1.000) in Saint Barthélemy (S4 Table, S22–S25 Figs). Summing these IAR estimates across all countries and territories in the Americas, there were a total of 132.3 million (95% CrI: 111.3-170.2 million) ZIKV infections across Latin America and the Caribbean (Table 1). The majority of these infections, 114.1 million (95% CrI: 99.39-128.4 million), were from the 15 modeled countries and territories, while the other 33 countries and territories accounted for an additional 16.16 million (95% CrI: 5.427-51.71 million) infections. There were an estimated 53.4 million (95% CrI: 40.8-64.9 million) ZIKV infections in Brazil and 25.6 million (95% CrI: 19.2-32.7 million) in Mexico (Fig 2B). Venezuela had the largest number of ZIKV infections (6.63 million; 95% CrI: 0.35-31.5 million) out of the countries that were not explicitly modeled (S4 Table). The projected number of infections in non-modeled countries was lower under the alternative method of estimation based on only a subset of country-specific parameter estimates (Table 1).

**Table 1:** Total infections in modeled and projected countries and territories under two different projection methods. Default method used parameter estimates from all 15 modeled countries, while local method used only parameter estimates from neighboring countries and territories or those with similar characteristics.

	Default Projections	Local Projections
Modeled Countries (N = 15)	114,112,764	114,112,764
(95% CrI)	(99,394,311–128,358,749)	(99,394,311–128,358,749)
Non-modeled Countries (N = 33)	16,160,272	8,766,342
(95% CrI)	(5,426,679–51,709,310)	(4,162,037–35,907,308)
Total Infections	132,278,856	123,996,410
(95% CrI)	(111,305,999–170,156,510)	(108,124,733–151,195,526)

### Parameter estimates

**Fig 4:**
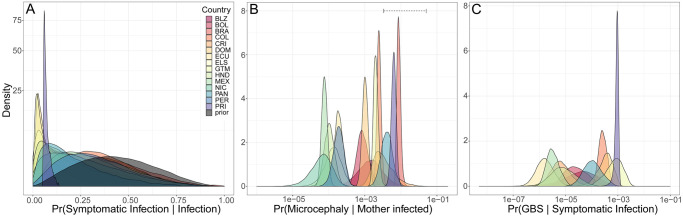
Posterior parameter estimates. (A) Posterior and prior symptomatic probability estimates for each country or territory. (B) Posterior estimates from each country and territory of the probability that a ZIKV infection in a pregnant woman results in a reported case of microcephaly. Dashed line represents range for estimated risk of Zika-associated microcephaly from published observational studies (see text for references). (C) Posterior estimates from each country and territory of the probability that a symptomatic infection results in a reported Guillan-Barré syndrome (GBS) case.

The median symptomatic probability of a ZIKV infection across all countries was 0.1 (95% CrI: 0.02-0.53), which was lower than the prior estimate of 0.41 (95% CrI: 0.14–0.73). The estimated symptomatic probability ranged from 0.03 (95% CrI: 0.01-0.17) in Guatemala to 0.33 (95% CrI: 0.15-0.6) in Colombia (Fig 4A). This shift in the posterior estimate relative to the prior estimate of the symptomatic probability may be the result of identifiability issues between the symptomatic probability and the reporting probability parameters for the different data types. Misdiagnosis of symptomatic ZIKV infections as a different disease, such as dengue fever, could also lower the estimate of the symptomatic probability, although dengue virus infections misidentified as ZIKV infections would have the opposite effect.

Reported rates of microcephaly in most countries were lower than recent estimates of the risk of microcephaly based on studies of ZIKV infection during pregnancy (Fig 4B). The probability that an infection in a pregnant woman resulted in a reported case of microcephaly ranged from a low of 0.07 per 1,000 infections (95% CrI: 0.01-0.19) in Nicaragua to 8.7 per 1,000 infections (95% CrI: 7.13-11.39) in Brazil. In comparison, estimates of the risk of microcephaly have range from 4.1 (95% CI: 3.4-4.9) per 1,000 [8] to 50 (95% CI: 40-70) per 1,000 births to ZIKV-infected mothers [47]. The probability that a symptomatic infection would result in a reported Guillan-Barré case varied from a low of 0.16 per 100,000 symptomatic infections (95% CrI: 0.04-0.7) in Ecuador to a high of 92.27 per 100,000 symptomatic infections (95% CrI: 55.72-113.6) in Puerto Rico (Fig 4C). The large variability between countries in reporting probabilities for microcephaly and GBS cases could be due to differences in case definitions among countries, differences in surveillance, or differences in underlying dengue immunity that may have impacted the severity of ZIKV infections due to some form of cross-reactive response [15–17].

The probability of a symptomatic infection being reported as a suspected or confirmed Zika case was higher for pregnant women than the general population for all countries where separate data on pregnant women were available, with the exception of suspected cases in El Salvador and Honduras (S26–S29 Figs). The countrywide reporting probability in pregnant women was as low as 0.002 (95% CrI: 0-0.012) for confirmed cases in El Salvador (S29 Fig, and as high as 0.289 (95% CrI: 0.041-0.989) for confirmed cases in Costa Rica and 0.276 (95% CrI: 0.056-0.727) for suspected cases in Dominican Republic (S28 Fig). As with the variability in reporting probabilities for GBS and microcephaly, this variability in confirming ZIKV infections in pregnant women indicates that there were considerable differences in surveillance and testing efforts among countries during the epidemic.

Countrywide reporting of suspected cases in the entire population ranged from 0.012 (95% CrI: 0.006-0.041) in Peru to 0.933 (95% CrI: 0.565-0.998) in Puerto Rico (S26 Fig). Countrywide reporting of confirmed cases ranged from 0.0004 (95% CrI: 0.0001-0.0023) in El Salvador to 0.504 (95% CrI: 0.305-0.54) in Puerto Rico (S27 Fig). The variation in reporting probabilities among administrative units within a country or territory was largest in Colombia for suspected cases (70.2% of variance was between administrative units vs. 29.8% due to within-unit variance), suspected cases in pregnant women (63.8%), and confirmed cases in pregnant women (67.7%). The largest between-administrative unit variance in reporting probabilities for confirmed cases in the total population occurred in Puerto Rico (56.6% of total variance). Several countries showed little variability in reporting probabilities among administrative units, with < 1% of total variance explained (S30–S33 Figs).

The posterior distribution of the national IAR was only weakly negatively correlated with the symptomatic probability posterior distribution in each country (S34 Fig). The IAR posterior distribution was not strongly correlated with the posterior distributions of any of the reporting probabilities, with the exception of the reporting probability for microcephaly cases, which is the only case type that we assumed could result from both asymptomatic and symptomatic infections (S35–S36 Figs). The posterior distributions of the other reporting rates were strongly correlated with the symptomatic probability posterior distribution (S37 Fig).

### Model validation

#### Simulation study

The modeled posterior IAR estimates closely matched the simulated IAR values when a Beta(1, 2) prior was used, but overestimated IAR when a flat prior was used (S38 Fig). The symptomatic probability ($\rho_{Z}$) was underestimated for each of the three different simulated values used with or without the Beta prior for IAR (S39 Fig).

#### Posterior predictive checks

**Fig 5:**
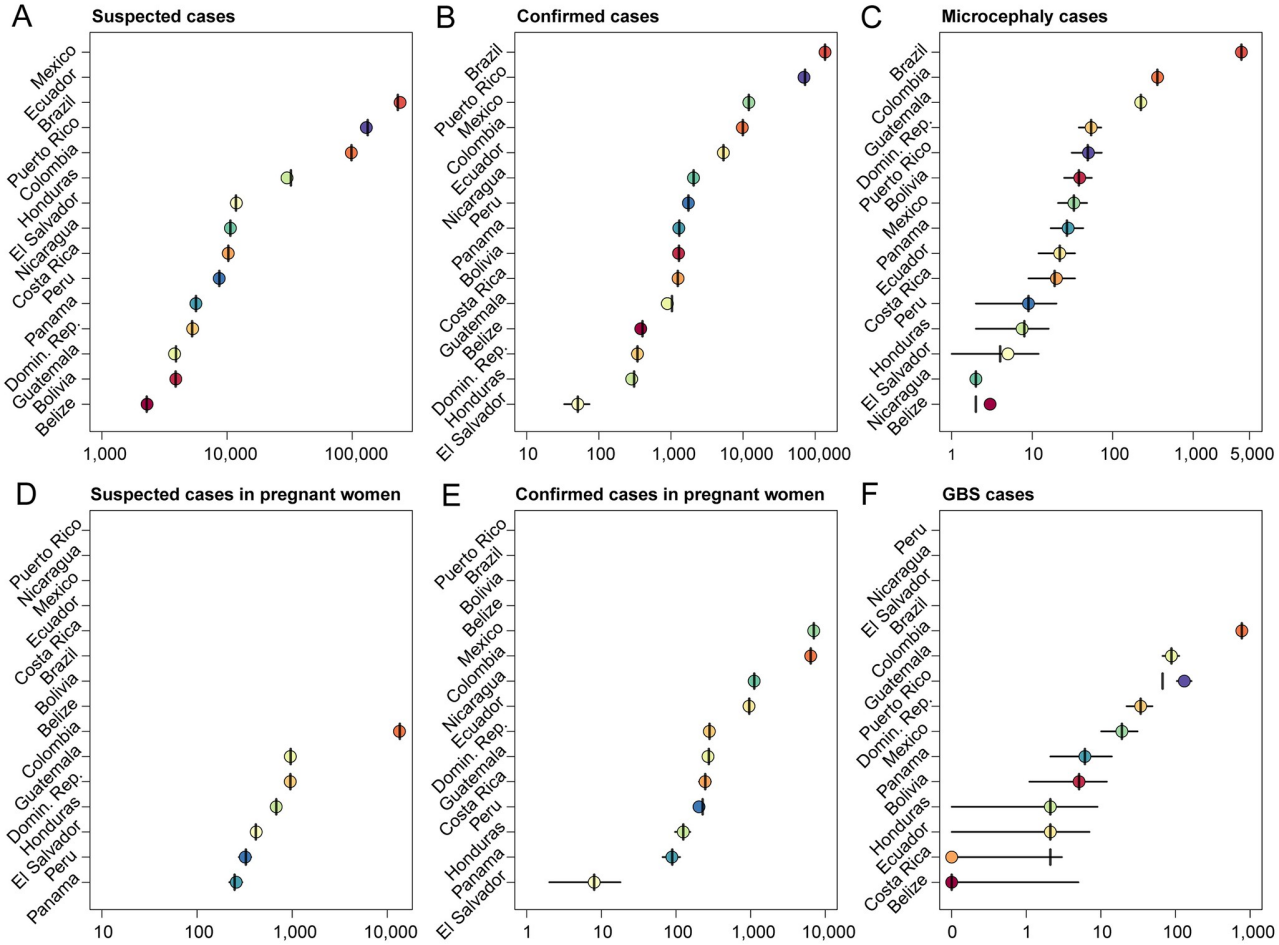
Posterior predictive checks at the national level for each data type used in the Bayesian models. Vertical lines are the observed cases and circles are the predicted number of cases with 95% credible intervals for each country and data type.

Our model did not generate predicted cases that were more extreme than the numbers of observed cases—as indicated by Bayesian p-values between 0.1 and 0.9—for each observed data type in all countries and territories except Puerto Rico. For Puerto Rico, the total number of reported confirmed cases (*p*_*B*_ = 0.990), suspected cases (*p*_*B*_ = 0.998), and GBS cases (*p*_*B*_ = 0.004), were outside the range of model predictions, but the number of microcephaly cases was not (*p*_*B*_ = 0.566) (Fig 5). The underestimation of confirmed and suspected cases—and overestimation of GBS cases in Puerto Rico indicates that our model was not capable of reconciling estimates based on different data types when the subnational reporting probabilities for these different data types did not vary consistently. Therefore, the municipality-level IAR estimates for Puerto Rico should be viewed with caution. The observed number of microcephaly cases, confirmed cases in pregnant women, and suspected Zika cases in pregnant women fell within the 95% posterior predictive interval (PPI) of the model for every country and territory that reported these data types (Fig 5). Beyond Puerto Rico, the number of confirmed cases in Guatemala was higher than the 95% PPI (1,032 vs. 811-963), the number of suspected cases in Honduras was higher than the 95% PPI (32,385 vs. 29,659-30,583), and the number of suspected cases in Brazil was lower than the 95% PPI (231,725 vs. 240,240-243,040). Comparisons of the observed data to subnational PPIs for each country or territory are provided in the project Github repository https://github.com/mooresea/Zika_IAR.

#### Holdout analysis

Modeled IAR estimates were not overly sensitive to any one data type. Removing any one data type from the estimation process did not significantly alter the national IAR estimate for Guatemala, with median estimates within 1.0% of the median estimate from the full model of 22.6% (S40 Fig). For the Dominican Republic, the median national IAR estimate varied from 0.217 to 0.314 depending on which data type was withheld, but these median model estimates were all within the 95% CrI for the full model of 0.180-0.331 (S41 Fig). For Panama, the national median estimates with different data types withheld were all slightly higher (0.127-0.150) than the median estimate of the full model (0.117), but still within the 95% CrI of 0.060-0.222 (S42 Fig). When models were fitted with a single data type, national and subnational IAR estimates varied widely depending on which data type was used to fit the model (S43–S45 Figs). The single-data-type models overestimated the median national IAR relative to the full model for all three countries or territories (Guatemala, Panama, and Puerto Rico), and had increased uncertainty at both the national and subnational levels.

#### Comparison to seroprevalence studies

Our median IAR estimates for Managua, Nicaragua (0.538; 95% CrI: 0.244-0.928) and Guayas, Ecuador (0.503; 95% CrI: 0.153-0.902) were close to seroprevalence estimates from those regions (Managua: 0.46, 95% CI: 0.44-0.48 [24]; Guayas:, 0.50, 95% CI:0.32-0.68 [46]) that were withheld from the fitting process (Fig 3). Overall, the 95% CI for 15 of 18 seroprevalence estimates from published studies overlapped with the 95% CrI of the IAR estimates from our model for the corresponding administrative unit. These two measures are not necessarily identical for every country or territory, because many of the seroprevalence studies were either conducted in only part of the administrative unit for which we estimated the IAR (e.g., the city of Salvador within Bahia State, Brazil or the city of Santos within São Paulo State, Brazil) or were conducted well before the end of the epidemic (Bolivia). The three estimates that did not overlap were Tarija, Bolivia (seroprevalence: 0.005; 95% CI: 0.000-0.015 vs. IAR: 0.108; 95% CrI: 0.043-0.275), Rio Grande do Sul, Brazil (seroprevalence: 0.006; 95% CI: 0.000-0.016 vs. IAR: 0.055; 95% CrI: 0.038-0.078), and Bayamon, Puerto Rico (seroprevalence: 0.347; 95% CI: 0.292-0.412 vs. IAR: 0.536; 95% CrI: 0.475-0.617).

## Discussion

Our results show that the vast majority of ZIKV infections in the Americas went unreported during the recent epidemic. PAHO reported fewer than one million suspected or confirmed Zika cases, whereas we estimate that 132.3 million (95% CrI: 111.3-170.2 million) people were infected. This discrepancy is due to the high number of asymptomatic infections, as well as estimated reporting probabilities below 1% for both suspected and confirmed Zika cases in many countries. The majority of ZIKV infections (114.1 million; 95% CrI: 99.39-128.4 million) occurred in the fifteen countries and territories that we modeled subnationally, while an additional 16.16 million (95% CrI: 5.427-51.71 million) occurred in the remaining American territories where national IAR estimates were extrapolated from the estimated reporting probabilities from the modeled countries. These estimates of the national and subnational IAR and total ZIKV infections represent the most extensive attempt to date to estimate the size of the Zika epidemic in the Americas.

Our results show significant within- and between-country variability in IARs. This large variability in IARs could reflect spatial heterogeneity in habitat suitability for *Ae*. *aegypti* [48]. Early projections of the epidemic also predicted that the IAR would be highly heterogeneous due to the geographic distribution of *Ae*. *aegypti* as well as other factors likely to influence transmission intensity, such as temperature and economic prosperity [49]. The historical pattern of virus introduction and spread may have also contributed to the high level of within- and between-country variability in IAR [50]. Local outbreaks in geographic regions or municipalities that began near the end of the local transmission season may have ended before epidemic fadeout would be expected due to herd immunity [10, 51]. Determining the extent to which this spatial heterogeneity is the result of ZIKV epidemiology versus historical contingencies will be important for identifying current and future at-risk populations.

Although our IAR estimates closely matched estimates from seroprevalence studies in some areas (e.g., Managua, Nicaragua, Guayas, Ecuador, and Pernambuco, Brazil), other estimates diverged from published seroprevalence estimates. One potential reason for the discrepancy between our IAR estimates and serosurvey-based estimates is a mismatch in the spatial scale of the region that the IAR estimate covers. For example, our estimate for the Brazilian state of Bahia (0.514; 95% CrI: 0.388-0.631), was slightly lower than the seroprevalence estimates of 0.63 (95% CI: 0.595-0.671),0.68 (95% CI: 0.600-0.744), and 0.73 (95% CI: 0.70–0.76) from Salvador, the largest city in Bahia [17, 25, 26]. However, the overall IAR in Bahia might be lower than the IAR in Salvador if rural areas outside of Salvador experienced a lower infection attack rate. Serosurvey-based estimates might also underestimate IAR if the sensitivity and specificity of the serological assay are not taken into account. Only Rodriguez-Barraquer et al. [17] reported seroprevalence estimates adjusted for assay sensitivity and specificity, which increased their estimate from 0.63 (95% CI: 0.60-0.65) to 0.73 (95% CI: 0.70-0.76) for Salvador, Brazil. Another potential cause of discrepancies is that several of the serosurveys were conducted prior to completion of the ZIKV epidemic. For example, the published seroprevalence estimates for Bolivia from Villarroel et al. [52] were conducted from December, 2016 until May, 2017, but Bolivia had significant ZIKV transmission activity throughout 2017 and 2018. As a result, our IAR estimates which incorporated data from 2017 and 2018 were higher than those reported by Villarroel et al. [52]. Conducting further serosurveys in a range of locations across the Americas would be an important step in determining current levels of immunity and would further validate our model estimates.

Posterior predictive checks showed that Puerto Rico was the only territory for which our model did not accurately predict several of the observed data types, which may explain why our IAR estimate for Bayamon, Puerto Rico was significantly different than the seroprevalence estimate from Lozier et al. [53]. Puerto Rico has more administrative level-one areas (78) than the other modeled countries and territories, which suggests that our model may produce overly precise estimates when there is a large sample size and reporting probabilities are too variable between different administrative areas to be reconciled. Even with variable reporting probabilities among administrative levels, confirmed cases were underestimated in some administrative areas of Puerto Rico and overestimated in others. In addition, variation in the reporting probabilities of different case types was not always spatially consistent (e.g., certain areas had above-average reporting of suspected cases, but below average reporting of confirmed cases or GBS cases).

The estimated reporting probabilities for microcephaly in each territory were significantly lower than the reported risk of microcephaly or CZS during pregnancy from several different studies. However, the primary goal of our analysis was to estimate the number of ZIKV infections and not to estimate individual epidemiological parameters such as the symptomatic probability or the risk of Zika-associated microcephaly. Our microcephaly detection estimates are significantly lower than estimates from epidemiological studies because we are estimating both the probability of a ZIKV infection resulting in microcephaly, and the probability that a microcephaly case was diagnosed and reported. Our estimates also depended on estimates of the annual number of births in a region, but this could represent an overestimate of the number of births at risk in many regions because the duration of the epidemic in most localities was less than one year. In addition, differences in the outbreak length among areas would lead to different estimates of the microcephaly reporting probability due to differences in the denominator (number of susceptible births) rather than the underlying risk of microcephaly. Overestimating the number of births at risk would lead to an underestimate of the microcephaly rate in a region or country. Whether this underestimate in the microcephaly rate would also affect the estimate of IAR would depend on the influence of the other data types on the total likelihood. The risk of microcephaly or CZS has been estimated as 0.95% (95% CI: 0.34-1.91%) during the first trimester in French Polynesia [54], 2.3% (2 out of 86 births) in Colombia [55], and 5% (95% CI: 4-7%) among fetuses and infants born to women with laboratory-confirmed ZIKV infection in the U.S. territories [47]. In a cohort study in Rio de Janerio, 42% of children born to ZIKV-infected mothers were found to have central nervous system abnormalities, although only 4 of 117 were diagnosed with microcephaly [5]. Our estimates ranged from 0.1 per 1,000 infections (95% CrI: 0-0.2) in Nicaragua to 8.7 per 1,000 infections (95% CrI: 7.1-11.4) in Brazil. Changes in the reporting probabilities of both ZIKV infections and microcephaly over the course of the epidemic could also complicate the estimation of the true risk of CZS [56].

Rates of GBS also varied significantly, ranging from 0.04 to 8.51 per 100,000 total ZIKV infections. A recent review estimated a rate of 20 GBS cases per 100,000 ZIKV infections (95% CrI: 5-45 per 100,000), which is higher than our estimates [57]. However, that analysis also estimated the IAR for each location included in their study and estimated lower IARs for Bahia, Brazil (0.02), Salvador, Brazil (0.08), Colombia (0.09), El Salvador (0.15), and Honduras (0.04) than our model. Their IAR estimates for Bahia State and Salvador in Brazil were also much lower than seroprevalence estimates from three studies [17, 25, 26] that more closely matched our IAR estimates for Bahia State. Underestimating the number of ZIKV infections would lead to an overestimate of GBS incidence per infection. For Puerto Rico, where our estimate for IAR (0.316; 95% CrI: 0.284–0.347) is within the 95% CrI of the IAR estimate (0.17; 0.08-0.46) by [57], our estimate of 8.51 (95% CrI: 6.94-11.15) GBS cases per 100,000 ZIKV infections is also within the range of their estimate of 14 (95% CrI: 4-25) GBS cases per 100,000 ZIKV infections. This overlap suggests that our method of simultaneously estimating IAR, reporting probabilities, and the risk of GBS produces reasonable estimates for GBS risk and reporting.

The current status of population-level immunity throughout the Americas has important implications for the future of Zika epidemiology in the region [10, 11, 58]. Our IAR estimates suggest that a number of areas exceeded the herd immunity threshold, which has also been suggested by other studies [19]. However, the high degree of heterogeneity in subnational IARs suggests that certain areas in Central and South America that are suitable for ZIKV transmission may still contain a considerable number of susceptible individuals. While some areas experienced a low IAR because of environmental conditions that limited local transmission, such as high-elevation locations in Colombia and Peru, there are other areas that had a low IAR where conditions appear favorable for ZIKV transmission. For example, the Brazilian states of Pará and Amazonas had median IAR estimates of 0.077 and 0.164, even though both were believed to be high-risk locations [49, 59] based on high suitability for *Ae*. *aegypti* [48, 60] and historical dengue incidence [61, 62]. If the remaining population-at-risk is large in these areas, then local outbreaks in the near future could still be possible. Modeling studies also suggest that the presence of multiple locations with insufficient immunity to reach the herd immunity threshold increases the chances that ZIKV becomes endemic in the region in the next decade as new births replenish the susceptible population [10]. Although Zika incidence in the Americas in 2019 appears to be much lower than during the epidemic, limited transmission has been reported in multiple countries and recent Zika outbreaks have also been reported in Africa and Asia [63], leaving open the possibility that ZIKV could be re-introduced into vulnerable populations. However, it is also possible that there was significant transmission in these states prior to the detection of ZIKV transmission in the Americas, in which case the actual IAR could be higher than we estimate. Genomic data suggests that ZIKV was present in northeast Brazil by early 2014 and spread to multiple countries before Zika surveillance began [64]. Microcephaly data can reconstruct some of this missed portion of the epidemic due to the lag between maternal infection and birth, but even this data will miss infections that occurred more than a year prior to the first case reports.

Because of the high variation in estimated reporting probabilities among modeled countries, there was a high degree of uncertainty in our projections of IAR in the non-modeled territories. Previous studies have also found high variation in the probability of a ZIKV infection being reported between countries [19]. This resulted in a fair amount of uncertainty in our estimates of the total number of ZIKV infections (95% CrI: 111, 305, 999-170, 156, 510) in a total population of 623.7 million. However, because most of the non-modeled territories were relatively small in population size (the total estimated population size in the 15 modeled territories is 507.1 million versus 116.6 million in the 33 non-modeled territories) the median estimate is largely a reflection of infections in the modeled countries. Another limitation of projecting IARs from the modeled countries to non-modeled countries and territories is that smaller islands and overseas territories such as Saint Martin, Guadeloupe, French Guiana, and Martinique likely had better surveillance and higher reporting rates than any of the modeled countries. For example, we projected an attack rate of 1.00 (95% CrI: 0.23-1.00) or 0.59 (95% CrI: 0.41-1.00) in French Guiana, but a recent study by Flamand et al. [65] found an estimated seroprevalence of 0.233 (95% CI: 0.209-0.259), suggesting that their surveillance system detected a higher fraction of infections than most, if not all, of the countries we analyzed. Several overseas territories Because we were unable to precisely estimate IAR without estimating territory-specific reporting probabilities, the publication of subnational totals for different types of Zika cases (suspected, microcephaly, etc.) would help greatly in refining these estimates. The publication of this data in conjunction with seroprevalence data would be particularly valuable for examining differences in surveillance systems and the geographic variation in IAR throughout the entire region [10].

The Zika epidemic in the Americas was a major public health concern that drew considerable attention from both the public health sector and the general public. Despite this attention, the majority of infections were not detected by public health surveillance systems. Using a hierarchical Bayesian model with empirically-informed priors, we were able to leverage Zika case reporting to simultaneously estimate national and subnational reporting probabilities, the fraction of symptomatic infections, and subnational IARs. Our results indicate that fewer than 1% of ZIKV infections in the Americas were reported during the recent epidemic. In the absence of detailed seroprevalence data, our subnational and national IAR estimates are an important first attempt at assessing current levels of ZIKV immunity throughout the Americas. Current levels of immunity are a critical determinant of the probability of further Zika outbreaks—and potentially the likelihood of dengue outbreaks as well—in this region in the next decade.

## Supporting information

S1 Appendix Zika data. (PDF)

S2 Appendix Model implementation and diagnostics. (PDF)

S3 Appendix Testing model assumptions. (PDF)

S4 Appendix Infection attack rate projections. (PDF)

S1 Table Summary of data availability at the national or subnational level for each country and territory. (PDF)

S2 TableDescription of each of the variables and parameters used in the models.Parameters were estimated separately for each model country or territory. Where available, the variables were provided at the 1st administrative unit for a particular country. The symptomatic probability ($\rho_{Z}$), the fraction of (*ρ*_*G*_) and (*ρ*_*G*_) were estimated at the country-level. The reporting probabilities for confirmed and suspected cases in the total population and in pregnant women were estimated at the 1st-administrative unit level, using the detailed hyperparameters to capture within-country variation in these reporting probabilities.(PDF)

S3 Table Comparison of national IAR estimates with or without a beta prior for IAR. (PDF)

S4 Table Infection attacks rates and total infections for default projections based on parameter estimates from all 15 national models. (PDF)

S5 Table Infection attacks rates and total infections based on parameter estimates from a subset of national models as indicated by the Models column. (PDF)

S6 Table Comparison of model-specific IAR estimates for each modeled territory versus projections of IAR from combined model estimates under the two different projection methods. (PDF)

S7 Table Zika seroprevalence studies. (PDF)

S1 Fig Traceplots of the log probability density of the posterior distribution for each country model after warmup. Colors represent the four separate chains.
(TIF)

S2 Fig Comparison of posterior national-level IAR estimates for each country or territory with or without a Beta(1, 2) prior for subnational IARs. (TIF)

S3 Fig Comparison of posterior estimates from four countries of the probability that a symptomatic infection was reported as a suspected case when the reporting process was modeled using a binomial or Poisson distribution. (TIF)

S4 Fig Comparison of posterior national-level IAR estimates for four countries when the reporting process for suspected cases was modeled using a binomial or Poisson distribution. (TIF)

S5 Fig Comparison of posterior national-level IAR estimates for four countries when the reporting process for suspected cases was modeled using a binomial or Poisson distribution. (TIF)

S6 Fig Comparison of posterior national-level IAR estimates for four countries when the reporting process for suspected cases was modeled using a binomial or Poisson distribution. (TIF)

S7 Fig Posterior distribution of subnational infection attack rates (IAR) for Belize. (TIF)

S8 Fig Posterior distribution of subnational infection attack rates (IAR) for Bolivia. (TIF)

S9 Fig Posterior distribution of subnational infection attack rates (IAR) for Brazil. (TIF)

S10 Fig Posterior distribution of subnational infection attack rates (IAR) for Colombia. (TIF)

S11 Fig Posterior distribution of subnational infection attack rates (IAR) for Costa Rica. (TIF)

S12 Fig Posterior distribution of subnational infection attack rates (IAR) for the Dominican republic. (TIF)

S13 Fig Posterior distribution of subnational infection attack rates (IAR) for Ecuador. (TIF)

S14 Fig Posterior distribution of subnational infection attack rates (IAR) for El Salvador. (TIF)

S15 Fig Posterior distribution of subnational infection attack rates (IAR) for Guatemala. (TIF)

S16 Fig Posterior distribution of subnational infection attack rates (IAR) for Honduras. (TIF)

S17 Fig Posterior distribution of subnational infection attack rates (IAR) for Mexico. (TIF)

S18 Fig Posterior distribution of subnational infection attack rates (IAR) for Nicaragua. (TIF)

S19 Fig Posterior distribution of subnational infection attack rates (IAR) for Panama. (TIF)

S20 Fig Posterior distribution of subnational infection attack rates (IAR) for Peru. (TIF)

S21 Fig Posterior distribution of subnational infection attack rates (IAR) for Puerto Rico. (TIF)

S22 Fig Probability distribution of the infection attack rate (IAR) for the Bahamas assuming reporting probabilities estimated from (A) all 15 different national models or (B) a subset of national models based on similarities in the country or territories. The different colors represent the probability distribution of IAR generated from using the estimated reporting probabilities from each modeled territory.
(TIF)

S23 Fig Probability distribution of the infection attack rate (IAR) for Venezuela assuming reporting probabilities estimated from (A) all 15 different national models or (B) a subset of national models based on similarities in the country or territories. The different colors represent the probability distribution of IAR generated from using the estimated reporting probabilities from each modeled territory.
(TIF)

S24 Fig Probability distribution of the infection attack rate (IAR) for the British Virgin Islands assuming reporting probabilities estimated from (A) all 15 different national models or (B) a subset of national models based on similarities in the country or territories. The different colors represent the probability distribution of IAR generated from using the estimated reporting probabilities from each modeled territory.
(TIF)

S25 Fig Probability distribution of the infection attack rate (IAR) for the U.S. Virgin Islands assuming reporting probabilities estimated from (A) all 15 different national models or (B) a subset of national models based on similarities in the country or territories. The different colors represent the probability distribution of IAR generated from using the estimated reporting probabilities from each modeled territory.
(TIF)

S26 Fig Posterior estimates from each country and territory of the probability that a symptomatic ZIKV infection is reported as a suspected Zika case. (TIF)

S27 Fig Posterior estimates from each country and territory of the probability that a symptomatic ZIKV infection is reported as a confirmed Zika case. (TIF)

S28 Fig Posterior estimates from each country and territory of the probability that a symptomatic ZIKV infection in a pregnant woman is reported as a suspected Zika case. (TIF)

S29 Fig Posterior estimates from each country and territory of the probability that a symptomatic ZIKV infection in a pregnant woman is reported as a confirmed Zika case. (TIF)

S30 Fig Posterior estimates from each country and territory of the probability that a symptomatic ZIKV infection is reported as a suspected Zika case. Black lines show the country-wide average reporting probability and grey lines show the estimated reporting probability in each administrative unit.
(TIF)

S31 Fig Posterior estimates from each country and territory of the probability that a symptomatic ZIKV infection is reported as a confirmed Zika case. Black lines show the country-wide average reporting probability and grey lines show the estimated reporting probability in each administrative unit.
(TIF)

S32 Fig Posterior estimates from each country and territory of the probability that a symptomatic ZIKV infection in a pregnant woman is reported as a suspected Zika case. Black lines show the country-wide average reporting probability and grey lines show the estimated reporting probability in each administrative unit.
(TIF)

S33 Fig Posterior estimates from each country and territory of the probability that a symptomatic ZIKV infection in a pregnant woman is reported as a confirmed Zika case. Black lines show the country-wide average reporting probability and grey lines show the estimated reporting probability in each administrative unit.
(TIF)

S34 Fig Joint posterior distributions for infection attack rate (IAR) and symptomatic probability from each country and territory. (TIF)

S35 Fig Joint posterior distributions for infection attack rate (IAR) and different reporting probabilities for Colombia. (TIF)

S36 Fig Joint posterior distributions for infection attack rate (IAR) and microcephaly reporting probability from each country and territory. (TIF)

S37 Fig Joint posterior distributions for symptomatic probability and different reporting probabilities for Colombia. (TIF)

S38 Fig Posterior distributions of ZIKV infection attack rates (IAR) estimated from simulated data. Simulation numbers are the three different simulated datasets with different symptomatic probability values. Blue values are posterior distributions from model using a Beta(1, 2) prior, and red values are posterior distributions from model with a flat prior. The solid lines are the simulated IAR value being estimated.
(TIF)

S39 FigPosterior distributions of the symptomatic probability ($\rho_{Z}$) estimated from simulated data.Simulation numbers are the three different simulated datasets with different $\rho_{Z}$ values. Blue values are posterior distributions from model using a Beta(1, 2) prior, and red values are posterior distributions from model with a flat prior. The solid lines are the simulated $\rho_{Z}$ value being estimated.(TIF)

S40 Fig Posterior distributions of national and subnational ZIKV infection attack rates (IAR) for Guatemala when each of the different data types is withheld from the model fitting process. (TIF)

S41 Fig Posterior distributions of national and subnational ZIKV infection attack rates (IAR) for the Dominican Republic when each of the different data types is withheld from the model fitting process. (TIF)

S42 Fig Posterior distributions of national and subnational ZIKV infection attack rates (IAR) for Panama when each of the different data types is withheld from the model fitting process. (TIF)

S43 Fig Posterior distributions of national and subnational ZIKV infection attack rates (IAR) for Guatemala from models fit to a single data type in comparison to the full model fit to all data types. (TIF)

S44 Fig Posterior distributions of national and subnational ZIKV infection attack rates (IAR) for the Dominican Republic from models fit to a single data type in comparison to the full model fit to all data types. (TIF)

S45 Fig Posterior distributions of national and subnational ZIKV infection attack rates (IAR) for Panama from models fit to a single data type in comparison to the full model fit to all data types. (TIF)
