# Supplementary material for: Leveraging multiple data types to estimate the size of the Zika epidemic in the Americas
Source: PLoS Negl Trop Dis. 2020 Sep 28;14(9):e0008640. doi: 10.1371/journal.pntd.0008640 (PMC7544039; doi:10.1371/journal.pntd.0008640)
Supplement: S1 Appendix — (PDF) [file pntd.0008640.s001.pdf]

Appendix S1. Zika data

We collected publicly available Zika data for the 15 countries or territories where we were able to find subnational case counts for at least one data type (S1 Table). The potential data types considered were confirmed Zika cases (either lab or clinically confirmed), suspected Zika cases, microcephaly cases associated with a ZIKV infection in the mother, and Zika-associated cases of Guillan-Barré syndrome (GBS). Many territories also reported the number of suspected or confirmed Zika cases in pregnant women. Where available, these counts were treated as independent observations from the population-wide observations. Although cases in pregnant women and in the entire population are treated as independent data points, we assume that the infection attack rate (IAR) is the same in both populations. A graphical representation of how IAR was estimated from the observed data is provided in Fig (1) of the main text. Short descriptions for each of the variables and parameters included in the model are presented in S2 Table.

Where available, we obtained Zika data at the first administrative level (e.g., province or state) within a country or territory. Where lower level data was available, it was aggregated to the first administrative level. The majority of datasets were obtained either from governmental websites or from country reports produced by PAHO [28]. Details on the time period covered by each dataset and data sources are provided in S1 Table. Where data totals were provided by epidemiological week, the start and end dates for these reporting periods were obtained from the governmental report or assumed to match the dates of WHO epidemiological weeks. Although we list the start and end periods of reporting for each data type, in some instances there may have been gaps in reporting during this period.
