# Supplementary material for: Leveraging multiple data types to estimate the size of the Zika epidemic in the Americas
Source: PLoS Negl Trop Dis. 2020 Sep 28;14(9):e0008640. doi: 10.1371/journal.pntd.0008640 (PMC7544039; doi:10.1371/journal.pntd.0008640)
Supplement: S2 Appendix — (PDF) [file pntd.0008640.s002.pdf]

Appendix S2. Model implementation and diagnostics

Each country or territory model was fitted using the 'rstan' version 2.18.2 package in R [44] using the 'No-U-turn' sampling algorithm with four chains of 5,000 iterations and a 50% burn-in period. Smaller step sizes for the sampling algorithm were set by increasing the adapt delta parameter from the default of 0.8 to 0.99. In addition, the maximum tree depth was increased from 10 to 15. Convergence was assessed using the Gelman-Rubin convergence diagnostic, R_c [45]. The R_c values for every parameter in each of the 15 county or territory models were < 1.05, indicating that each model achieved convergence. Traceplots of the log of the posterior distribution are provided in S1 Fig.

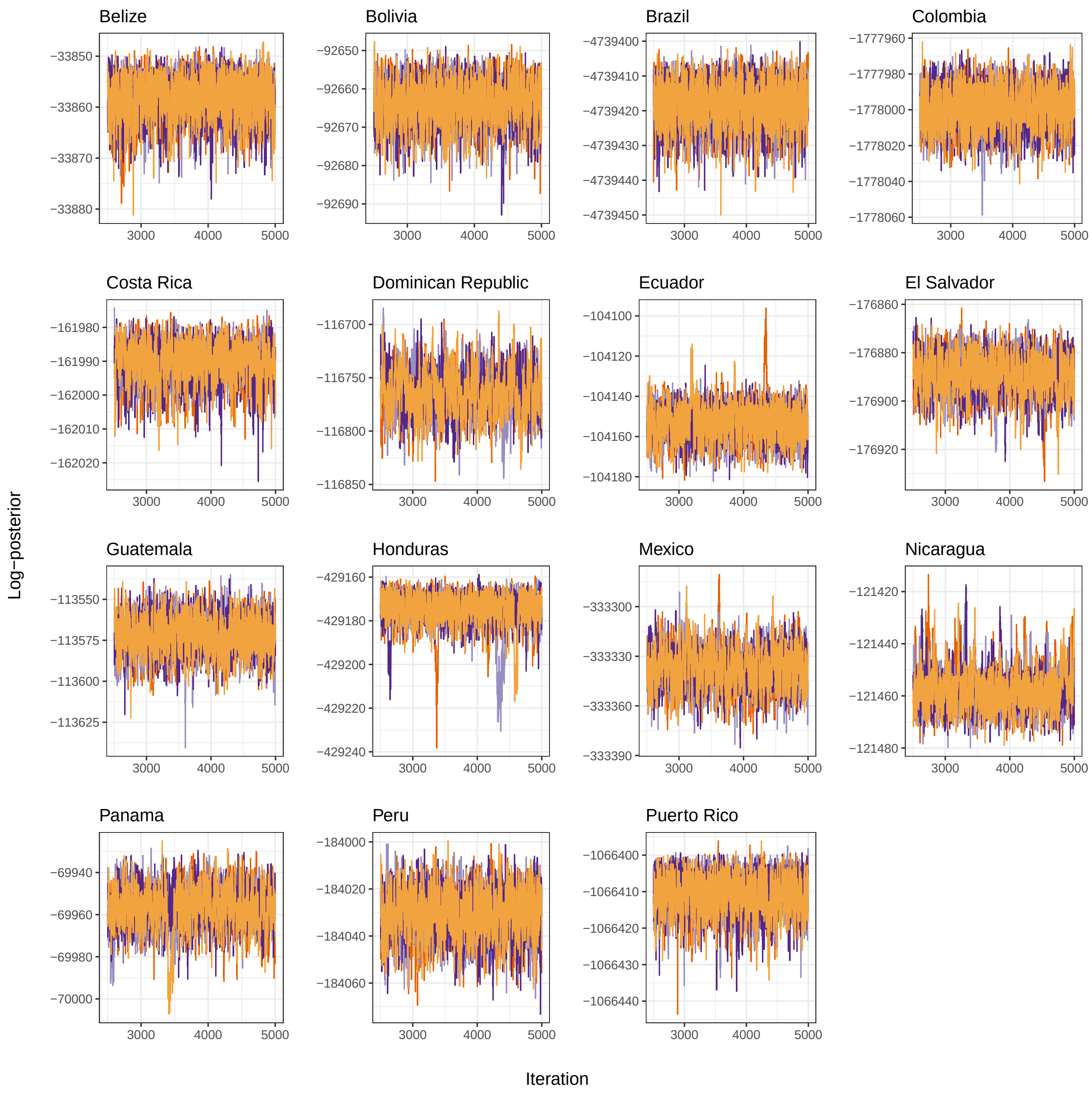

SI Fig 1: Traceplots of the log probability density of the posterior distribution for each country model after warmup. Colors represent the four separate chains.
