# Supplementary material for: Leveraging multiple data types to estimate the size of the Zika epidemic in the Americas
Source: PLoS Negl Trop Dis. 2020 Sep 28;14(9):e0008640. doi: 10.1371/journal.pntd.0008640 (PMC7544039; doi:10.1371/journal.pntd.0008640)
Supplement: S3 Appendix — (PDF) [file pntd.0008640.s003.pdf]

Appendix S3. Testing model assumptions

In the analysis presented in the main text, we used a Beta(1, 2) prior for the IAR in each country or territory model. The intention of this prior was to lightly constrain our ZIKV IAR estimates and prevent the model from converging towards extreme estimates without precluding the possibility of values anywhere between 0 and 1. This prior distribution has a median value of 0.292 (95% range: 0.013–0.842). To examine the sensitivity our IAR estimates to this prior assumption, we also ran a model version for each territory with a uniform prior on the IAR. With the uniform prior for the subnational IARs, the posterior IAR estimates at both the subnational and national level were higher for all 15 modeled countries and territories except for Costa Rica (S3 Table; S2 Fig).

In the analysis presented in the main text, we assumed that the reporting of symptomatic ZIKV infections, $\mathcal{Z}_i$, as suspected cases, S_i, followed a binomial distribution, $S_{T,i} \sim \text{Bin}(\mathcal{Z}_i, \rho_{S_{T,i}})$. The probability of a symptomatic infection being reported as a suspected case, $\rho_{S_{T,i}}$, in administrative unit i of a country or territory followed a beta distribution with hyperparameters α_{S_T} and β_{S_T}. However, because there is considerable overlap between the symptoms of a ZIKV infection and the symptoms of several other arbovirus infections—including dengue and chikungunya—the number of suspected Zika cases could exceed the number of symptomatic ZIKV infections if other arbovirus infections were misdiagnosed as ZIKV during the epidemic. To account for this possibility, we also considered a model where $S_{T,i} \sim \text{Poisson}(\mathcal{Z}_i \rho_{S_{T,i}})$. The reporting probability for suspected cases was allowed to range above one by drawing from a gamma distribution, $\rho_{S_{T,i}} \sim \text{Gamma}(\alpha_{S_T}, 1/\beta_{S_T})$. The gamma distribution hyperparameters, α_{S_T} and β_{S_T}, were assigned truncated standard normal prior distributions. These hyperparameter priors result in a mean of $\alpha_{S_T}\beta_{S_T} = 0.64$, and a variance of $\alpha_{S_T}\beta_{S_T}^2 = 0.512$ for the prior of the gamma distribution.

The version of the model with a Poisson distribution for suspected cases was run for Costa Rica, Guatemala, Panama, and Puerto Rico. These four countries and territories represented the range of estimated suspected reporting probabilities that were observed for the model with a binomial distribution, with Guatemala and Panama having relatively low estimates of ρ_{S_T},

and Costa Rica and Puerto Rico having the second highest and highest estimates of ρ_{S_T}, respectively. The values of ρ_{S_T} did not vary significantly between the models with binomial or Poisson distributions for Panama (0.092; 95% CrI: 0.024-0.436 vs. 0.074; 95% CrI: 0.021-0.343) or Guatemala (0.027; 95% CrI: 0.001-0.194 vs. 0.040; 95% CrI: 0.0022-0.268) (S3 Fig). In addition, the IAR estimates for these two countries differed by <1% (S4 Fig). The median estimate of ρ_{S_T} for Costa Rica was lower with the Poisson distribution (0.14; 95% CrI: $0.029-0.768$) than with the binomial distribution (0.255; 95% CrI: $0.037-0.908$) (S3 Fig). This decrease in the estimated reporting probability was only associated with a small increase in the IAR estimate from 0.092 (95% CrI: $0.019-0.193$) to 0.102 (95% CrI: $0.026-0.206$) (S4 Fig - S5 Fig). Puerto Rico had the highest estimate of ρ_{S_T} when suspected cases were binomially distributed, with a median of 0.933 and a upper 95% credible interval very close to 1 (95% CrI: $0.632-0.999$). When we assumed suspected cases followed a Poisson distribution, the median estimate of ρ_{S_T} was 0.299 (95% CrI: $0.099-0.958$). The marginal posterior distribution for ρ_{S_T} with a Poisson distribution was much broader than with the binomial distribution, and although the 95% credible interval was below 1 the upper estimates from the posterior were greater than 1 (S3 Fig). The estimated territory-wide IAR in Puerto Rico was higher with a Poisson distribution (0.38; 95% CrI: $0.325-0.437$) than with the binomial distribution (0.316; 95% CrI: $0.288-0.345$) (S4 Fig). A majority of Puerto Rico's municipalities had higher IAR estimates with a Poisson distribution, but several estimates were lower than they were with a binomial distribution (S6 Fig).
