# Supplementary material for: Leveraging multiple data types to estimate the size of the Zika epidemic in the Americas
Source: PLoS Negl Trop Dis. 2020 Sep 28;14(9):e0008640. doi: 10.1371/journal.pntd.0008640 (PMC7544039; doi:10.1371/journal.pntd.0008640)
Supplement: S4 Appendix — (PDF) [file pntd.0008640.s004.pdf]

Appendix S4. Infection attack rate projections

The territory-level IARs and number of ZIKV infections for all of the Americas were estimated by extrapolating parameter estimates from the 15 modeled countries and territories to the remaining countries and territories in the region. ZIKV IAR and the total number of ZIKV infections were estimated for the 33 countries and territories that reported confirmed Zika cases (C), suspected Zika cases (S), and Zika-associated microcephaly cases (M) to the Pan American Health Organization (PAHO) [3]. A national IAR estimate was obtained by drawing from the posterior distributions of the different reporting parameters from each of the 15 country models. This allowed us to draw from across the full range of estimated reporting probabilities from these 15 countries and territories in predicting the IARs in the remaining territories (S6 Table). For a given model, k, the probability of a given IAR value in country j was derived from the joint probability of the probability density functions for each of the different data types (C_j, S_j, and/or M_j) that were used to fit that model. The IAR, $\frac{\mathcal{I}_{j,k}}{N_j}$, for country j from model k given C_j was estimated using a binomial distribution $C_j \sim \text{Bin}(N_j, \rho_{C_{j,k}} \rho_{\mathcal{Z}_k} \frac{\mathcal{I}_{j,k}}{N_j})$, where $\rho_{C_{j,k}}$ was drawn from $\rho_{C_{j,k}} \sim \text{Beta}(\alpha_{C_k}, \beta_{C_k})$ using the posterior distributions for α_{C_k} and β_{C_k} from model k. Similarly, $S_j \sim \text{Bin}(N_j, \rho_{S_{j,k}} \rho_{\mathcal{Z}_k} \frac{\mathcal{I}_{j,k}}{N_j})$ and $M_j \sim \text{Bin}(B_j, \rho_{M_i} \frac{\mathcal{I}_{j,k}}{N_j})$. These probability densities were calculated across a range of $\frac{\mathcal{I}_{j,k}}{N_j}$ from 0 to 1 based on 1,000 draws from the posterior distribution of model k. The combined probability density function for $\frac{\mathcal{I}_j}{N_j}$ across all $K = 15$ models was $\frac{\mathcal{I}_j}{N_j} = \sum_{k=1}^{K} (\mathcal{I}_{j,k} | \Theta_j) / K N_j$.

As an alternative to drawing from all 15 modeled countries and territories to estimate infections in the non-modeled territories, we explored a second method where parameters were drawn only from a subset of the modeled countries that shared a border or similar characteristics (e.g., island nations) with the country or territory being estimated. Projections for mainland countries in Central and South America were estimated using the parameters of the nearest 2-4 neighbors, including all bordering countries. Projections for island countries in the Caribbean were estimated using parameters from Puerto Rico, Dominican Republic, and Belize under the assumption that reporting probabilities in smaller island nations would be similar to other islands or countries with a population of under one million (Belize). Finally, projections for the

overseas territories of France, the United Kingdom, the United States, and the Netherlands were estimated using parameter estimates from Puerto Rico under the assumption that these territories would have similar surveillance systems. The models used to estimate infections for each country and territory is presented in S5 Table.

Examples of the IAR probability distributions under both projection methods are presented in S22 Fig - S25 Fig. The IAR probability distributions for the remaining countries and territories are located in the Github repository `https://github.com/mooresea/Zika_IAR`. The median IAR estimates and total number of ZIKV infections in each country and territory under the default method are provided in S4 Table and estimates for the alternative method are provided in S5 Table. For the 15 modeled territories, these estimates use the modeled IAR estimates rather than IAR projections based on the range of reporting probabilities observed across all 15 territories. A comparison of the modeled IAR estimates versus the IAR projections for each of these territories is provided in S6 Table. The 95% CrIs for the projected IAR estimates include the mean of the model posterior for the national-level IAR for all 15 modeled territories. However, under the default method the 95% CrI for the projections of most territories is very broad, with a lower bound near 0 and an upper bound at 1. These broad credible intervals are an inevitable result of the high variability in estimated reporting probabilities among the modeled territories, which results in a high uncertainty in IARs when sampling reporting probabilities from across the posterior distributions from every model. The median projected IAR is very similar to the modeled IAR estimate for several countries (e.g., Brazil, Honduras, Nicaragua), but drastically different for several other territories, including Belize and Puerto Rico. These results suggest that the projections for non-modeled territories should be interpreted cautiously. Fortunately, most of the non-modeled countries have small populations and do not contribute significantly to the continent-wide estimate of ZIKV infections.
