# Supplementary material for: Leveraging multiple data types to estimate the size of the Zika epidemic in the Americas
Source: PLoS Negl Trop Dis. 2020 Sep 28;14(9):e0008640. doi: 10.1371/journal.pntd.0008640 (PMC7544039; doi:10.1371/journal.pntd.0008640)
Supplement: S1 Table — (PDF) [file pntd.0008640.s005.pdf]

SI Table 1: Summary of data availability at the national or subnational level for each country and territory.

Country	Data Type	Available	Level	Start Date	End Date	Source
Brazil	Suspected cases	T	1	4/2015	12/31/2018	[66]
	Confirmed cases	T	0	4/2015	1/4/2018	[3]
	Pregnant suspected	F	-	-	-	-
	Pregnant confirmed	F	-	-	-	-
	Microcephaly	T	1	11/8/2015	12/31/2018	[67]
	Guillain-Barré	F	-	-	-	-
Bolivia	Suspected cases	T	1	1/1/2016	6/30/2018	[68]
	Confirmed cases	T	1	1/1/2016	11/3/2018	[69]
	Pregnant suspected	F	-	-	-	-
	Pregnant confirmed	F	-	-	-	-
	Microcephaly	T	1	1/1/2016	6/30/2018	[68]
	Guillain-Barré	T	1	1/1/2016	6/30/2018	[68]
Belize	Suspected cases	T	1	1/1/2016	12/31/2017	fn. 1
	Confirmed cases	T	1	1/1/2016	12/31/2017	fn. 1
	Pregnant suspected	F	-	-	-	-
	Pregnant confirmed	F	-	-	-	-
	Microcephaly	T	1	1/1/2016	12/31/2017	fn. 1
	Guillain-Barré	T	1	1/1/2016	12/31/2017	fn. 1
Colombia	Suspected cases	T	1	8/9/2015	12/31/2017	[70]
	Confirmed cases	T	1	8/9/2015	12/31/2017	[70]
	Pregnant suspected	T	1	8/9/2015	12/31/2017	[70]
	Pregnant confirmed	T	1	8/9/2015	12/31/2017	[70]
	Microcephaly	T	0	1/1/2016	12/31/2018	[70]
	Guillain-Barré	T	1	10/18/2015	12/31/2018	[70]
Costa Rica	Suspected cases	T	0	1/1/2016	9/29/2018	[71, 72, 73]
	Confirmed cases	T	1	1/1/2016	12/31/2017	[71, 72, 73]
	Pregnant suspected	F	-	-	-	-

	Pregnant confirmed	T	1	1/1/2016	9/29/2018	[71, 73, 74]
	Microcephaly	T	0	1/1/2016	9/29/2018	[73, 75, 76]
	Guillain-Barré	T	0	1/1/2016	9/29/2018	[71, 72, 73]
Dominican Republic	Suspected cases	T	1	1/1/2016	3/25/2017	[77]
	Confirmed cases	T	1	1/1/2016	3/25/2017	[77]
	Pregnant suspected	T	1	1/1/2016	3/25/2017	[77]
	Pregnant confirmed	T	1	1/1/2016	3/25/2017	[77]
	Microcephaly	T	1	1/1/2016	3/25/2017	[77]
	Guillain-Barré	T	1	1/1/2016	3/25/2017	[77]
Ecuador	Suspected cases	F	-	-	-	-
	Confirmed cases	T	1	12/20/2015	10/23/2018	[78]
	Pregnant suspected	F	-	-	-	-
	Pregnant confirmed	T	1	12/20/2015	10/23/2018	[78]
	Microcephaly	T	1	12/20/2015	10/23/2018	[78]
	Guillain-Barré	T	1	12/20/2015	10/23/2018	[78]
El Salvador	Suspected cases	T	1	11/22/2015	12/16/2017	[79, 80]
	Confirmed cases	T	0	11/22/2015	12/31/2016	[80]
	Pregnant suspected	T	1	11/22/2015	12/16/2017	[79, 80]
	Pregnant confirmed	T	0	1/3/2016	12/31/2016	[80]
	Microcephaly	T	0	11/22/2015	8/6/2016	[81]
	Guillain-Barré	F	-	-	-	-
Guatemala	Suspected cases	T	1	11/29/2015	10/20/2018	[82]
	Confirmed cases	T	1	11/29/2015	11/11/2017	[82]
	Pregnant suspected	T	1	1/1/2016	11/26/2016	[82]
	Pregnant confirmed	T	1	1/1/2016	10/20/2018	[82]
	Microcephaly	T	1	1/1/2016	11/18/2017	[82]
	Guillain-Barré	T	1	1/1/2016	11/11/2017	[82]
Honduras	Suspected cases	T	1	1/1/2016	12/31/2017	[83]
	Confirmed cases	T	0	12/13/2015	1/4/2018	[3]
	Pregnant suspected	T	0	1/1/2016	8/19/2017	[84]
	Pregnant confirmed	T	0	1/1/2016	8/19/2017	[84]
	Microcephaly	T	0	12/13/2015	8/19/2017	[84]

	Guillain-Barré	T	0	1/1/2016	8/19/2017	[84]
Mexico	Suspected cases	F	-	-	-	-
	Confirmed cases	T	1	11/28/2015	11/5/2018	[85]
	Pregnant suspected	F	-	-	-	-
	Pregnant confirmed	T	1	11/28/2015	11/5/2018	[86]
	Microcephaly	T	1	11/28/2015	9/17/2018	[86]
	Guillain-Barré	T	1	11/28/2015	2/6/2018	[86]
Nicaragua	Suspected cases	T	0	1/27/2016	12/31/2017	[87]
	Confirmed cases	T	1	1/27/2016	12/31/2017	[88]
	Pregnant suspected	F	-	-	-	-
	Pregnant confirmed	T	0	1/27/2016	1/2/2017	[89]
	Microcephaly	T	0	1/27/2016	11/21/2016	[89]
	Guillain-Barré	F	-	-	-	-
Panama	Suspected cases	T	0	11/22/2015	9/25/2017	[90]
	Confirmed cases	T	1	11/22/2015	11/10/2018	[91]
	Pregnant suspected	T	1	11/22/2015	11/10/2018	[91]
	Pregnant confirmed	T	1	11/22/2015	11/10/2018	[91]
	Microcephaly	T	1	11/22/2015	11/10/2018	[91]
	Guillain-Barré	T	1	11/22/2015	11/10/2018	[91]
Peru	Suspected cases	T	1	1/1/2016	9/15/2018	[92]
	Confirmed cases	T	1	1/1/2016	4/14/2018	[93]
	Pregnant suspected	F	-	-	-	-
	Pregnant confirmed	T	1	1/1/2016	12/31/2017	[94]
	Microcephaly	T	0	1/1/2016	9/25/2017	[95]
	Guillain-Barré	F	-	-	-	-
Puerto Rico	Suspected cases	T	2	1/1/2016	1/7/2017	[96]
	Confirmed cases	T	2	1/1/2016	1/7/2017	[96]
	Pregnant suspected	F	-	-	-	-
	Pregnant confirmed	F	-	-	-	-
	Microcephaly	T	0	1/1/2016	12/30/2017	[97]
	Guillain-Barré	T	1	1/1/2016	1/7/2017	[96]

[1]Belize MoH; http://health.gov.bz/www/contact-us
