# Supplementary material for: Leveraging multiple data types to estimate the size of the Zika epidemic in the Americas
Source: PLoS Negl Trop Dis. 2020 Sep 28;14(9):e0008640. doi: 10.1371/journal.pntd.0008640 (PMC7544039; doi:10.1371/journal.pntd.0008640)
Supplement: S2 Table — Parameters were estimated separately for each model country or territory. Where available, the variables were provided at the 1st administrative unit for a particular country. The symptomatic probability (ρZ), the fraction of (ρG) and (ρG) were estimated at the country-level. The reporting probabilities for confirmed and suspected cases in the total population and in pregnant women were estimated at the 1st-administrative unit level, using the detailed hyperparameters to capture within-country variation in these reporting probabilities. (PDF) [file pntd.0008640.s006.pdf]

SI Table 2: Description of each of the variables and parameters used in the models. Parameters were estimated separately for each model country or territory. Where available, the variables were provided at the 1st administrative unit for a particular country. The symptomatic probability ($\rho_{\mathcal{Z}}$), the fraction of (ρ_G) and (ρ_G) were estimated at the country-level. The reporting probabilities for confirmed and suspected cases in the total population and in pregnant women were estimated at the 1st-administrative unit level, using the detailed hyperparameters to capture within-country variation in these reporting probabilities.

Variable or Parameter	Description
$\mathcal{I}$	Number of infected individuals
$\mathcal{Z}$	Number of symptomatic infections
S_T	Number of suspected cases in total population
C_T	Number of confirmed cases in total population
S_P	Number of suspected cases in pregnant women
C_P	Number of confirmed cases in pregnant women
G	Number of Guillan-Barré syndrome (GBS) cases
M	Number of microcephaly cases
$\rho_{\mathcal{Z}}$	Proportion of infections that are symptomatic
ρ_{S_T}	Fraction of symptomatic infections that are reported as a suspected case
ρ_{C_T}	Fraction of symptomatic infections reported as a confirmed case
ρ_{S_P}	Fraction of symptomatic infections in pregnant women that are reported as a suspected case
ρ_{C_P}	Fraction of symptomatic infections in pregnant women that are reported as a confirmed case
ρ_G	Fraction of symptomatic infections that result in a reported GBS case
ρ_M	Fraction of infections in pregnant women that result in a reported microcephaly case
α_{S_T}	Hyperparameter from beta distribution for ρ_{S_T}
β_{S_T}	Hyperparameter from beta distribution for ρ_{S_T}
α_{C_T}	Hyperparameter from beta distribution for ρ_{C_T}
β_{C_T}	Hyperparameter from beta distribution for ρ_{C_T}
α_{S_P}	Hyperparameter from beta distribution for ρ_{S_P}
β_{S_P}	Hyperparameter from beta distribution for ρ_{S_P}
α_{C_P}	Hyperparameter from beta distribution for ρ_{C_P}
β_{C_P}	Hyperparameter from beta distribution for ρ_{C_P}
