# Supplementary material for: Leveraging multiple data types to estimate the size of the Zika epidemic in the Americas
Source: PLoS Negl Trop Dis. 2020 Sep 28;14(9):e0008640. doi: 10.1371/journal.pntd.0008640 (PMC7544039; doi:10.1371/journal.pntd.0008640)
Supplement: S3 Table — (PDF) [file pntd.0008640.s007.pdf]

SI Table 3: Comparison of national IAR estimates with or without a beta prior for IAR.

Country	IAR w/o prior	95% CrI	IAR w/ prior	95% CrI
Belize	0.46	(0.15 - 0.74)	0.23	(0.07 - 0.49)
Bolivia	0.23	(0.12 - 0.38)	0.16	(0.07 - 0.29)
Brazil	0.27	(0.23 - 0.3)	0.25	(0.19 - 0.31)
Colombia	0.26	(0.21 - 0.31)	0.19	(0.15 - 0.23)
Costa Rica	0.02	(0.01 - 0.13)	0.09	(0.02 - 0.19)
Dominican Republic	0.35	(0.26 - 0.45)	0.25	(0.18 - 0.33)
Ecuador	0.53	(0.34 - 0.69)	0.36	(0.21 - 0.51)
El Salvador	0.40	(0.27 - 0.53)	0.28	(0.16 - 0.4)
Guatemala	0.29	(0.23 - 0.35)	0.23	(0.16 - 0.29)
Honduras	0.55	(0.4 - 0.68)	0.36	(0.22 - 0.49)
Mexico	0.28	(0.22 - 0.33)	0.20	(0.15 - 0.25)
Nicaragua	0.50	(0.35 - 0.65)	0.33	(0.21 - 0.46)
Panama	0.17	(0.08 - 0.35)	0.12	(0.06 - 0.22)
Peru	0.59	(0.44 - 0.64)	0.08	(0.07 - 0.1)
Puerto Rico	0.43	(0.35 - 0.5)	0.32	(0.29 - 0.35)
