# Supplementary material for: Leveraging multiple data types to estimate the size of the Zika epidemic in the Americas
Source: PLoS Negl Trop Dis. 2020 Sep 28;14(9):e0008640. doi: 10.1371/journal.pntd.0008640 (PMC7544039; doi:10.1371/journal.pntd.0008640)
Supplement: S4 Table — (PDF) [file pntd.0008640.s008.pdf]

SI Table 4: Infection attacks rates and total infections for default projections based on parameter estimates from all 15 national models.

Country	IAR	95% CrI	Infections	95% CrI
Brazil	0.25	(0.19 - 0.31)	53,388,206	(40,819,653 - 64,922,428)
Mexico	0.20	(0.15 - 0.25)	25,599,729	(19,237,945 - 32,690,560)
Colombia	0.19	(0.15 - 0.23)	9,302,116	(7,133,364 - 11,360,866)
Venezuela	0.21	(0.01 - 1)	6,633,999	(350,008 - 31,518,000)
Ecuador	0.36	(0.21 - 0.51)	5,956,506	(3,523,747 - 8,484,736)
Guatemala	0.23	(0.16 - 0.29)	3,772,441	(2,714,844 - 4,819,782)
Honduras	0.36	(0.22 - 0.49)	2,926,168	(1,824,954 - 4,044,226)
Peru	0.08	(0.07 - 0.1)	2,672,621	(2,129,464 - 3,054,319)
Dominican Republic	0.25	(0.18 - 0.33)	2,657,916	(1,923,663 - 3,538,936)
Nicaragua	0.33	(0.21 - 0.46)	2,061,039	(1,320,432 - 2,841,540)
Bolivia	0.16	(0.07 - 0.29)	1,769,432	(793,925 - 3,172,282)
El Salvador	0.28	(0.16 - 0.4)	1,717,254	(974,790 - 2,482,731)
Puerto Rico	0.32	(0.29 - 0.35)	1,164,139	(1,059,133 - 1,270,456)
Trinidad and Tobago	0.62	(0.01 - 1)	847,316	(9,648 - 1,366,851)
Jamaica	0.28	(0.02 - 1)	787,408	(47,988 - 2,808,000)
Suriname	0.90	(0.04 - 1)	495,564	(21,279 - 548,000)
Guadeloupe	1.00	(0.34 - 1)	472,000	(158,442 - 472,000)
Panama	0.12	(0.06 - 0.22)	465,004	(238,134 - 886,204)
Costa Rica	0.09	(0.02 - 0.19)	448,074	(94,060 - 942,369)
Martinique	1.00	(0.09 - 1)	396,000	(34,131 - 396,000)
French Guiana	1.00	(0.23 - 1)	275,970	(64,676 - 276,000)
Haiti	0.02	(0 - 1)	272,412	(6,580 - 10,910,543)
Cuba	0.02	(0 - 0.98)	192,194	(2,322 - 11,193,532)
Curacao	1.00	(0.28 - 1)	148,974	(41,318 - 149,000)
Barbados	0.43	(0.02 - 1)	124,633	(5,303 - 291,947)
Argentina	0.00	(0 - 0.73)	122,880	(4,448 - 32,221,973)
Aruba	1.00	(0.11 - 1)	113,850	(11,981 - 114,000)
Virgin Islands (US)	1.00	(0.13 - 1)	102,910	(12,980 - 103,000)
Saint Lucia	0.62	(0.04 - 1)	102,142	(5,781 - 164,984)

Belize	0.23	(0.07 - 0.49)	85,497	(24,747 - 181,433)
Saint Vincent and the Grenadines	0.82	(0.04 - 1)	83,376	(3,914 - 101,986)
Paraguay	0.01	(0 - 0.99)	74,808	(4,172 - 6,638,558)
Dominica	1.00	(0.11 - 1)	73,802	(7,838 - 74,000)
Grenada	0.58	(0.03 - 1)	64,499	(2,770 - 110,971)
Bahamas	0.16	(0.01 - 1)	61,828	(3,222 - 394,768)
Antigua and Barbuda	0.65	(0.03 - 1)	60,924	(3,163 - 93,985)
Saint Kitts and Nevis	0.98	(0.06 - 1)	51,950	(3,397 - 52,996)
Guyana	0.06	(0 - 0.98)	48,304	(545 - 757,640)
Sint Maarten (Dutch part)	0.99	(0.06 - 1)	41,465	(2,441 - 41,994)
Cayman Islands	0.63	(0.03 - 1)	36,408	(1,554 - 57,974)
Saint Martin	1.00	(0.57 - 1)	35,989	(20,419 - 36,000)
Turks and Caicos Islands	0.57	(0.03 - 1)	29,828	(1,459 - 51,969)
Bonaire, St Eustatius and Saba	1.00	(0.16 - 1)	24,939	(4,076 - 25,000)
Virgin Islands (UK)	0.53	(0.02 - 1)	18,682	(819 - 34,967)
Saint Barthelemy	1.00	(0.65 - 1)	9,990	(6,503 - 10,000)
Anguilla	0.47	(0.02 - 1)	7,957	(345 - 16,953)
Montserrat	0.62	(0.03 - 0.99)	3,086	(145 - 4,975)
Uruguay	0.00	(0 - 0.01)	0	(0 - 38,519)
