# Supplementary material for: Leveraging multiple data types to estimate the size of the Zika epidemic in the Americas
Source: PLoS Negl Trop Dis. 2020 Sep 28;14(9):e0008640. doi: 10.1371/journal.pntd.0008640 (PMC7544039; doi:10.1371/journal.pntd.0008640)
Supplement: S5 Table — (PDF) [file pntd.0008640.s009.pdf]

SI Table 5: Infection attacks rates and total infections based on parameter estimates from a subset of national models as indicated by the Models column.

Country	Models	IAR	95% CrI	Infections	95% CrI
Brazil	BOL, PER, COL	0.25	(0.19 - 0.31)	53,387,751	(40,817,578 - 64,920,837)
Mexico	GTM, BLZ	0.20	(0.15 - 0.25)	25,600,820	(19,230,749 - 32,693,013)
Colombia	PER, ECU, BRA	0.19	(0.15 - 0.23)	9,302,140	(7,132,424 - 11,358,764)
Ecuador	PER, BRA, COL	0.36	(0.21 - 0.51)	5,956,962	(3,523,784 - 8,483,764)
Guatemala	HND, BLZ, MEX, ELS	0.23	(0.16 - 0.29)	3,772,346	(2,715,238 - 4,820,425)
Venezuela	BRA, COL	0.10	(0.02 - 1)	3,303,034	(514,541 - 31,518,000)
Honduras	GTM, ELS	0.36	(0.22 - 0.49)	2,926,372	(1,823,954 - 4,043,373)
Peru	BOL, BRA, COL, ECU	0.08	(0.07 - 0.1)	2,673,080	(2,128,467 - 3,054,169)
Dominican Republic	PRI	0.25	(0.18 - 0.33)	2,657,461	(1,923,919 - 3,539,678)
Nicaragua	CRI, HND	0.33	(0.21 - 0.46)	2,061,578	(1,319,926 - 2,839,857)
Bolivia	PER, BRA	0.16	(0.07 - 0.29)	1,769,332	(794,430 - 3,171,504)
El Salvador	GTM, HND	0.28	(0.16 - 0.4)	1,717,496	(974,334 - 2,481,345)
Puerto Rico	PRI	0.32	(0.28 - 0.35)	1,164,658	(1,044,881 - 1,278,225)
Jamaica	PRI	0.20	(0.03 - 1)	552,682	(88,679 - 2,807,717)
Guadeloupe	PRI	0.99	(0.7 - 1)	466,652	(328,582 - 472,000)
Panama	CRI, COL	0.12	(0.06 - 0.22)	464,937	(238,548 - 886,299)
Costa Rica	PAN, NIC	0.09	(0.02 - 0.19)	448,112	(94,066 - 943,386)
Martinique	PRI	1.00	(0.97 - 1)	396,000	(384,344 - 396,000)
Haiti	PRI	0.02	(0 - 0.21)	212,650	(32,718 - 2,272,261)
Argentina	BOL, BRA	0.00	(0 - 0.08)	202,398	(8,725 - 3,308,168)
Suriname	BRA, COL	0.33	(0.06 - 1)	179,172	(31,414 - 547,947)
Paraguay	BOL, BRA	0.02	(0 - 0.37)	164,714	(6,697 - 2,491,821)
French Guiana	BRA, COL	0.59	(0.41 - 1)	161,718	(114,220 - 275,779)
Trinidad & Tobago	PRI	0.12	(0.01 - 1)	157,540	(17,621 - 1,366,866)
Curacao	PRI	0.65	(0.46 - 1)	96,626	(67,946 - 148,919)
Belize	GTM, MEX	0.23	(0.07 - 0.49)	85,426	(24,825 - 181,213)
Dominica	PRI	0.99	(0.18 - 1)	73,590	(13,535 - 74,000)
Saint Lucia	PRI	0.37	(0.06 - 1)	60,549	(9,634 - 164,935)
Barbados	PRI	0.20	(0.03 - 1)	59,814	(9,552 - 291,662)

Cuba	PRI	0.00	(0 - 0.17)	42,167	(4,514 - 1,928,151)
St Kitts & Nevis	PRI	0.76	(0.12 - 1)	40,204	(6,505 - 52,992)
St Vincent & the Grenadines	PRI	0.39	(0.06 - 1)	40,197	(6,496 - 101,949)
Bahamas	PRI	0.10	(0.02 - 0.95)	38,796	(6,100 - 373,545)
Antigua and Barbuda	PRI	0.41	(0.07 - 1)	38,606	(6,217 - 93,965)
Saint Martin	PRI	1.00	(0.99 - 1)	35,977	(35,668 - 36,000)
Virgin Islands (US)	PRI	0.31	(0.22 - 0.55)	32,112	(22,824 - 56,171)
Aruba	PRI	0.25	(0.17 - 0.44)	28,060	(19,698 - 50,508)
Guyana	BRA, COL	0.03	(0 - 0.27)	20,807	(1,803 - 209,236)
Saint Barthelemy	PRI	1.00	(0.99 - 1)	9,983	(9,873 - 10,000)
Bonaire, St Eustatius & Saba	PRI	0.39	(0.28 - 0.73)	9,857	(6,878 - 18,312)
Grenada	PRI	0.06	(0.04 - 0.12)	6,740	(4,737 - 12,759)
Sint Maarten (Dutch part)	PRI	0.14	(0.1 - 0.27)	5,986	(4,077 - 11,380)
Cayman Islands	PRI	0.07	(0.05 - 0.13)	3,915	(2,719 - 7,321)
Anguilla	PRI	0.22	(0.03 - 0.99)	3,752	(560 - 16,790)
Turks & Caicos	PRI	0.07	(0.04 - 0.12)	3,380	(2,316 - 6,323)
Virgin Islands (UK)	PRI	0.05	(0.04 - 0.1)	1,891	(1,247 - 3,523)
Montserrat	PRI	0.07	(0.04 - 0.14)	352	(194 - 719)
Uruguay	BRA, BOL	0.00	(0 - 0)	0	(0 - 4,092)
