# Supplementary material for: Leveraging multiple data types to estimate the size of the Zika epidemic in the Americas
Source: PLoS Negl Trop Dis. 2020 Sep 28;14(9):e0008640. doi: 10.1371/journal.pntd.0008640 (PMC7544039; doi:10.1371/journal.pntd.0008640)
Supplement: S6 Table — (PDF) [file pntd.0008640.s010.pdf]

SI Table 6: Comparison of model-specific IAR estimates for each modeled territory versus projections of IAR from combined model estimates under the two different projection methods.

			Default		Alternative	
Country	Modeled IAR	95% CrI	Projected IAR	95% CrI	Projected IAR	95% CrI
Belize	0.23	(0.07 - 0.49)	0.85	(0.04 - 1)	0.99	(0.28 - 1)
Bolivia	0.16	(0.07 - 0.29)	0.05	(0 - 1)	0.04	(0 - 1)
Brazil	0.25	(0.19 - 0.31)	0.27	(0.01 - 1)	0.50	(0.04 - 1)
Colombia	0.19	(0.15 - 0.23)	0.30	(0.02 - 1)	0.43	(0.02 - 1)
Costa Rica	0.09	(0.02 - 0.19)	0.27	(0.01 - 1)	0.51	(0.03 - 1)
Dominican Republic	0.25	(0.18 - 0.33)	0.06	(0 - 1)	0.03	(0 - 1)
Ecuador	0.36	(0.21 - 0.51)	0.06	(0 - 1)	0.09	(0 - 0.69)
El Salvador	0.28	(0.16 - 0.4)	0.18	(0.01 - 1)	0.08	(0 - 1)
Guatemala	0.23	(0.16 - 0.29)	0.04	(0 - 1)	0.86	(0.16 - 1)
Honduras	0.36	(0.22 - 0.49)	0.37	(0.02 - 1)	0.21	(0 - 0.62)
Mexico	0.20	(0.15 - 0.25)	0.07	(0 - 1)	0.06	(0 - 0.54)
Nicaragua	0.33	(0.21 - 0.46)	0.37	(0.01 - 1)	0.10	(0.02 - 0.73)
Panama	0.12	(0.06 - 0.22)	0.24	(0.01 - 1)	0.05	(0 - 1)
Peru	0.08	(0.07 - 0.1)	0.04	(0 - 1)	0.04	(0.01 - 0.39)
Puerto Rico	0.32	(0.29 - 0.35)	1.00	(0.15 - 1)	0.36	(0.25 - 0.56)
