# Supplementary material for: Leveraging multiple data types to estimate the size of the Zika epidemic in the Americas
Source: PLoS Negl Trop Dis. 2020 Sep 28;14(9):e0008640. doi: 10.1371/journal.pntd.0008640 (PMC7544039; doi:10.1371/journal.pntd.0008640)
Supplement: S7 Table — (PDF) [file pntd.0008640.s011.pdf]

SI Table 7: Zika seroprevalence studies.

Country	ISO A1	Location	Infected	Tested	IAR (95% CI)	Sampling Period	Source
Bolivia	Beni	Beni	41	105	0.39 (0.30-0.49)	12/2016	[52]
Bolivia	Santa Cruz	Santa Cruz	43	200	0.22 (0.16-0.28)	3/2017 - 4/2017	[52]
Bolivia	Tarija	Tarija	1	196	0.01 (0.00-0.02)	3/2017 - 4/2017	[52]
Bolivia	La Paz	La Paz	0	162	0.00 (0.00-0.02)	3/2017 - 4/2017	[52]
Bolivia	Cochabamba	Cochabamba	0	152	0.00 (0.00-0.02)	3/2017 - 4/2017	[52]
Brazil	Bahia	Salvador	401	633	0.63 (0.60-0.67)	1/2016 - 5/2016	[25]
Brazil	Bahia	Salvador	108	160	0.68 (0.60-0.74)	5/2015 - 10/2016	[26]
Brazil	Bahia	Salvador	1061	1453	0.73 (0.70-0.76)	10/2015	[17]
Brazil	Pernambuco	Recife	39	61	0.64 (0.53-0.75)	1/15/2016 - 5/2/2016	[27]
Brazil	Sao Paulo	Santos	53	740	0.07 (0.05-0.09)	6/2016 - 2/2017	[98]
Brazil	Rio Grande do Sol	Santa Maria	1	182	0.01 (0.00-0.02)	12/5/2016 - 1/6/2017	[99]
Ecuador	Guayas	Guayaquil	14	28	0.50 (0.32-0.68)	6/1/2016 - 7/31/2016	[46]
Nicaragua	Managua	Managua	983	2147	0.46 (0.44-0.48)	2/2017 - 7/2017	[24]
Puerto Rico	Bayamon	Bayamon	75	216	0.35 (0.28-0.41)	9/16/2016 - 10/27/2016	[53]
Puerto Rico	Carolina	Carolina	10	42	0.24 (0.12-0.38)	9/16/2016 - 10/27/2016	[53]
Puerto Rico	Guayanabo	Guayanabo	3	14	0.21 (0.00-0.43)	9/16/2016 - 10/27/2016	[53]
Puerto Rico	Toa Alta	Toa Alta	21	70	0.30 (0.20-0.41)	9/16/2016 - 10/27/2016	[53]
Puerto Rico	Toa Baja	Toa Baja	5	25	0.20 (0.04-0.36)	9/16/2016 - 10/27/2016	[53]
