# Supplementary figures and images for: Leveraging multiple data types to estimate the size of the Zika epidemic in the Americas
Source: PLoS Negl Trop Dis. 2020 Sep 28;14(9):e0008640. doi: 10.1371/journal.pntd.0008640 (PMC7544039; doi:10.1371/journal.pntd.0008640)

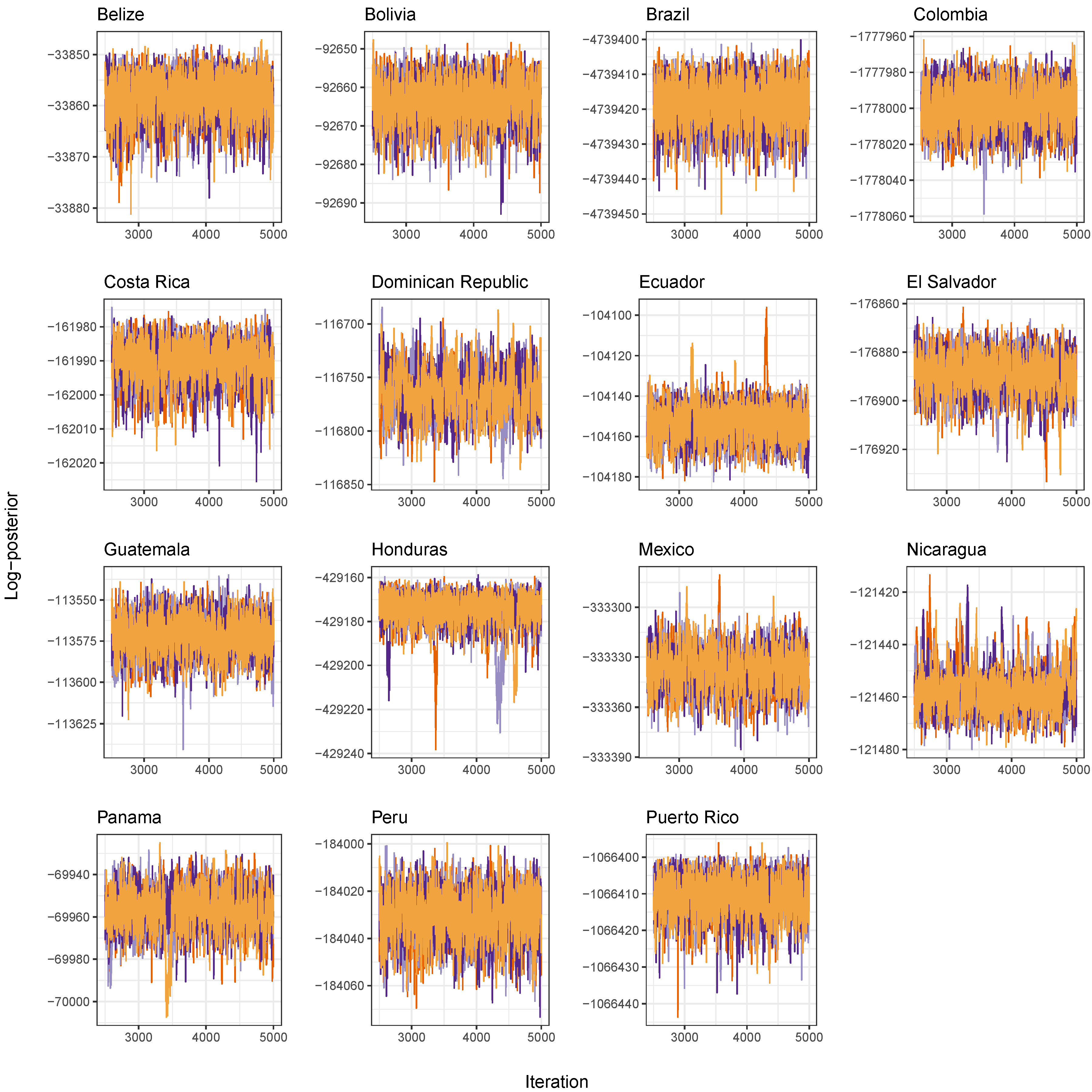

Supplement: S1 Fig — Colors represent the four separate chains. (TIF) [file pntd.0008640.s012.tif]

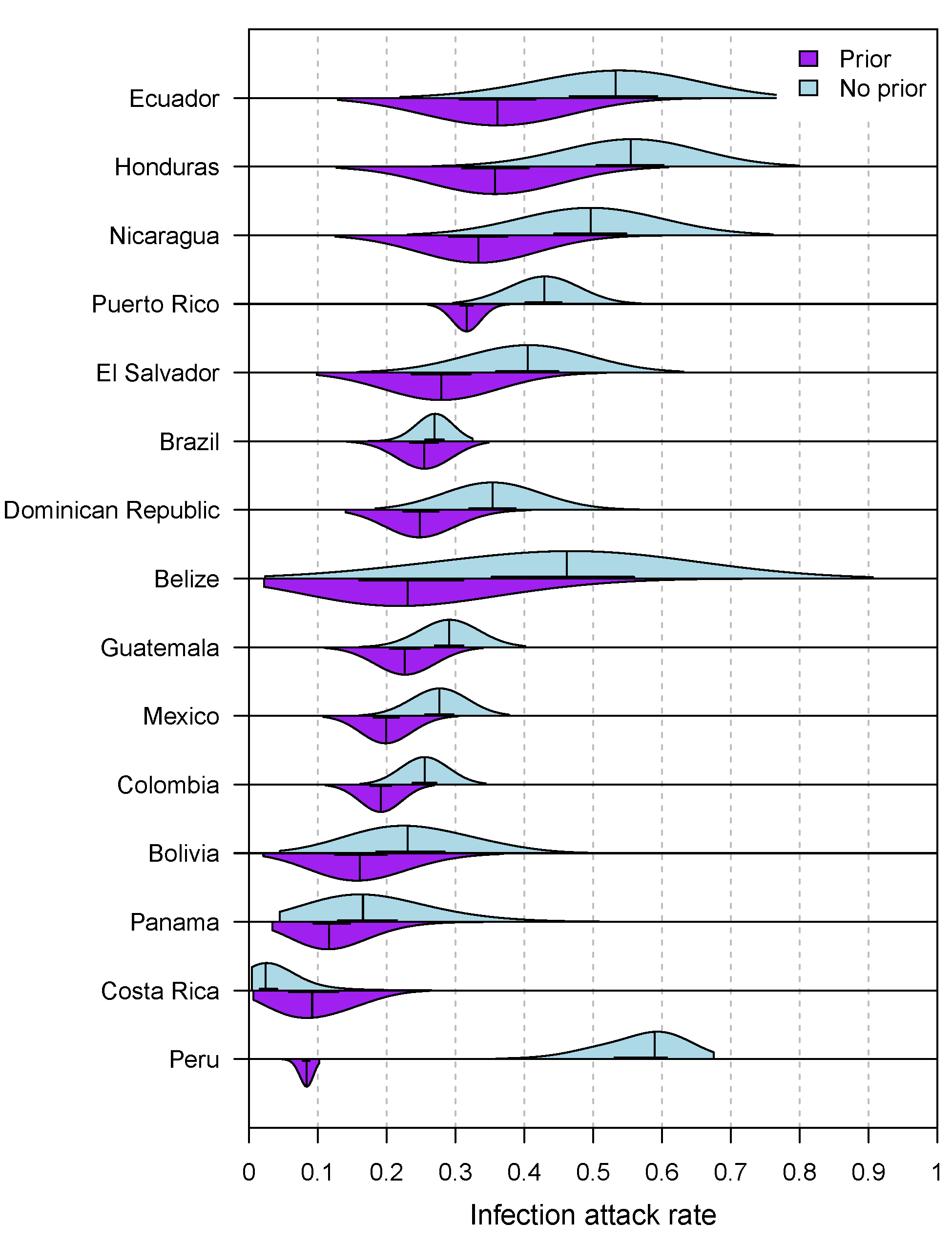

Supplement: S2 Fig — (TIF) [file pntd.0008640.s013.tif]

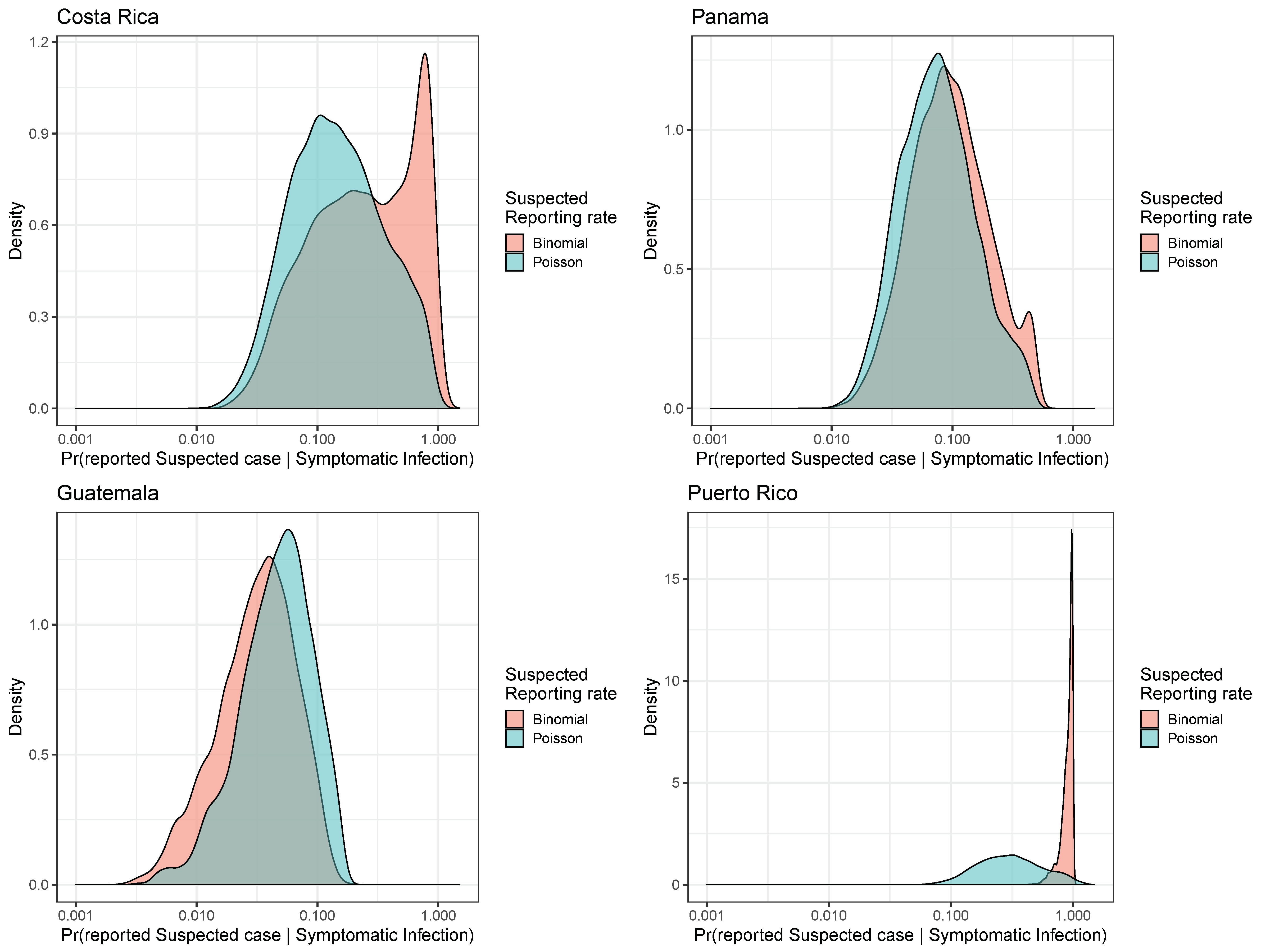

Supplement: S3 Fig — (TIF) [file pntd.0008640.s014.tif]

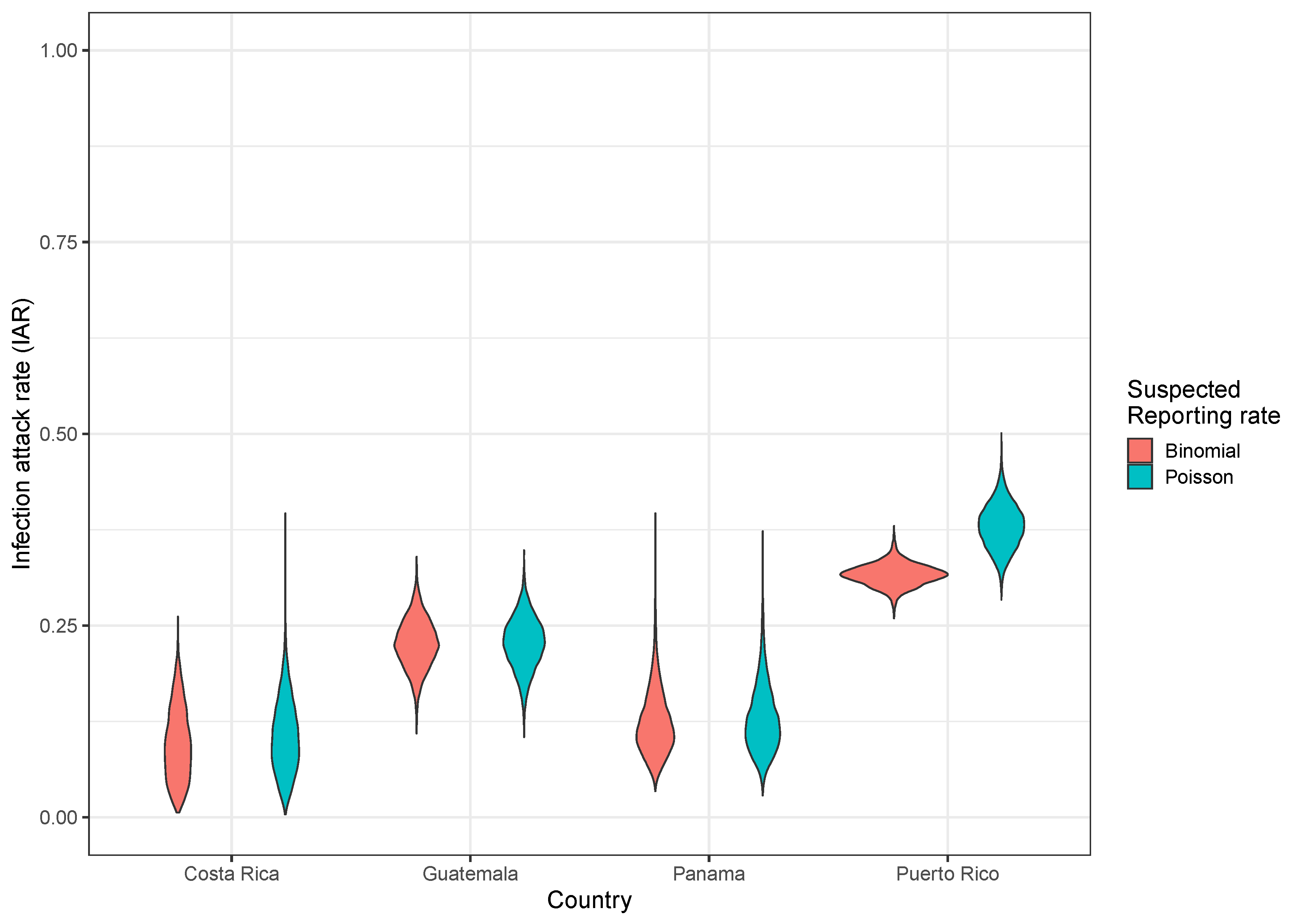

Supplement: S4 Fig — (TIF) [file pntd.0008640.s015.tif]

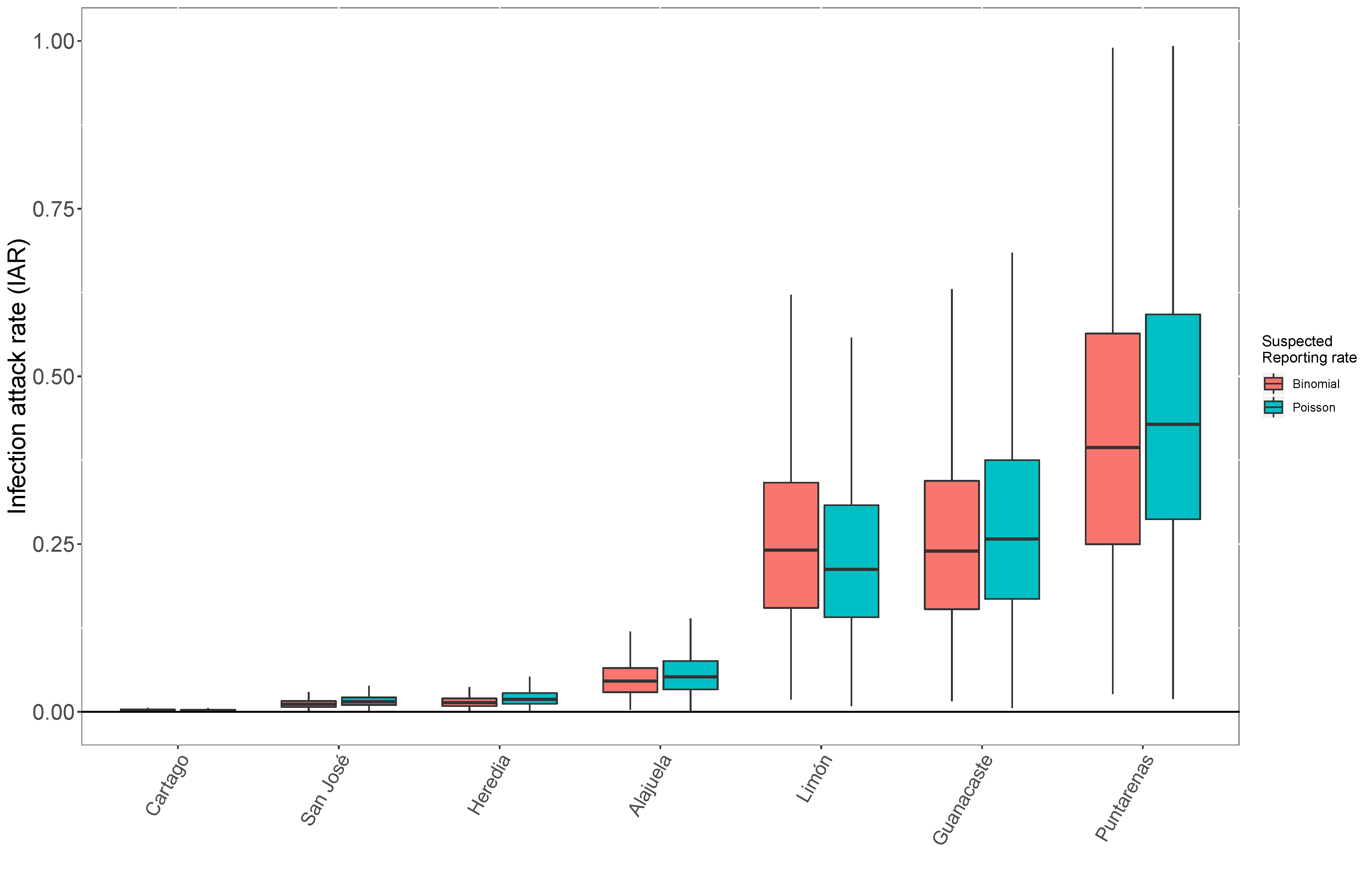

Supplement: S5 Fig — (TIF) [file pntd.0008640.s016.tif]

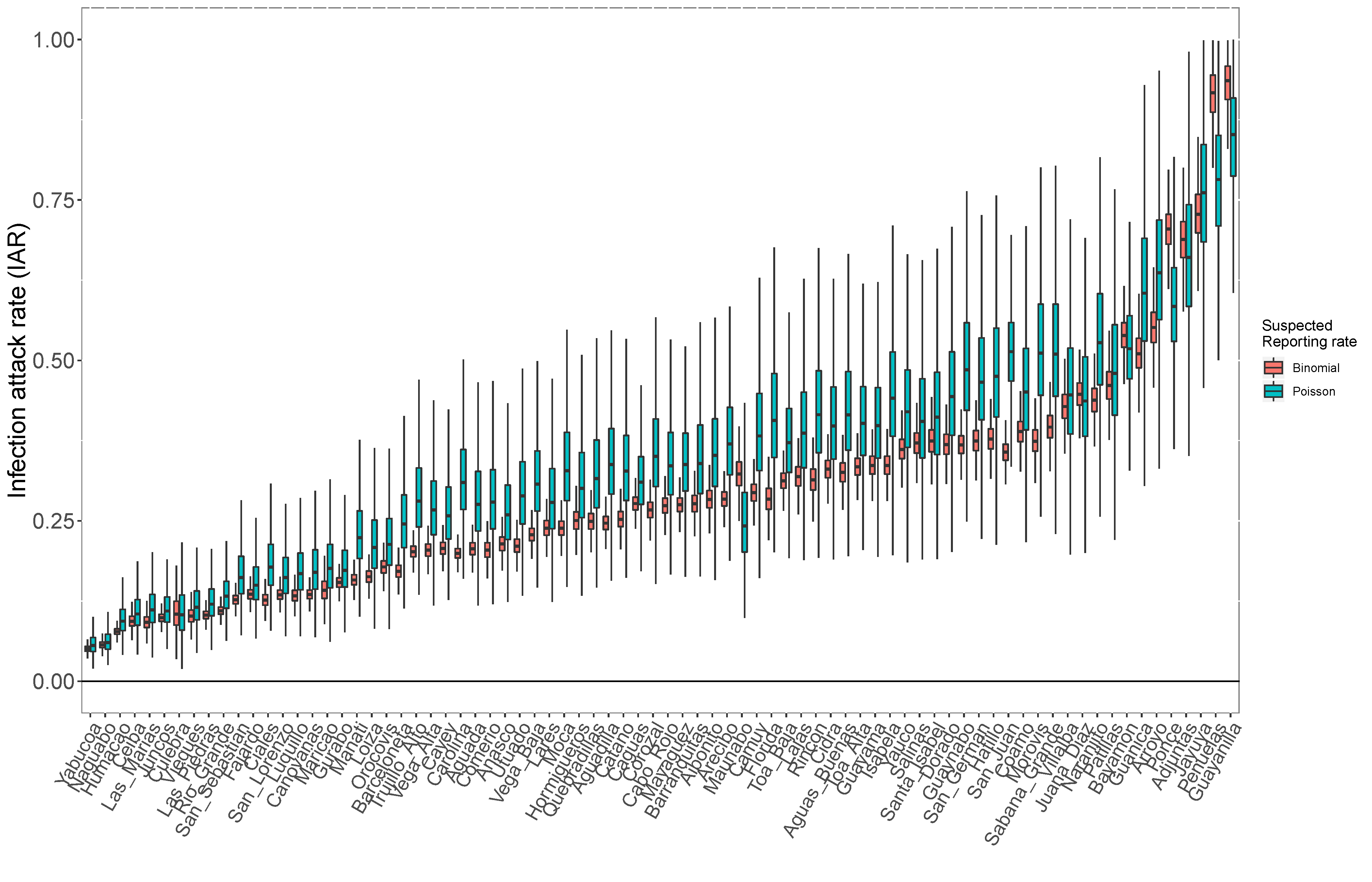

Supplement: S6 Fig — (TIF) [file pntd.0008640.s017.tif]

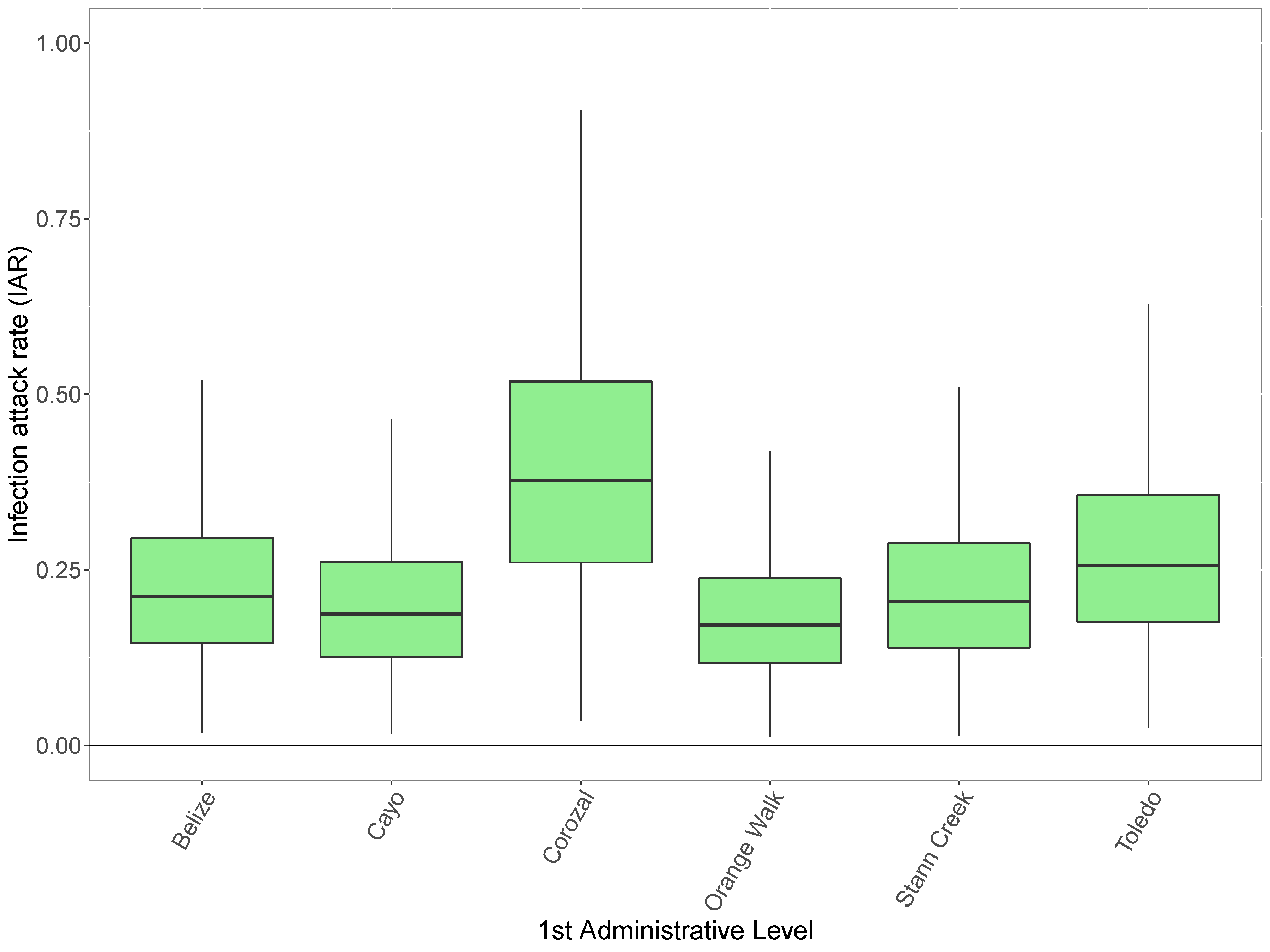

Supplement: S7 Fig — (TIF) [file pntd.0008640.s018.tif]

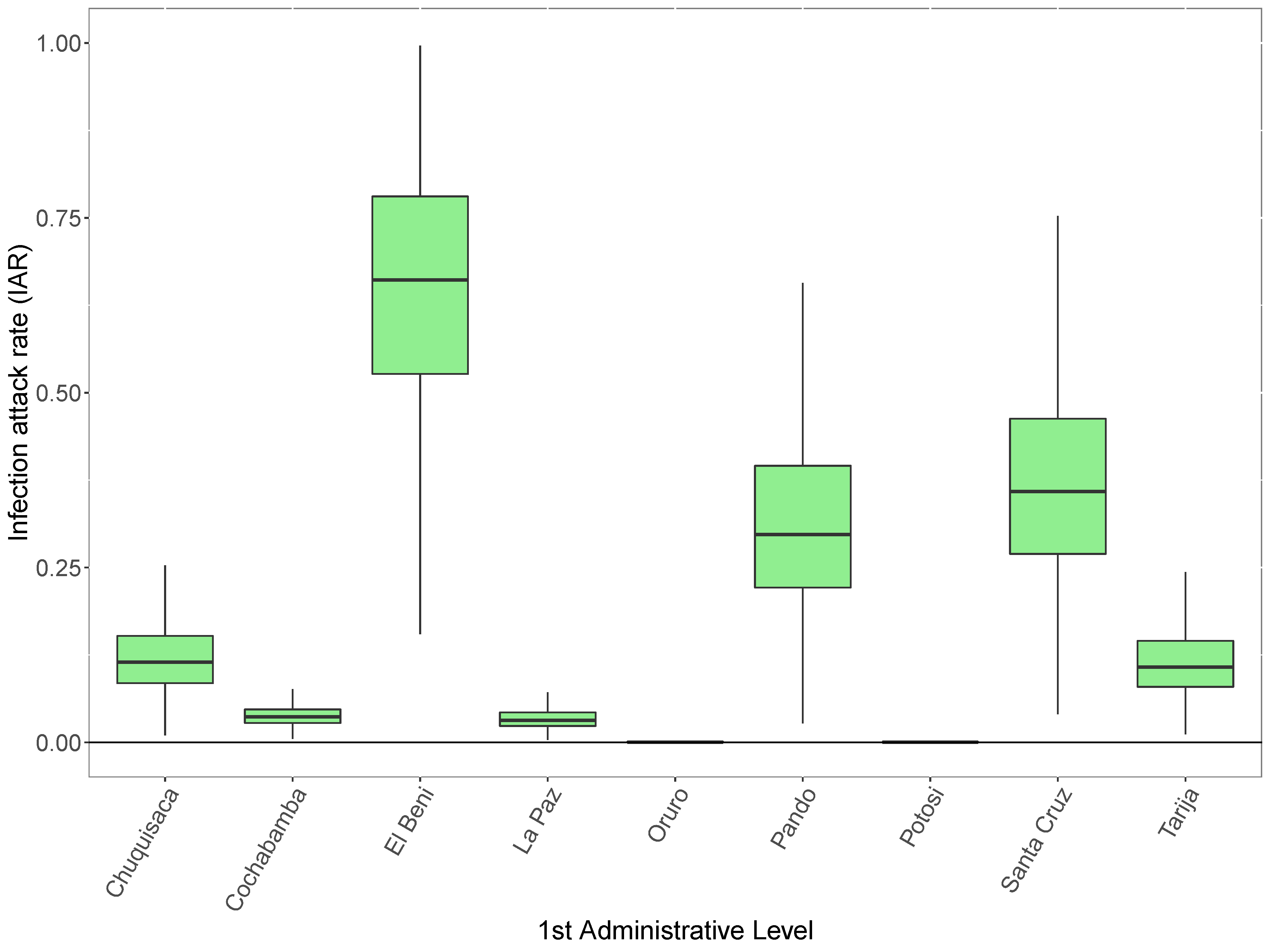

Supplement: S8 Fig — (TIF) [file pntd.0008640.s019.tif]

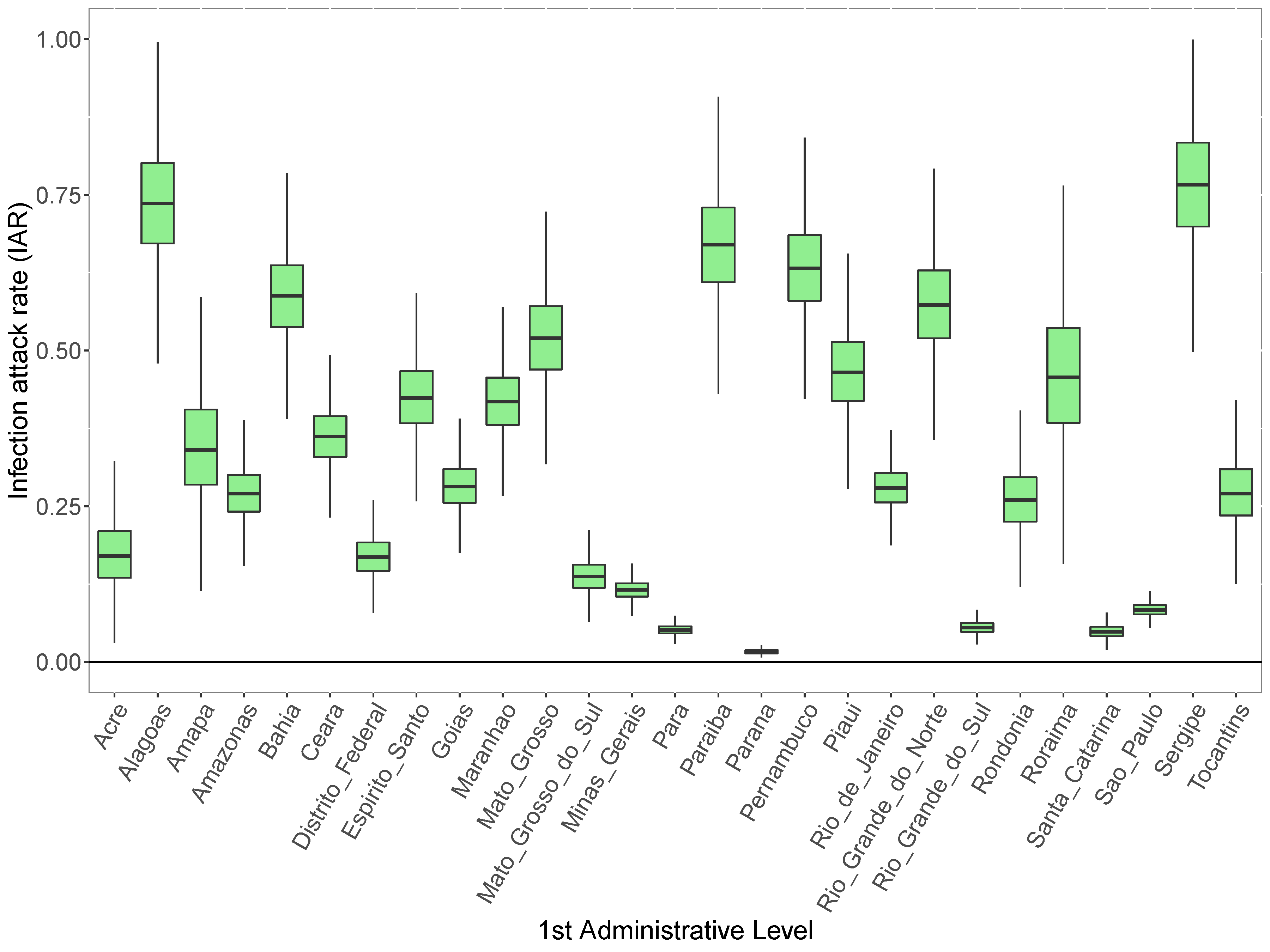

Supplement: S9 Fig — (TIF) [file pntd.0008640.s020.tif]

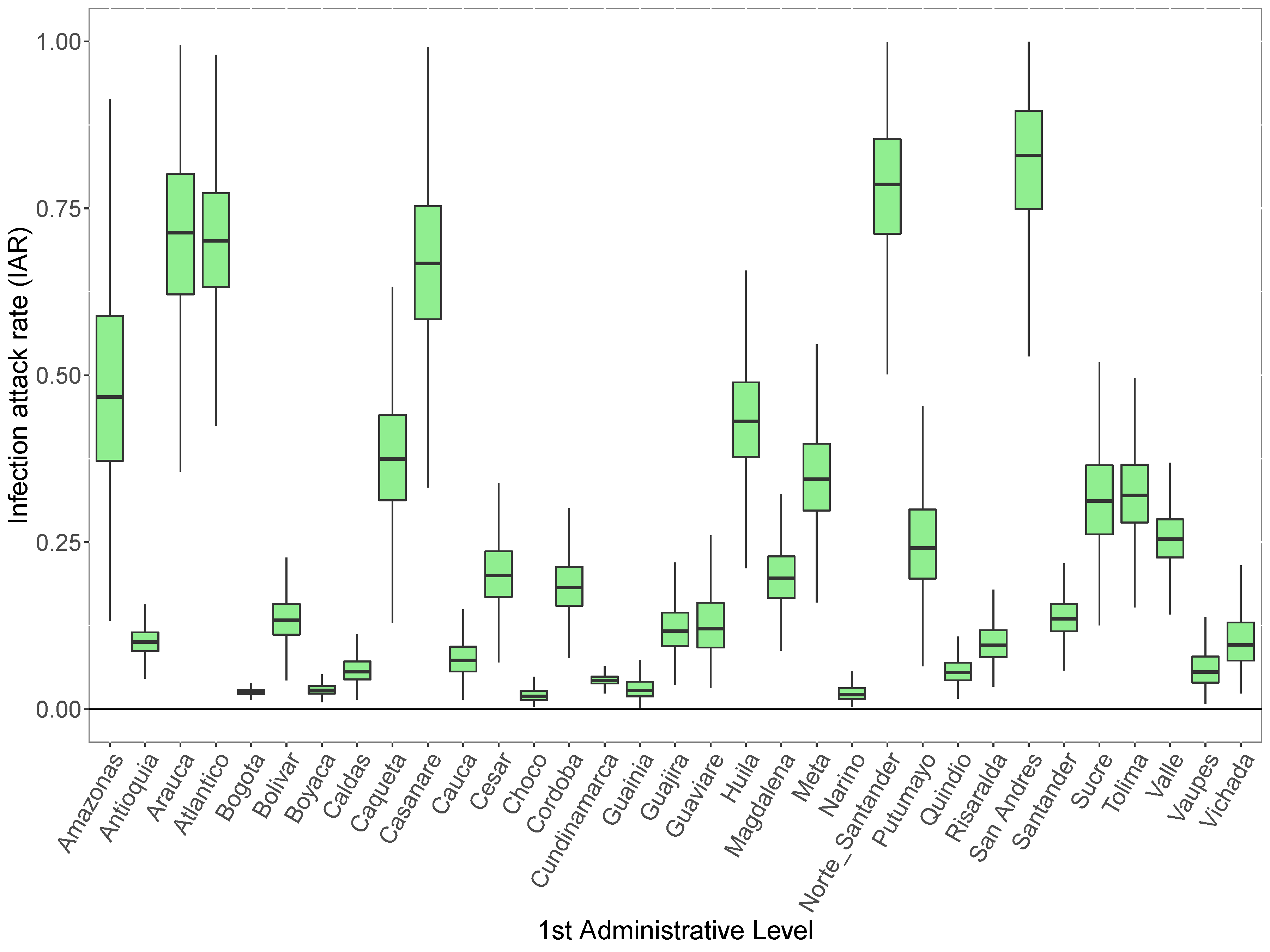

Supplement: S10 Fig — (TIF) [file pntd.0008640.s021.tif]

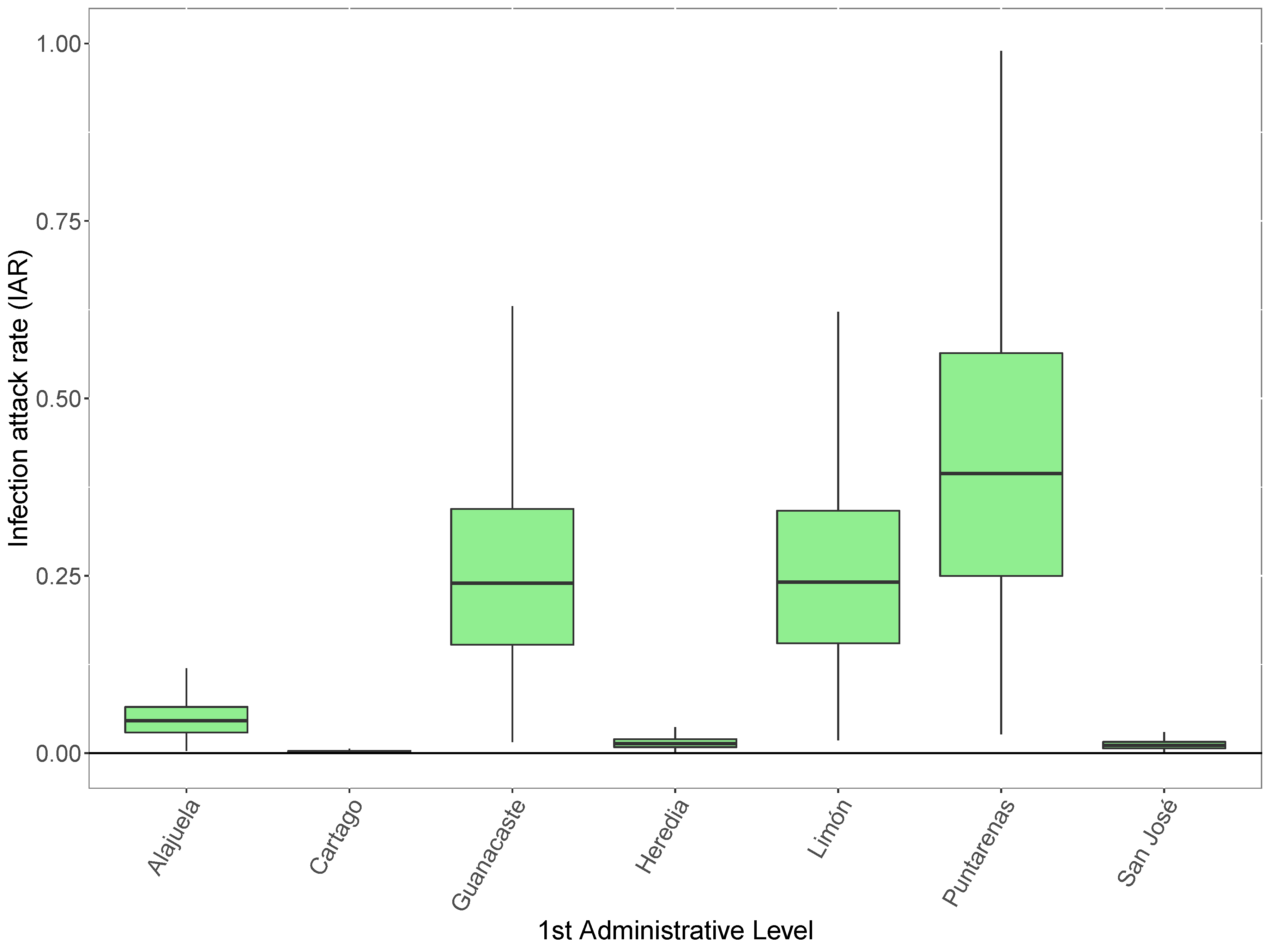

Supplement: S11 Fig — (TIF) [file pntd.0008640.s022.tif]

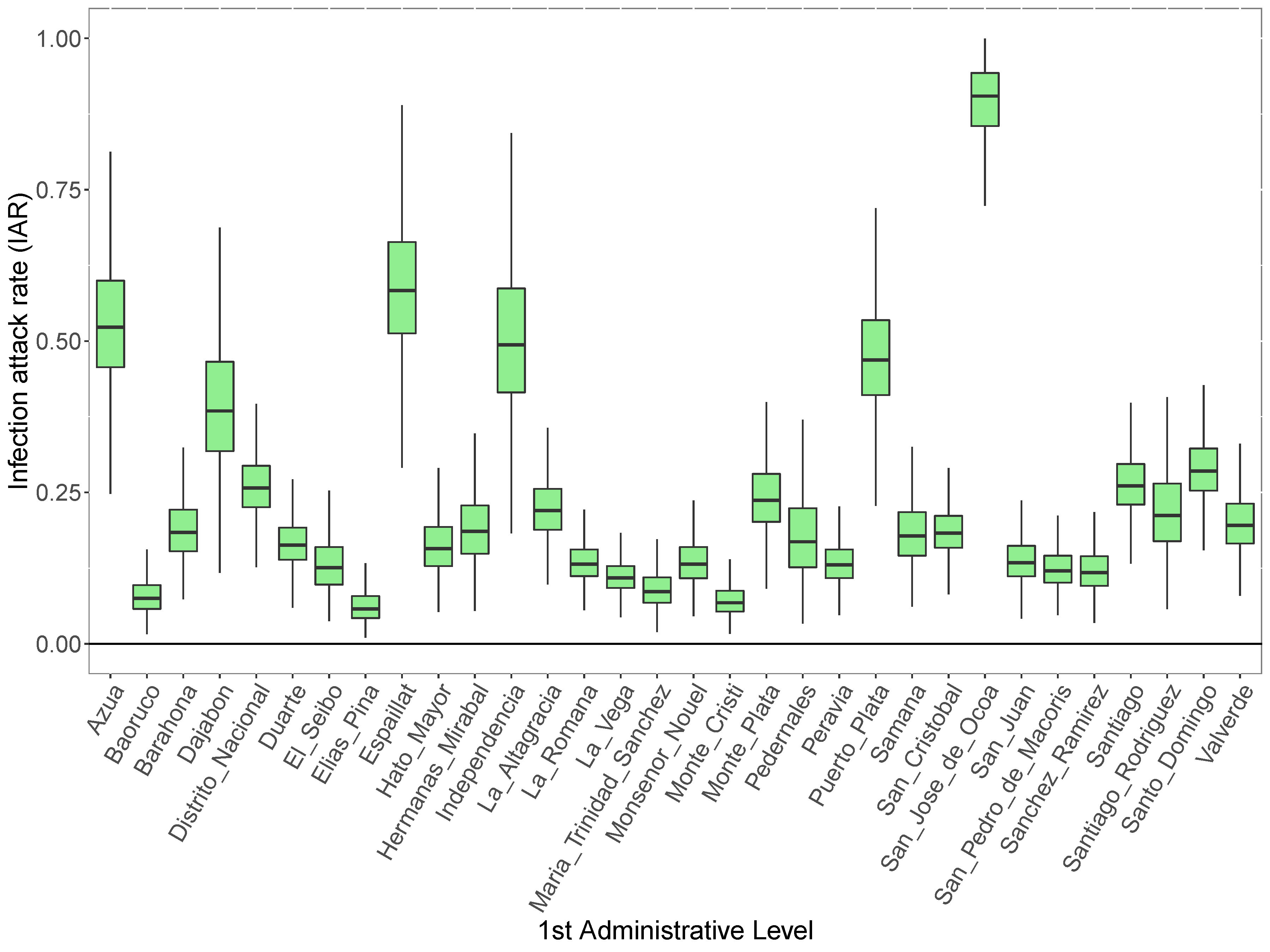

Supplement: S12 Fig — (TIF) [file pntd.0008640.s023.tif]

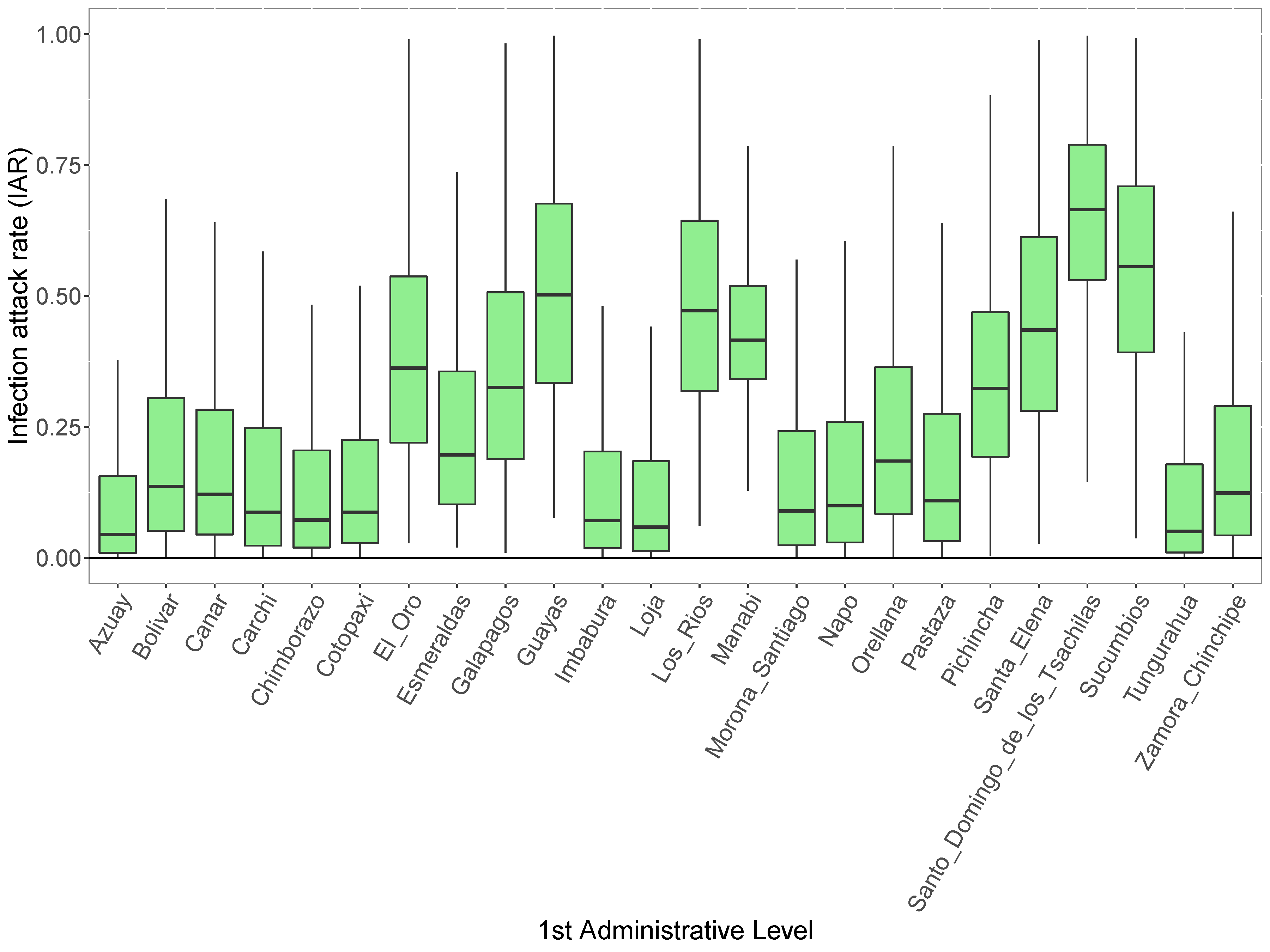

Supplement: S13 Fig — (TIF) [file pntd.0008640.s024.tif]

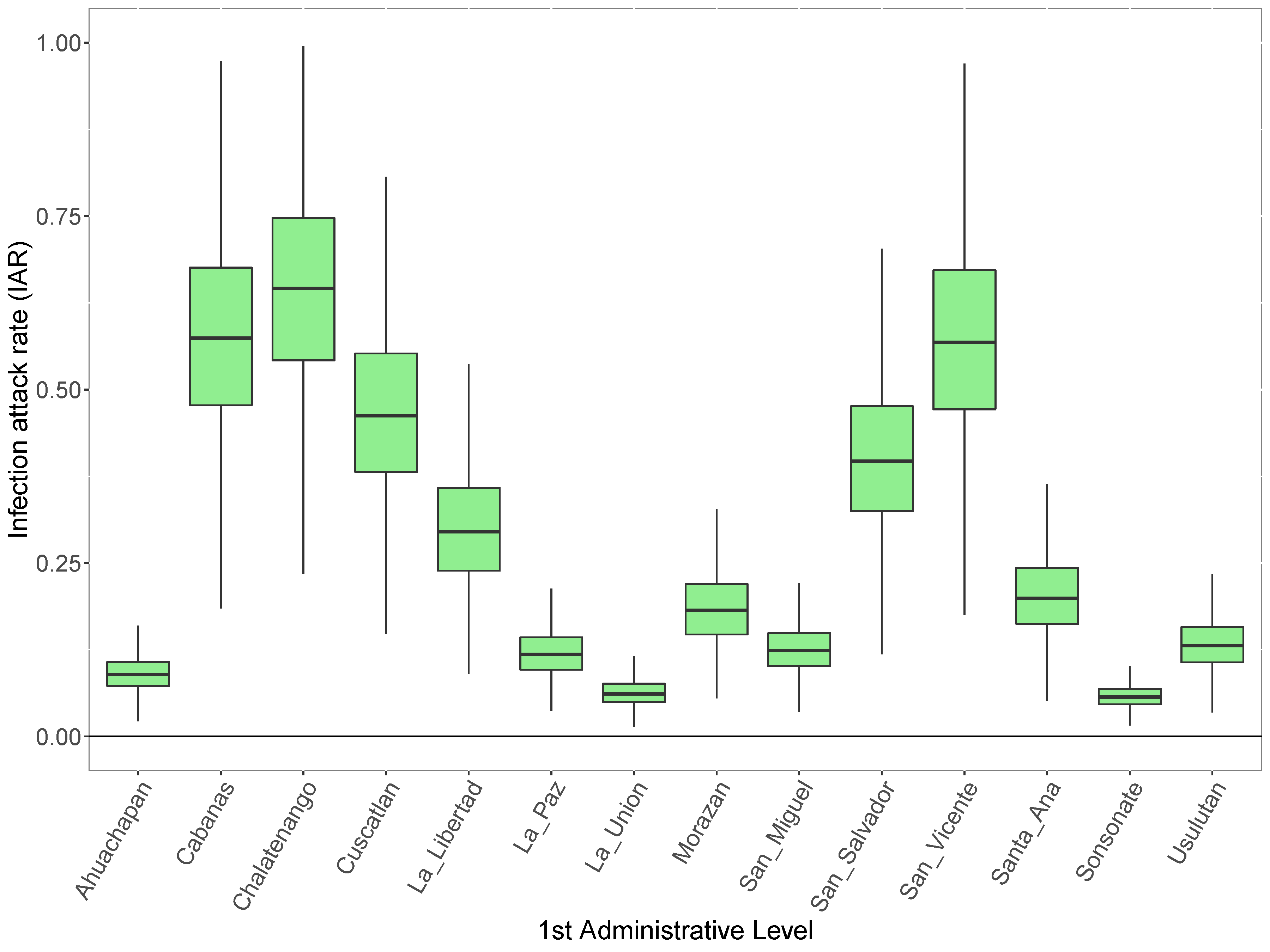

Supplement: S14 Fig — (TIF) [file pntd.0008640.s025.tif]

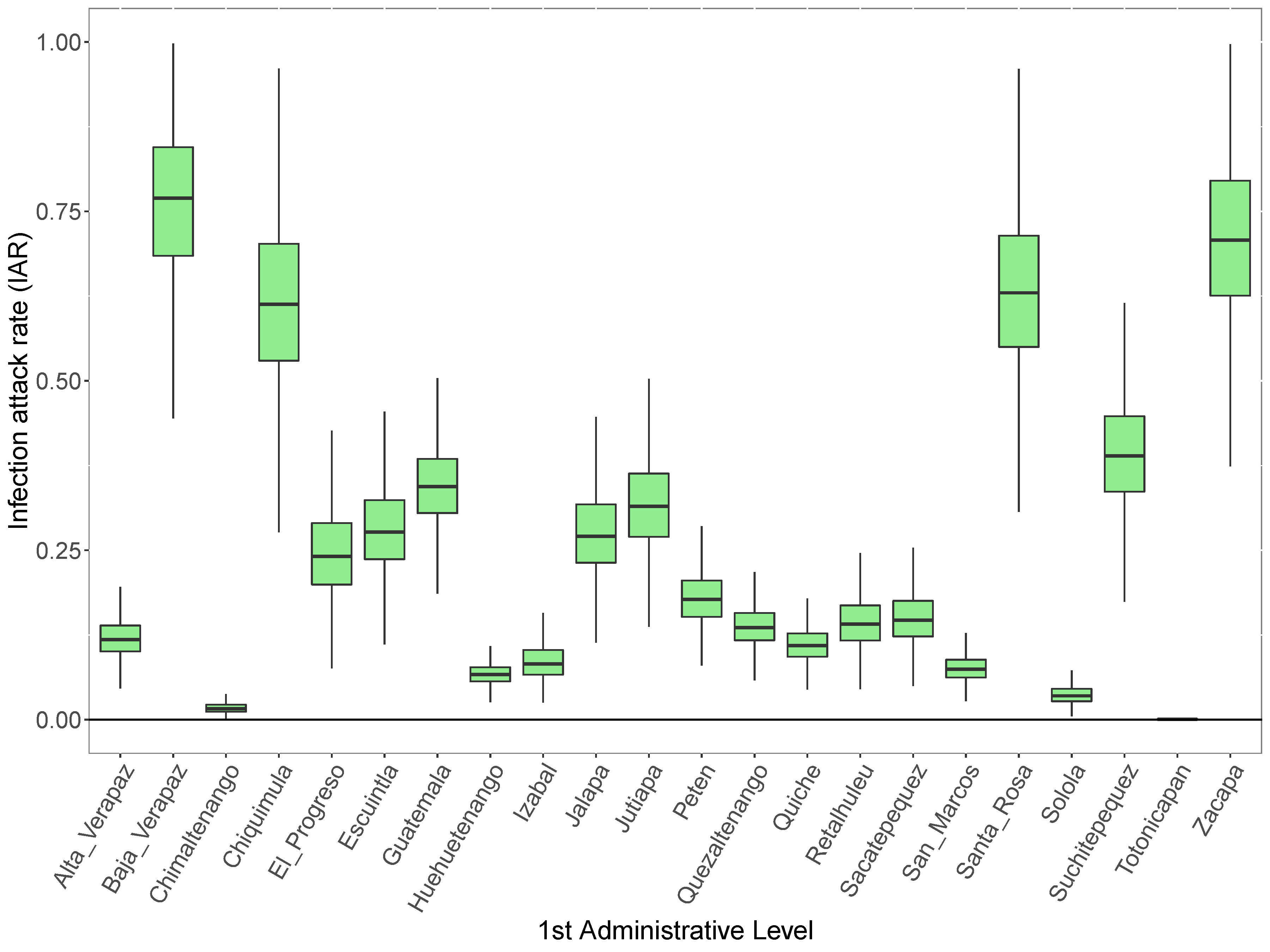

Supplement: S15 Fig — (TIF) [file pntd.0008640.s026.tif]

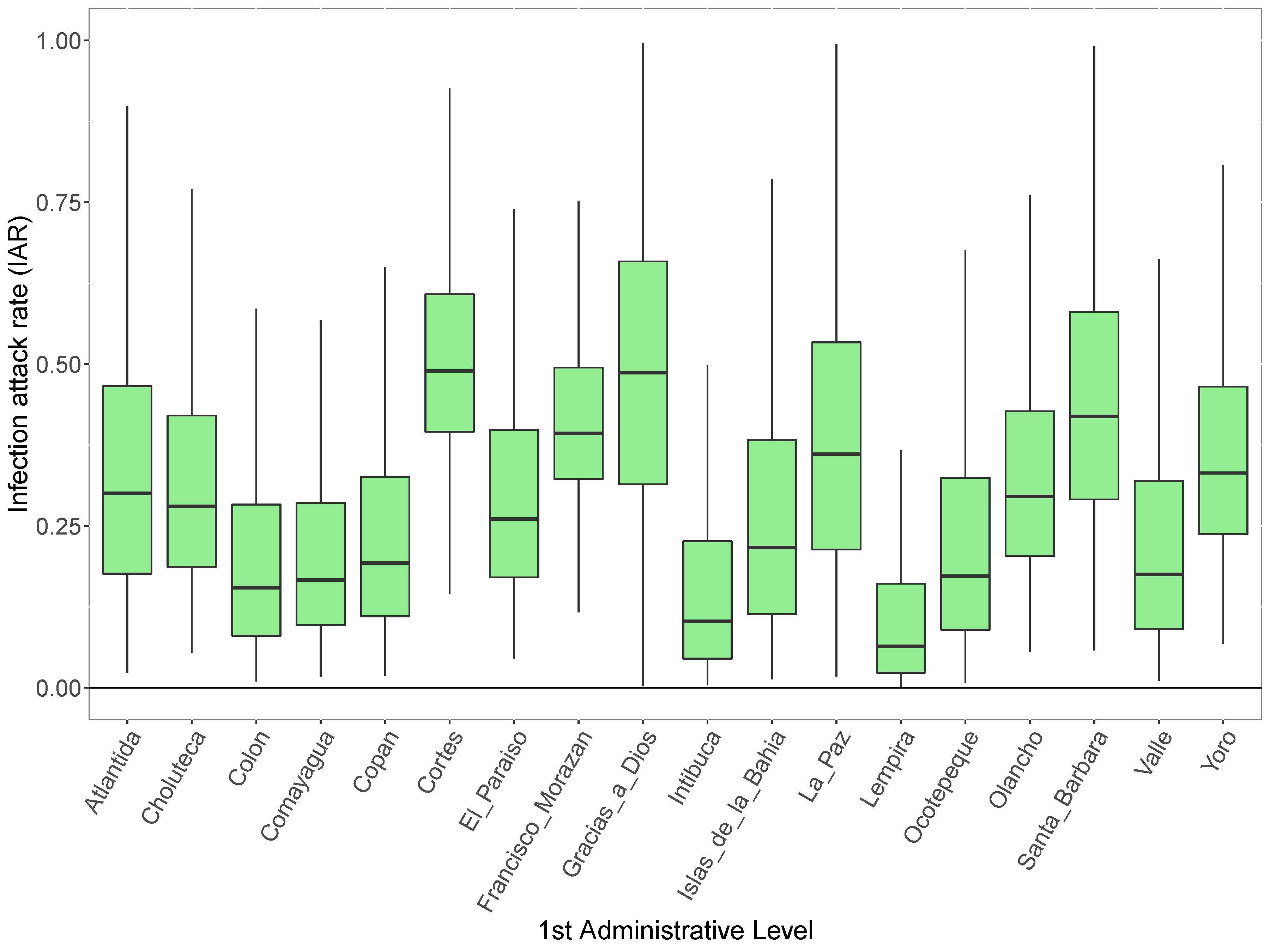

Supplement: S16 Fig — (TIF) [file pntd.0008640.s027.tif]

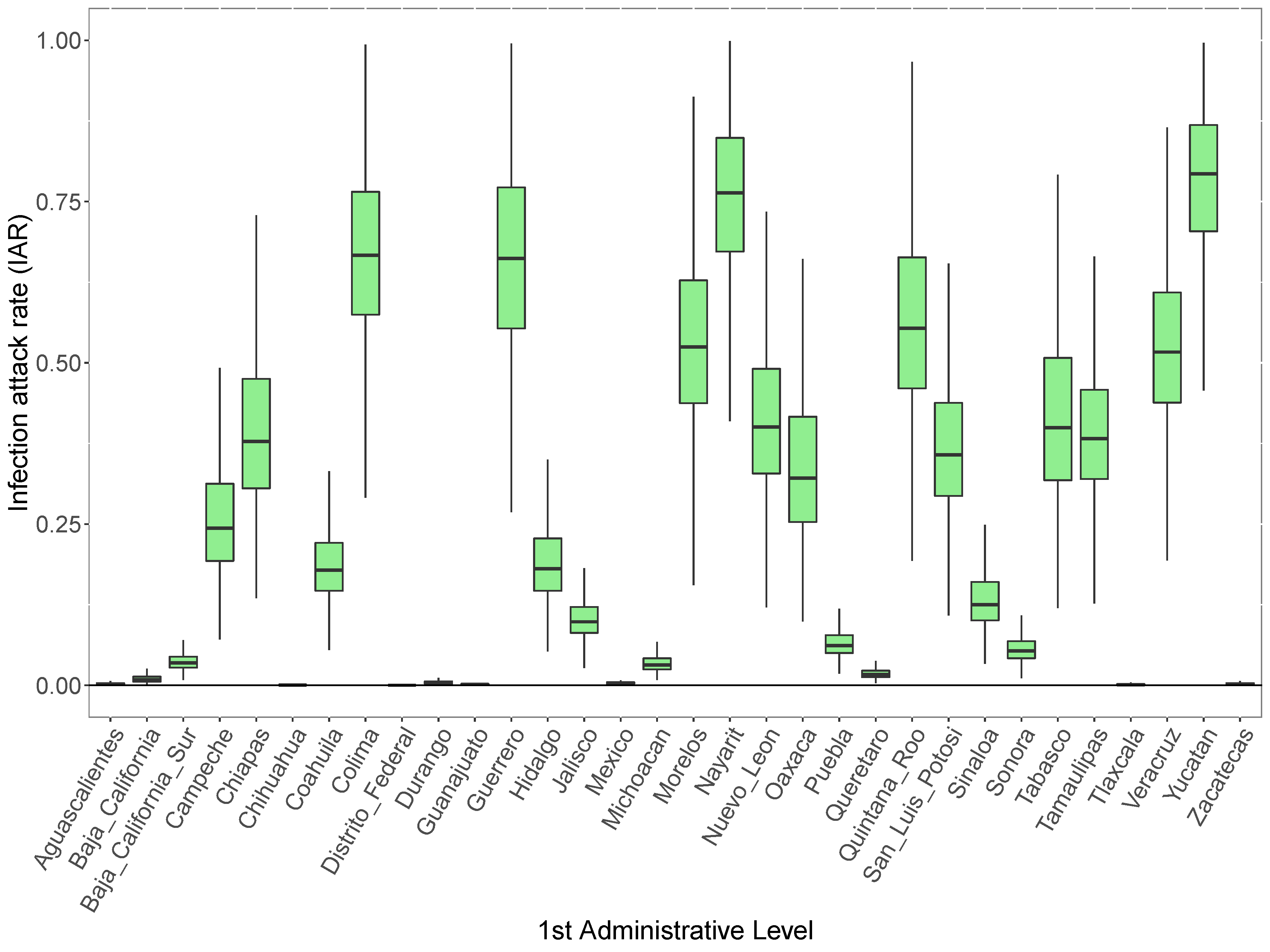

Supplement: S17 Fig — (TIF) [file pntd.0008640.s028.tif]

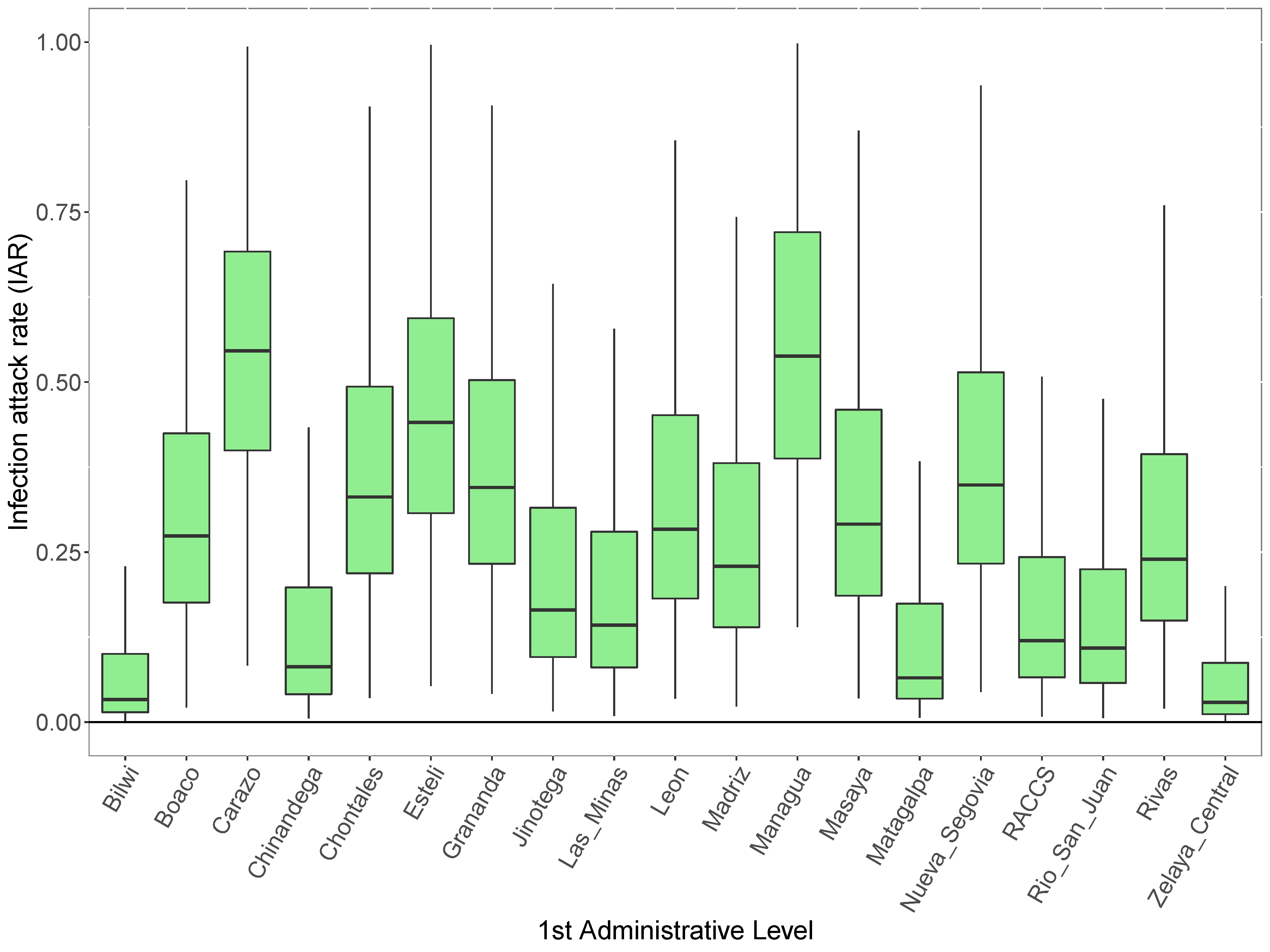

Supplement: S18 Fig — (TIF) [file pntd.0008640.s029.tif]

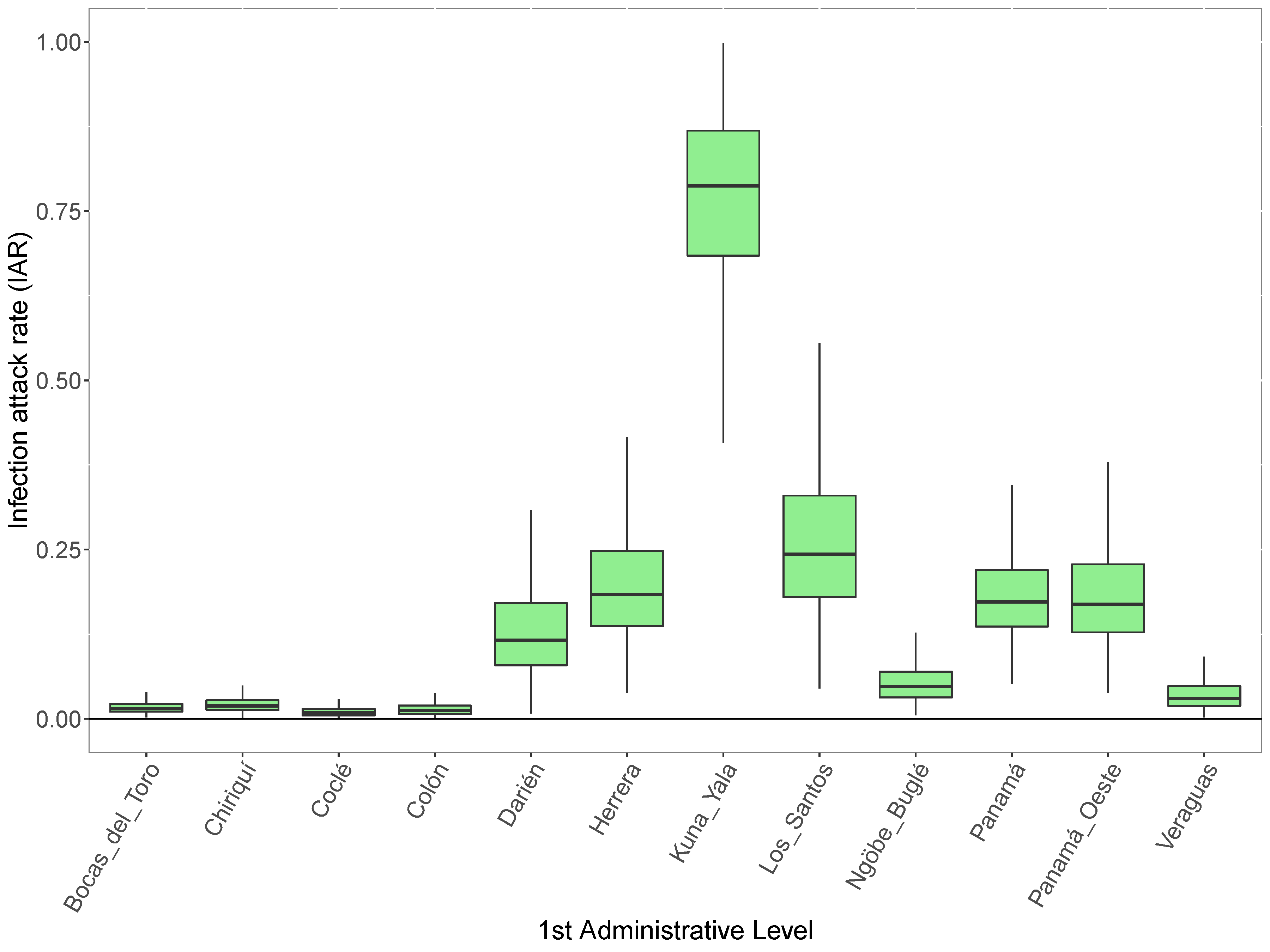

Supplement: S19 Fig — (TIF) [file pntd.0008640.s030.tif]

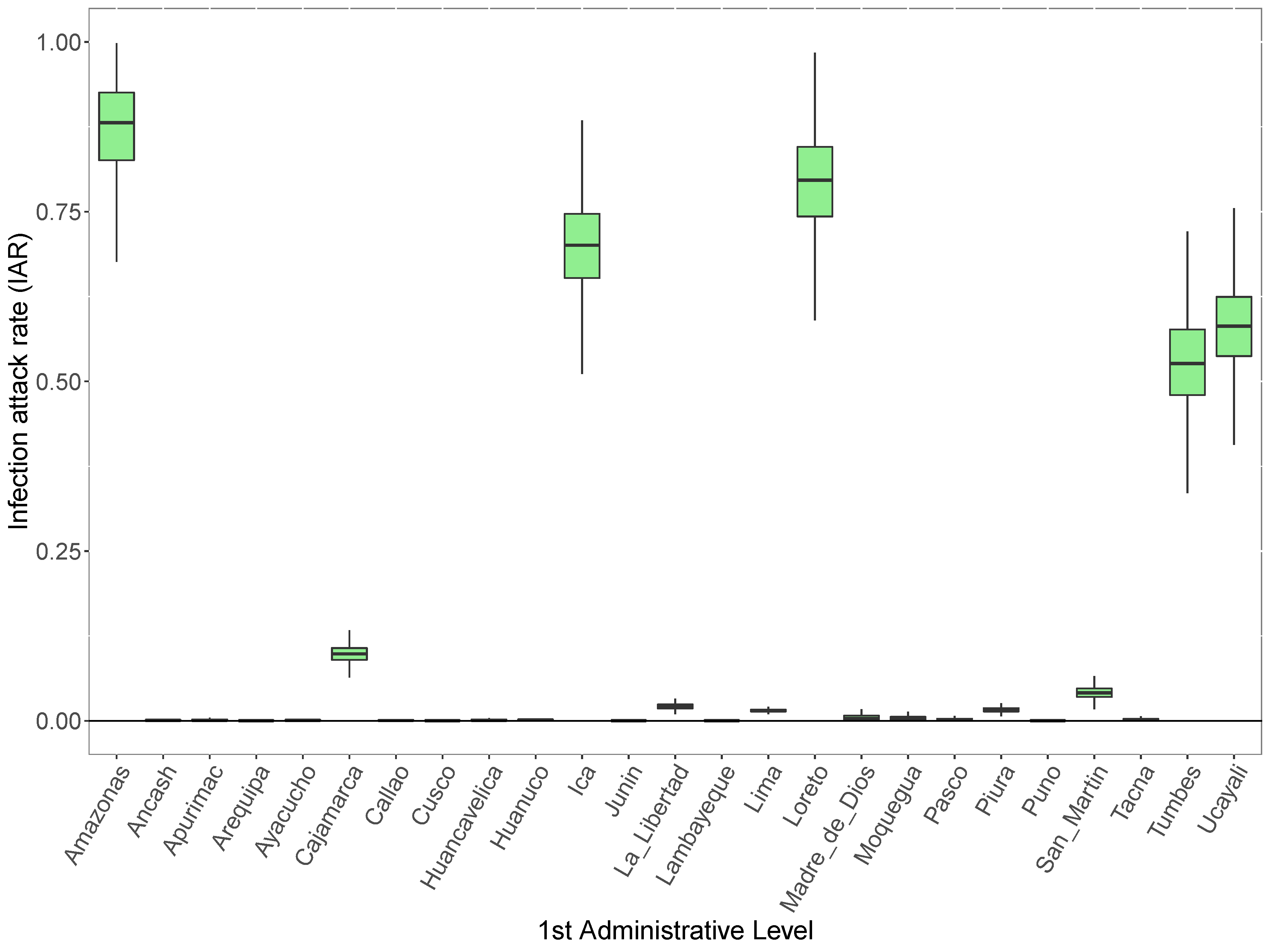

Supplement: S20 Fig — (TIF) [file pntd.0008640.s031.tif]

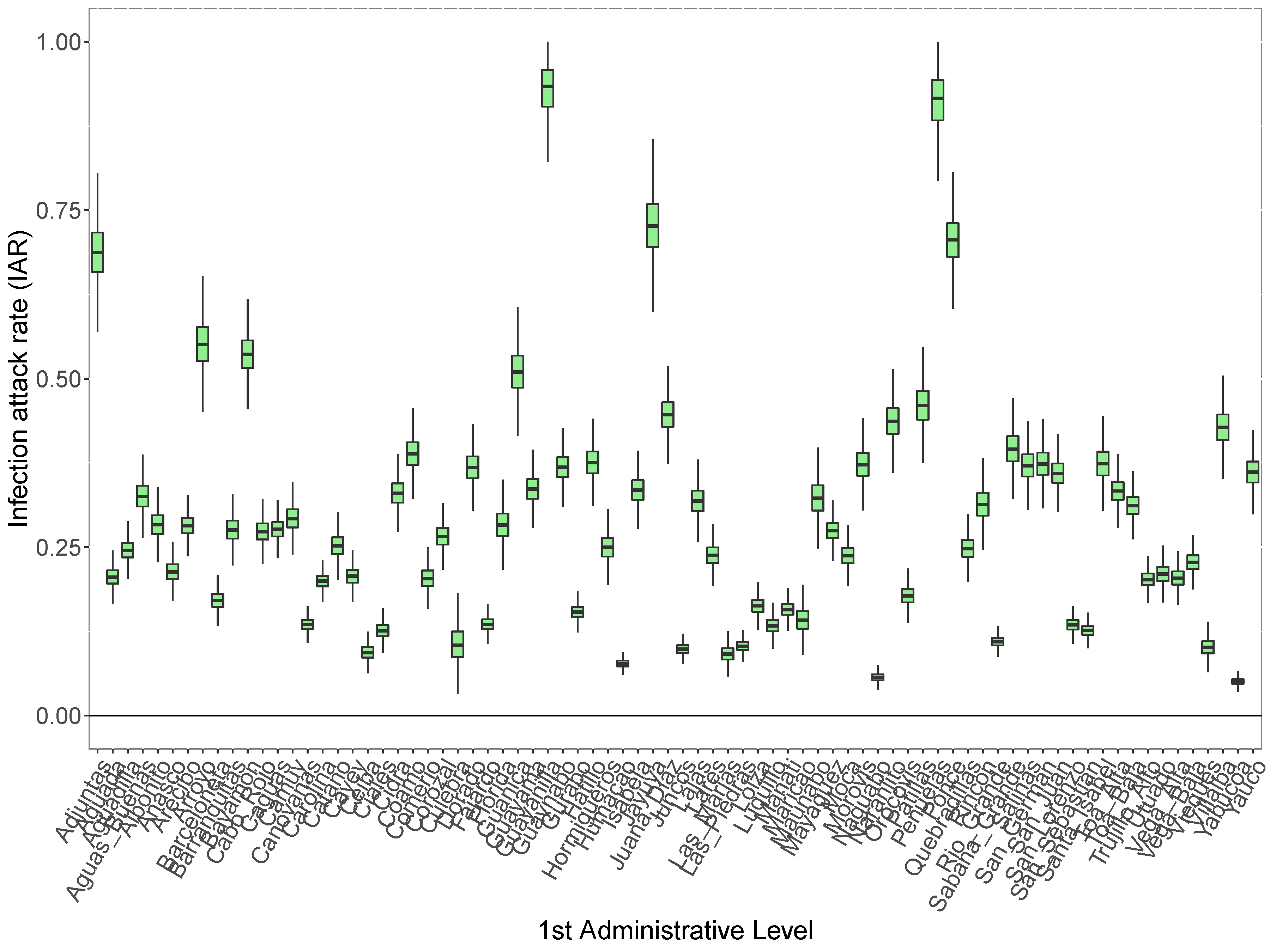

Supplement: S21 Fig — (TIF) [file pntd.0008640.s032.tif]

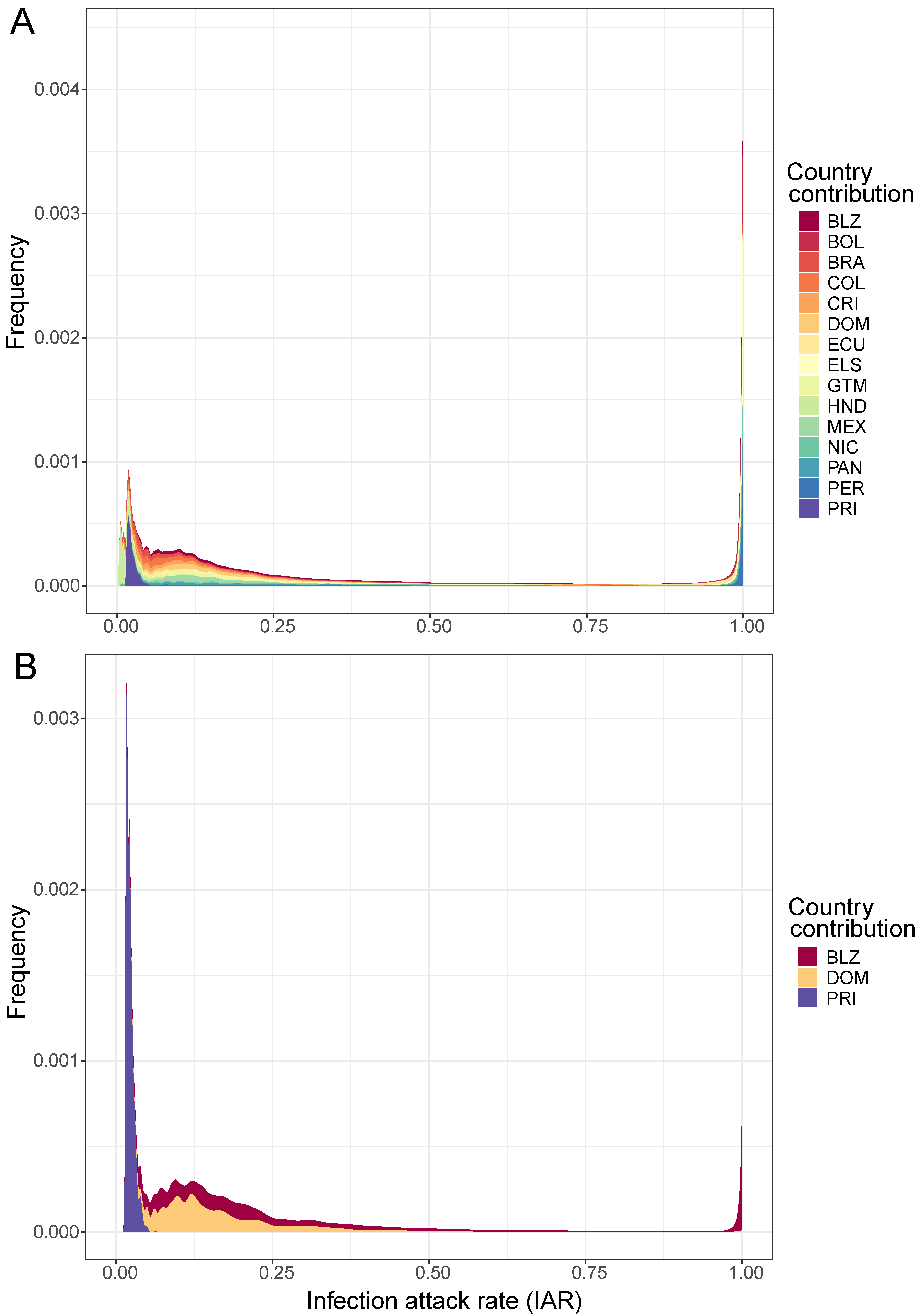

Supplement: S22 Fig — The different colors represent the probability distribution of IAR generated from using the estimated reporting probabilities from each modeled territory. (TIF) [file pntd.0008640.s033.tif]

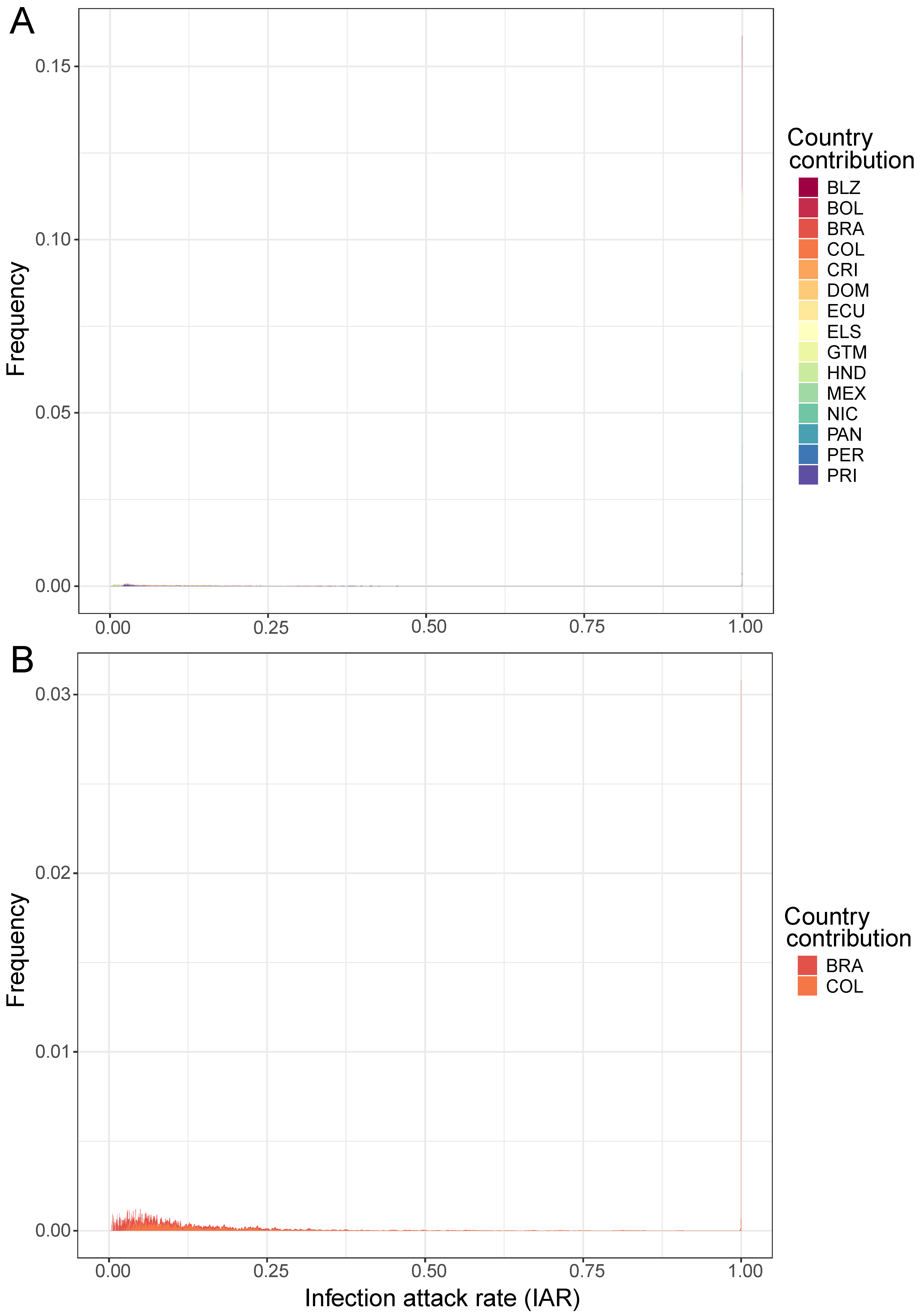

Supplement: S23 Fig — The different colors represent the probability distribution of IAR generated from using the estimated reporting probabilities from each modeled territory. (TIF) [file pntd.0008640.s034.tif]

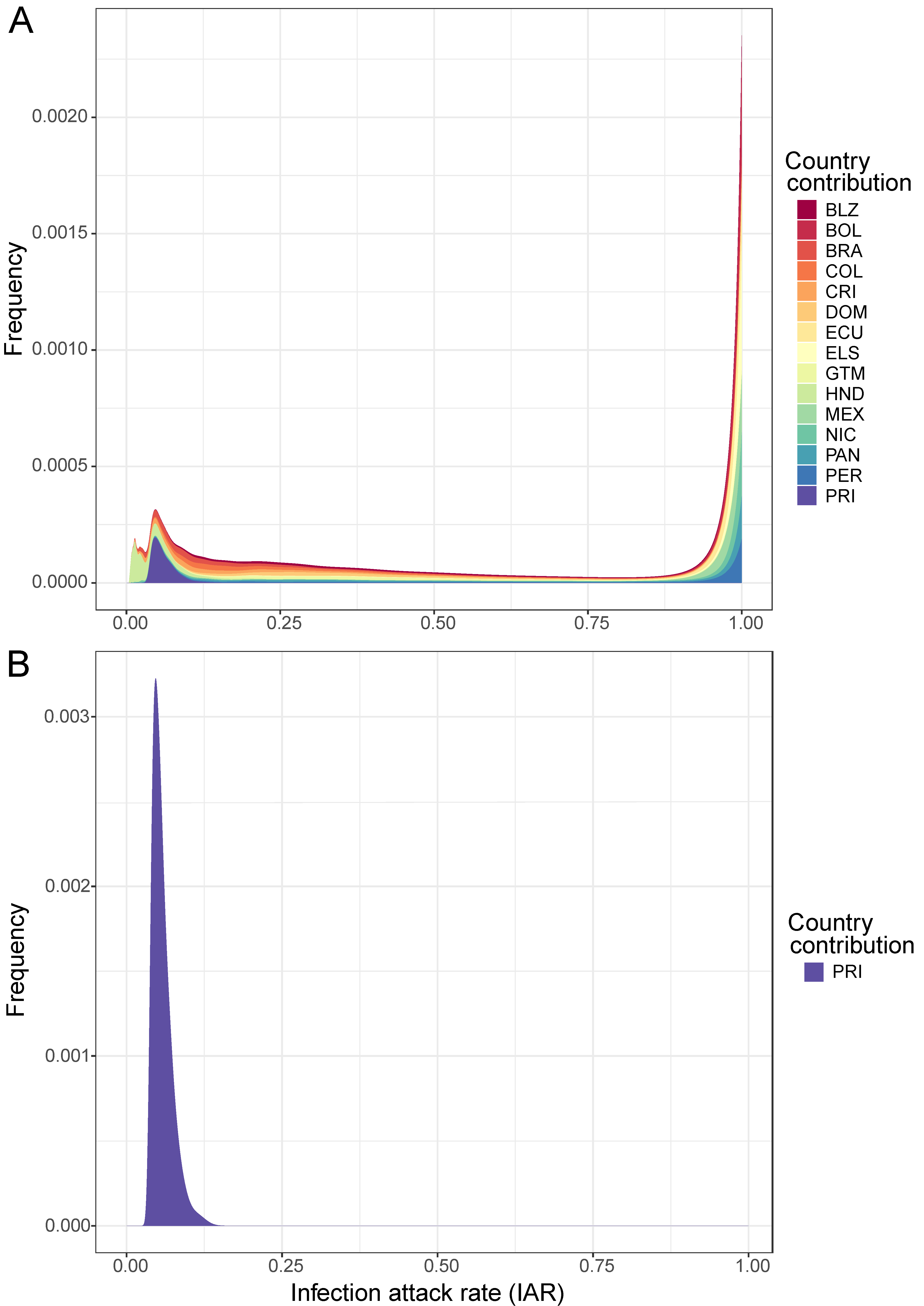

Supplement: S24 Fig — The different colors represent the probability distribution of IAR generated from using the estimated reporting probabilities from each modeled territory. (TIF) [file pntd.0008640.s035.tif]

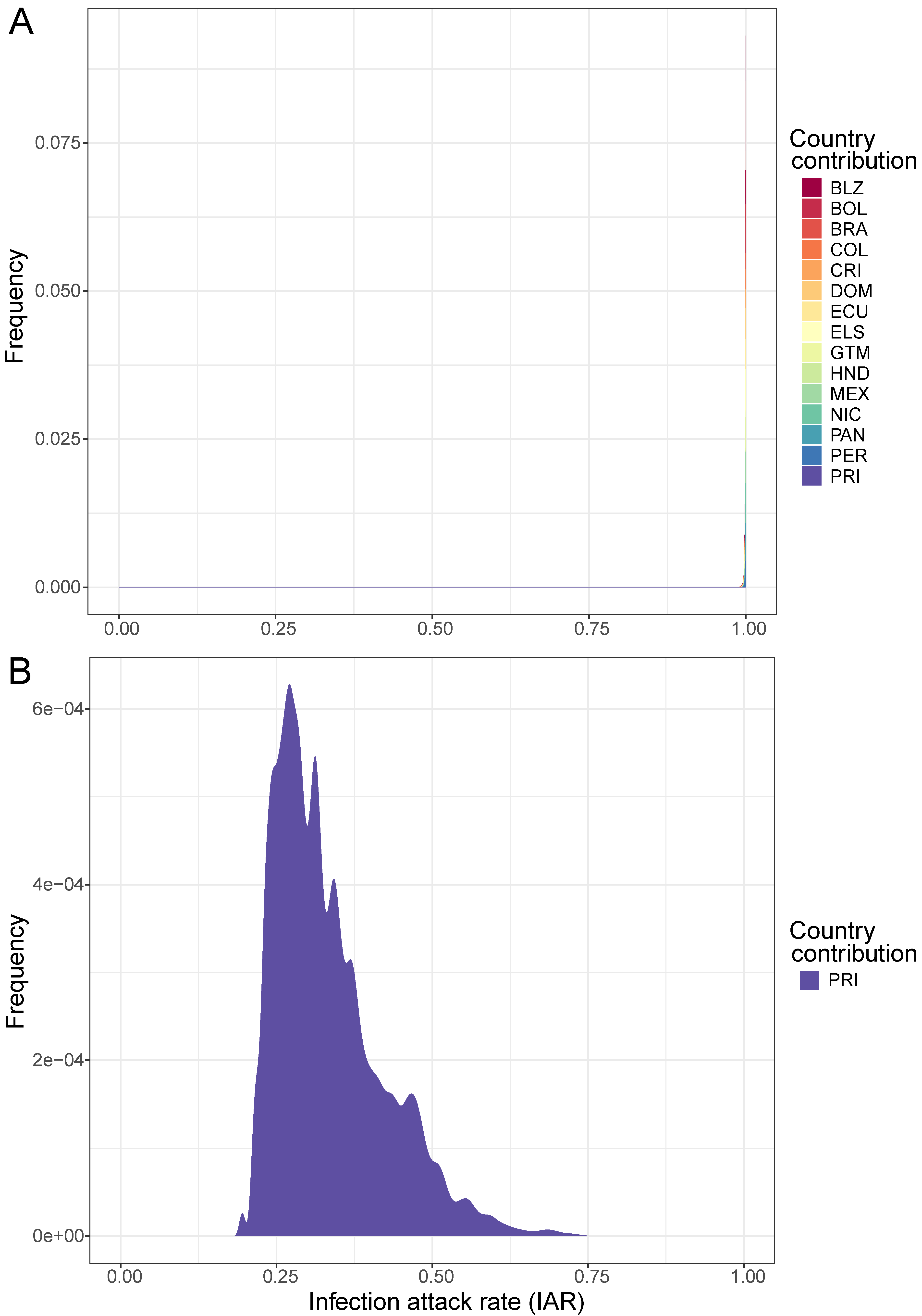

Supplement: S25 Fig — The different colors represent the probability distribution of IAR generated from using the estimated reporting probabilities from each modeled territory. (TIF) [file pntd.0008640.s036.tif]

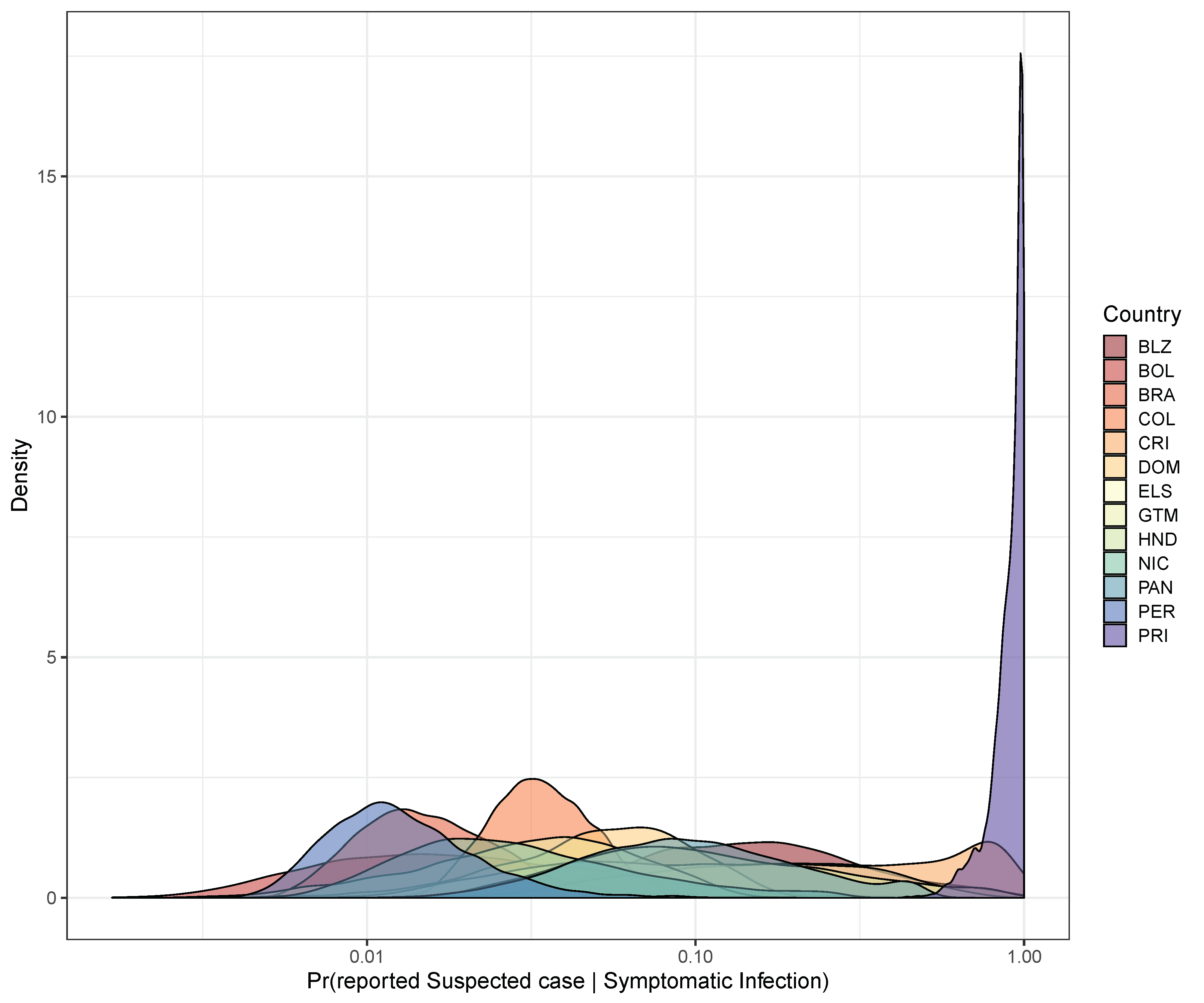

Supplement: S26 Fig — (TIF) [file pntd.0008640.s037.tif]

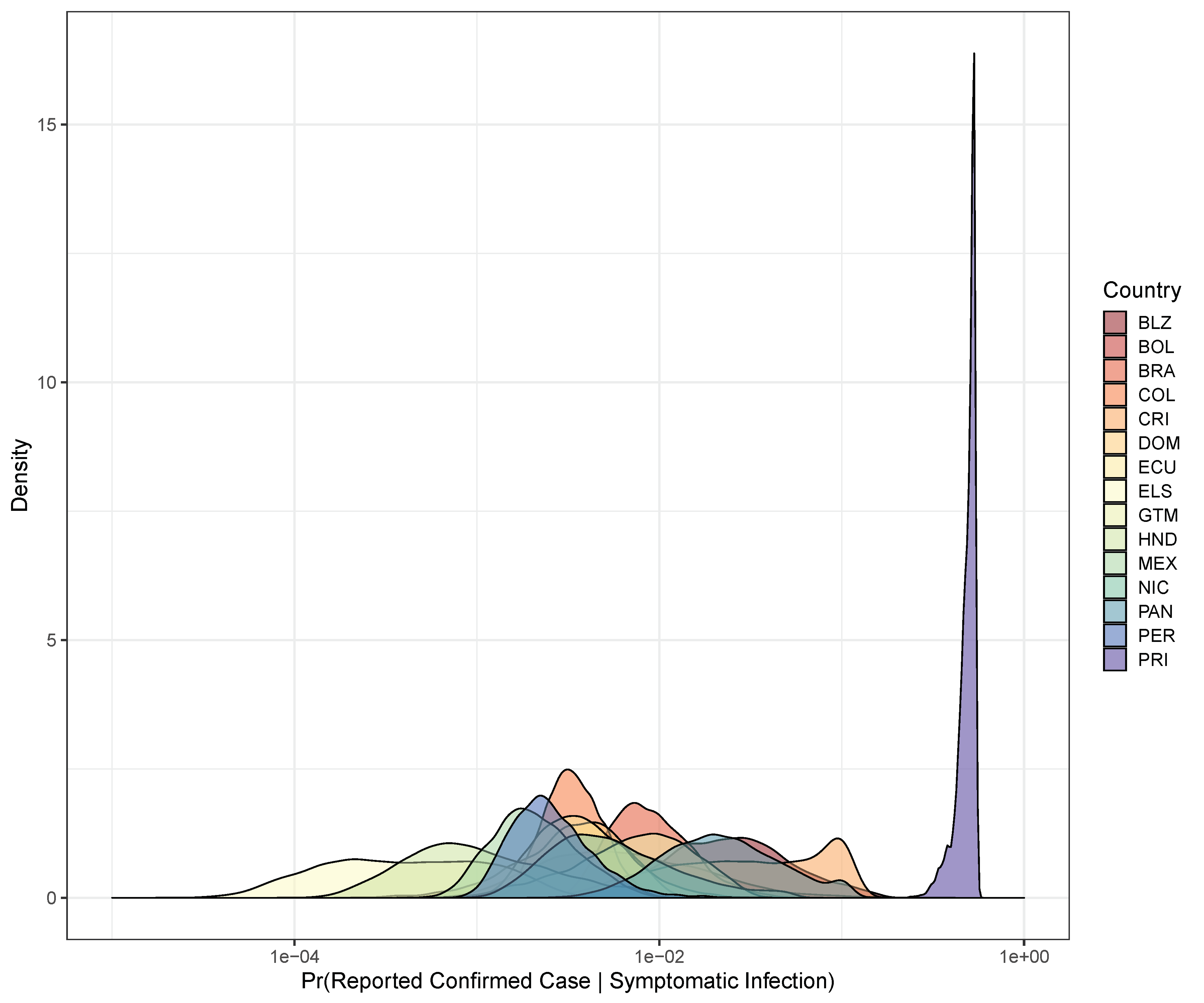

Supplement: S27 Fig — (TIF) [file pntd.0008640.s038.tif]

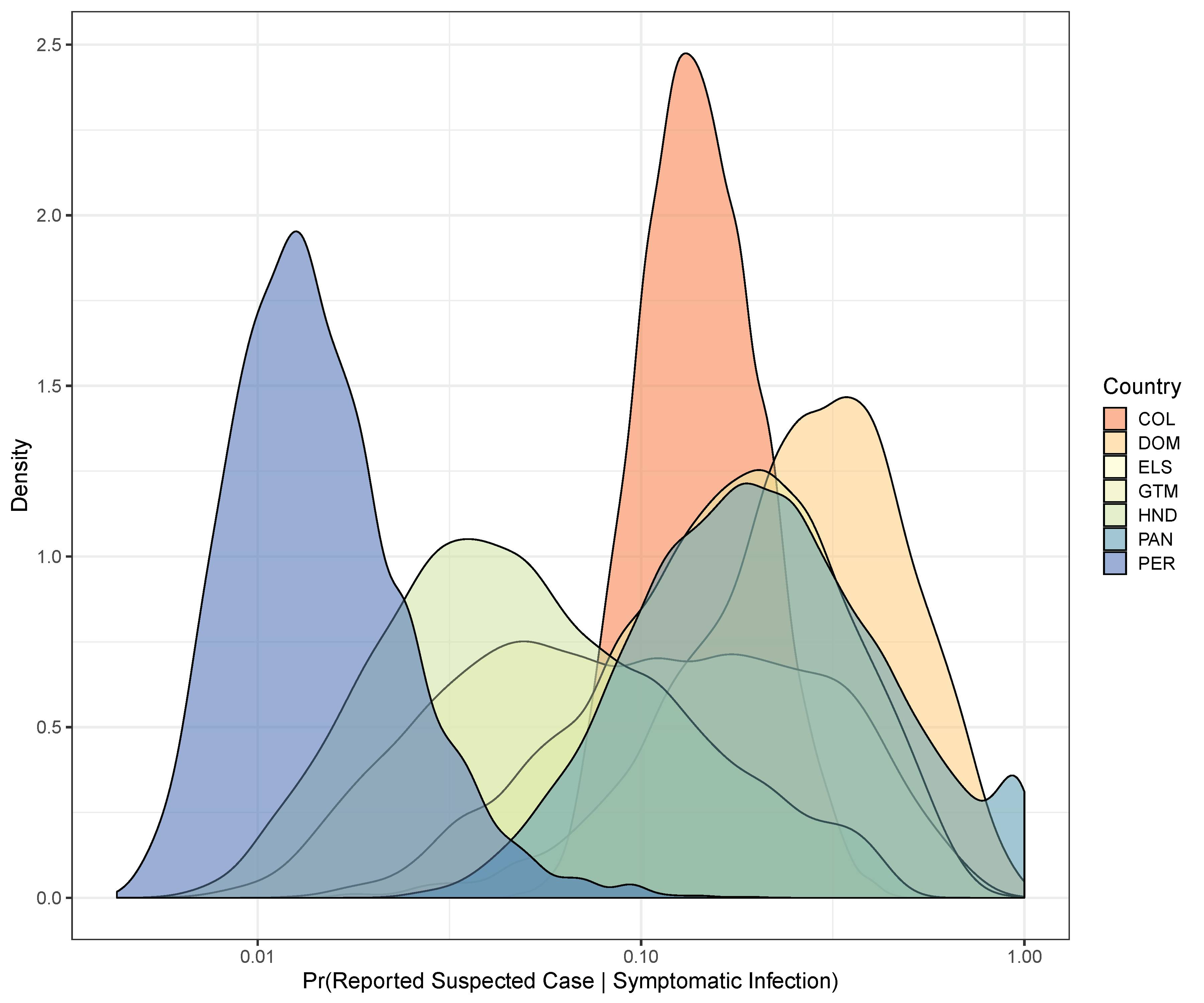

Supplement: S28 Fig — (TIF) [file pntd.0008640.s039.tif]

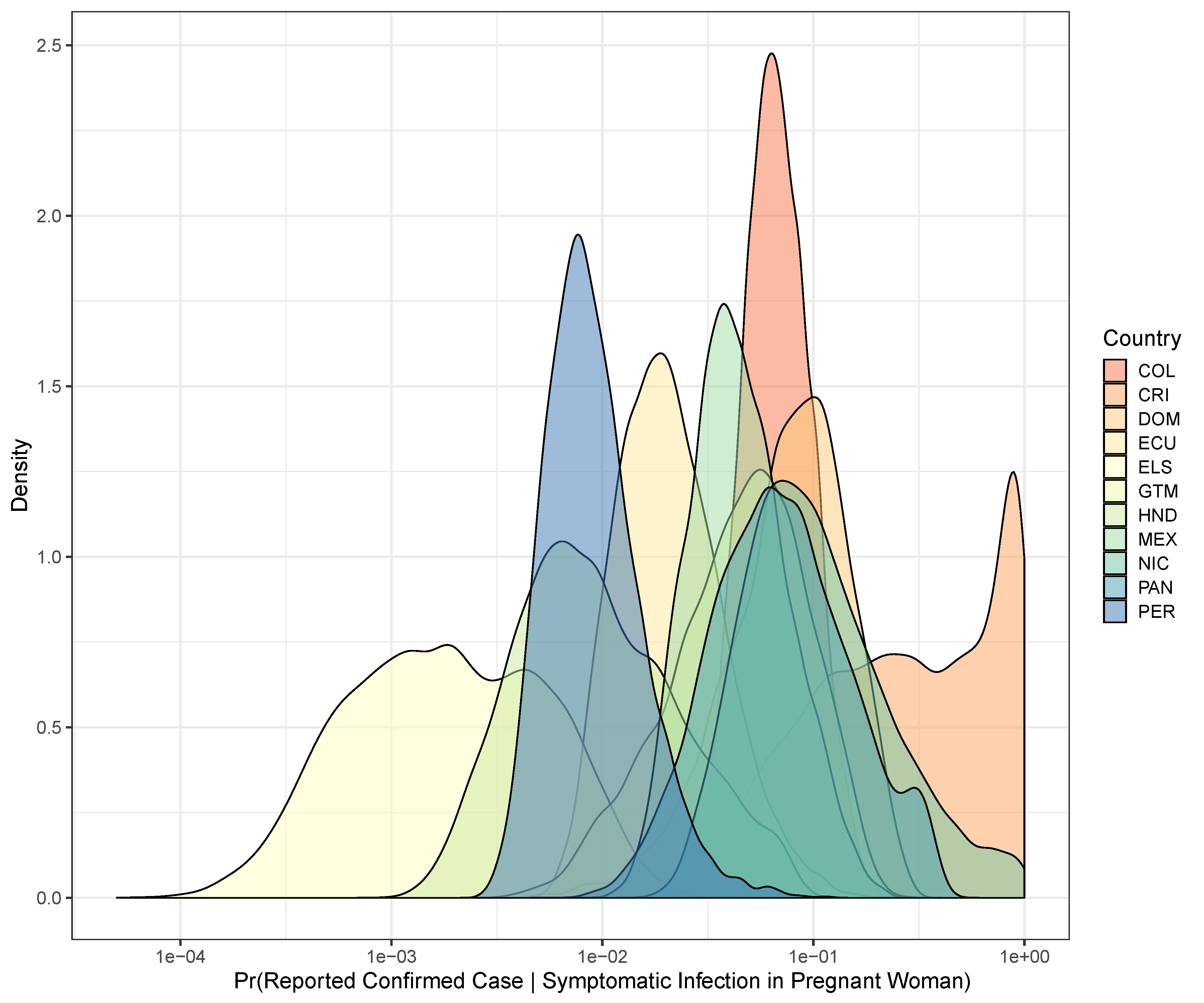

Supplement: S29 Fig — (TIF) [file pntd.0008640.s040.tif]

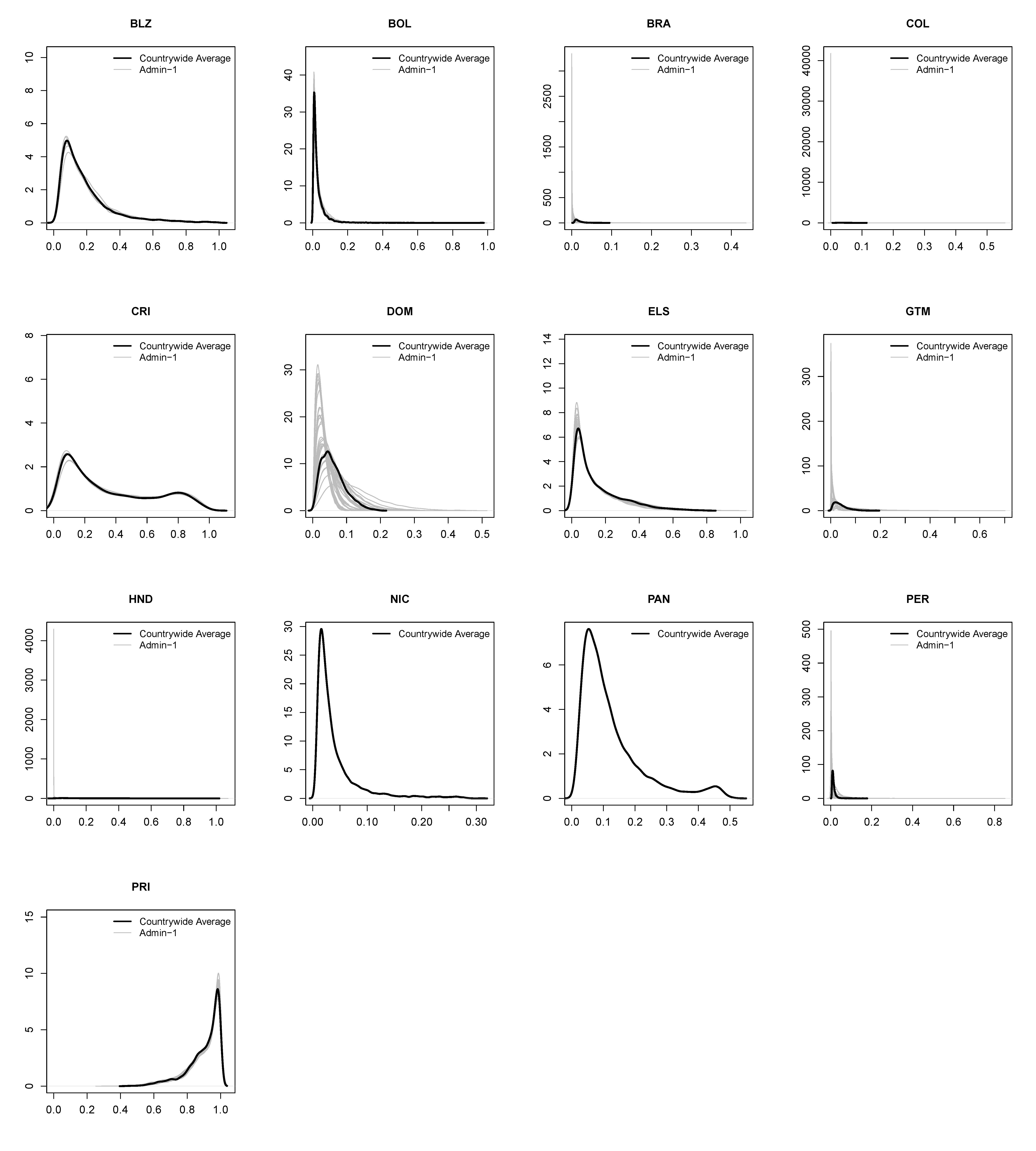

Supplement: S30 Fig — Black lines show the country-wide average reporting probability and grey lines show the estimated reporting probability in each administrative unit. (TIF) [file pntd.0008640.s041.tif]

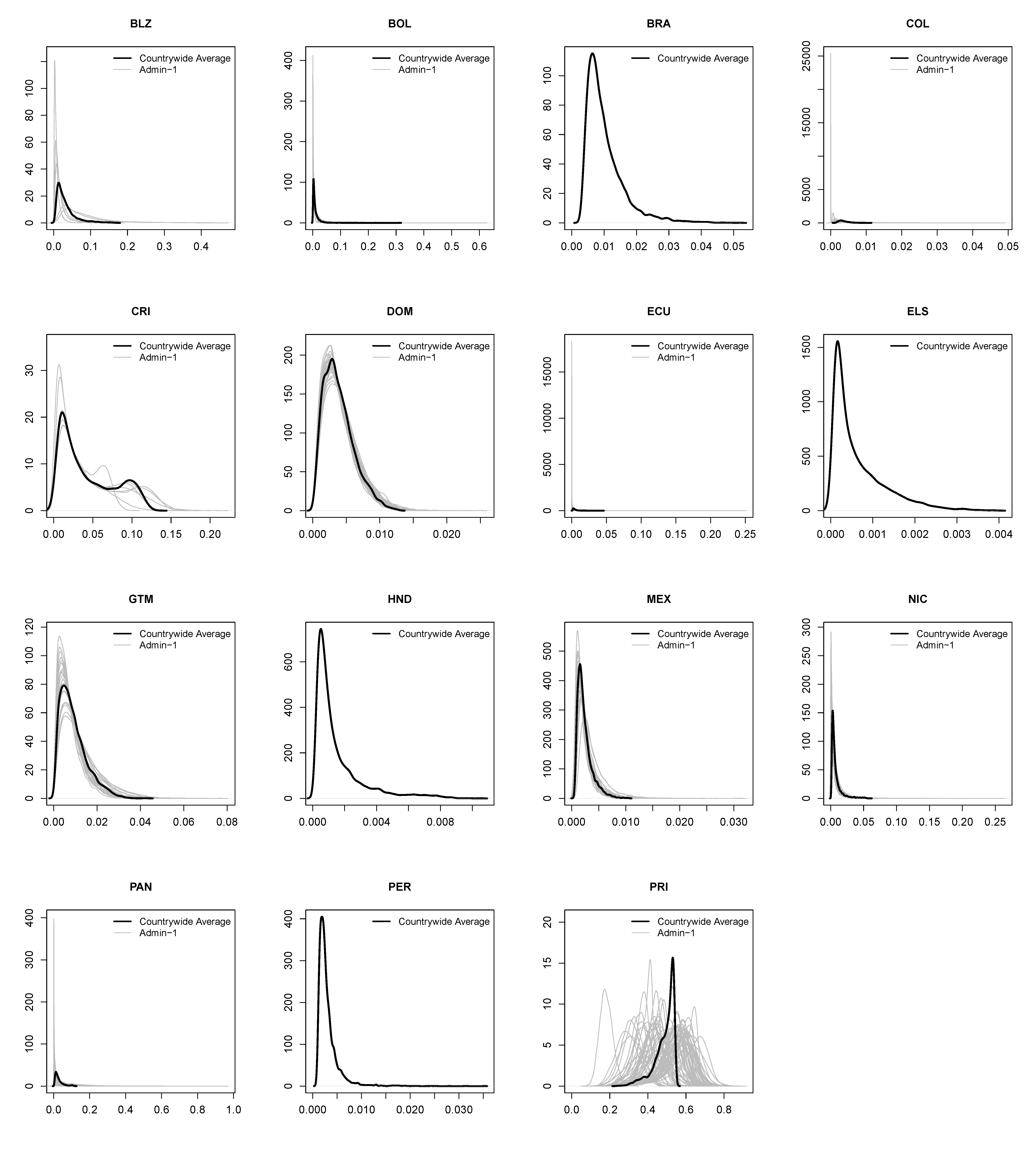

Supplement: S31 Fig — Black lines show the country-wide average reporting probability and grey lines show the estimated reporting probability in each administrative unit. (TIF) [file pntd.0008640.s042.tif]

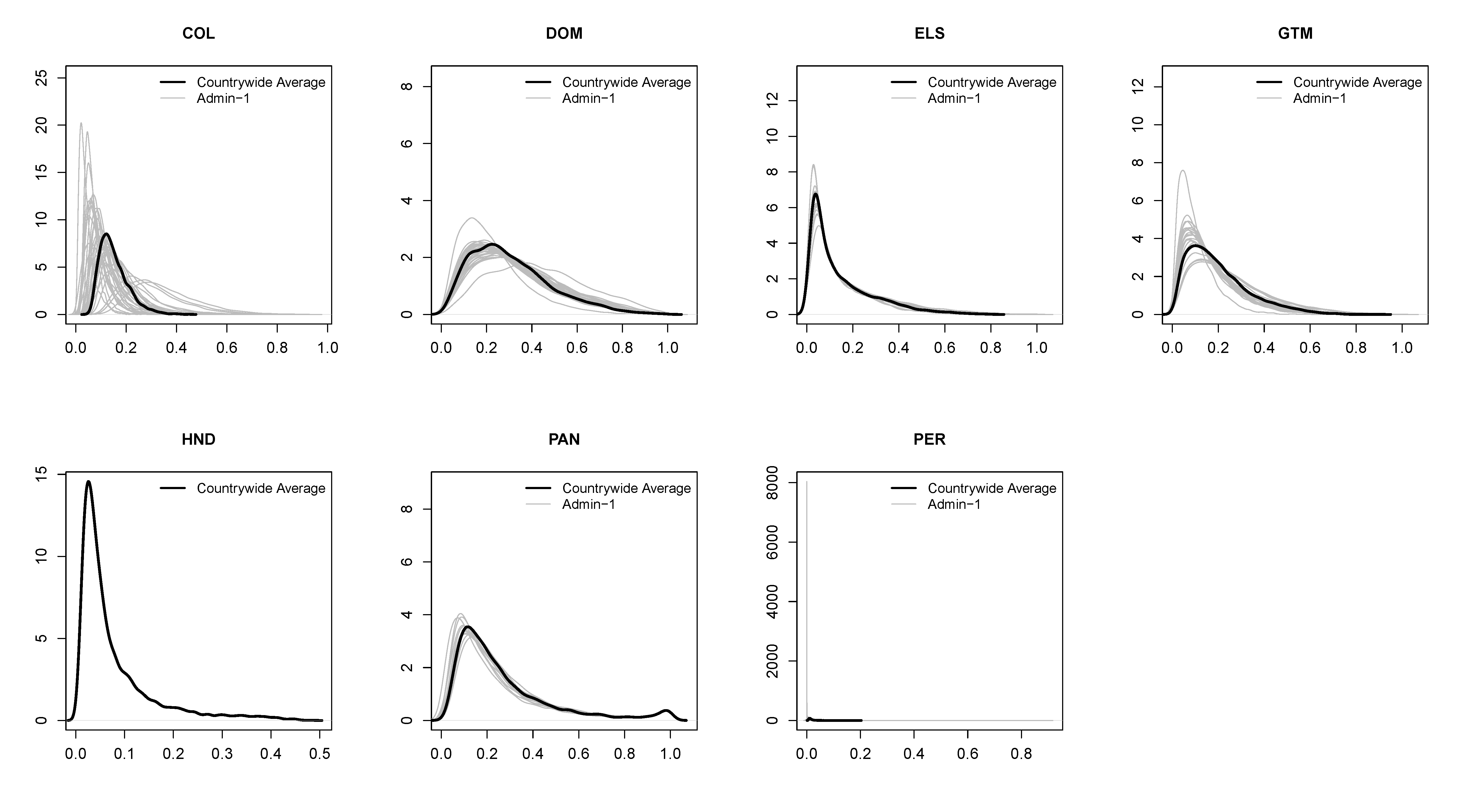

Supplement: S32 Fig — Black lines show the country-wide average reporting probability and grey lines show the estimated reporting probability in each administrative unit. (TIF) [file pntd.0008640.s043.tif]

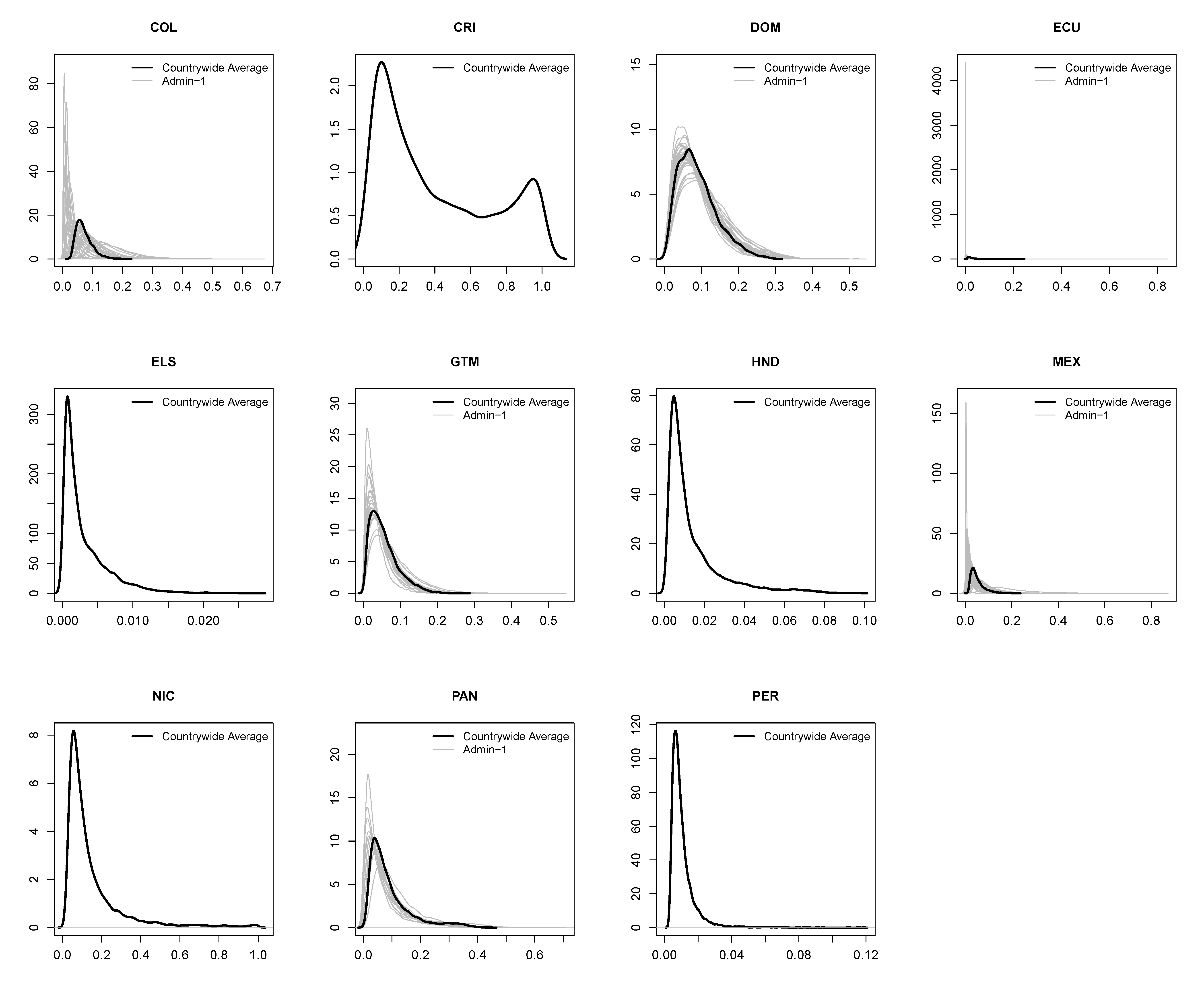

Supplement: S33 Fig — Black lines show the country-wide average reporting probability and grey lines show the estimated reporting probability in each administrative unit. (TIF) [file pntd.0008640.s044.tif]

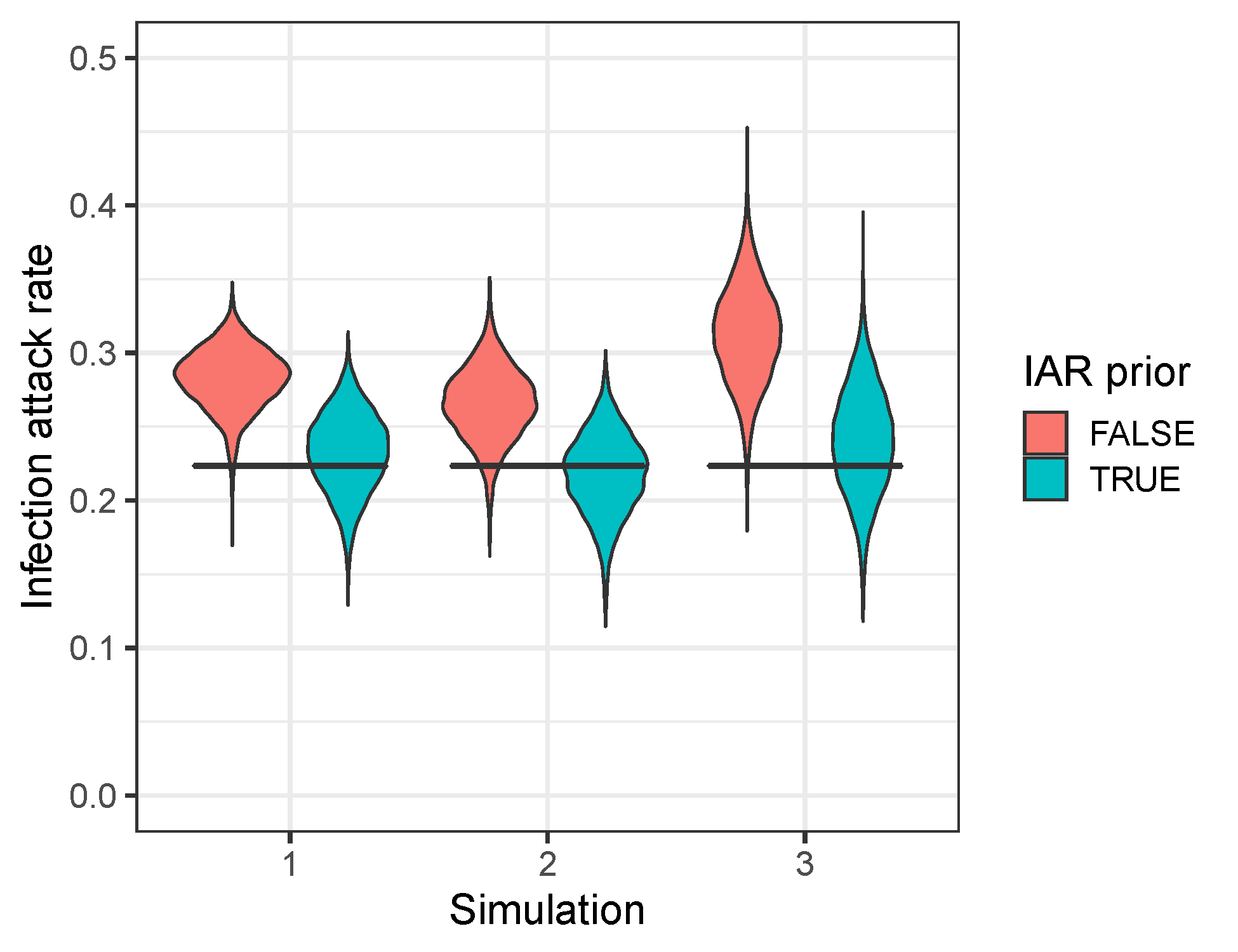

Supplement: S38 Fig — Simulation numbers are the three different simulated datasets with different symptomatic probability values. Blue values are posterior distributions from model using a Beta(1, 2) prior, and red values are posterior distributions from model with a flat prior. The solid lines are the simulated IAR value being estimated. (TIF) [file pntd.0008640.s049.tif]

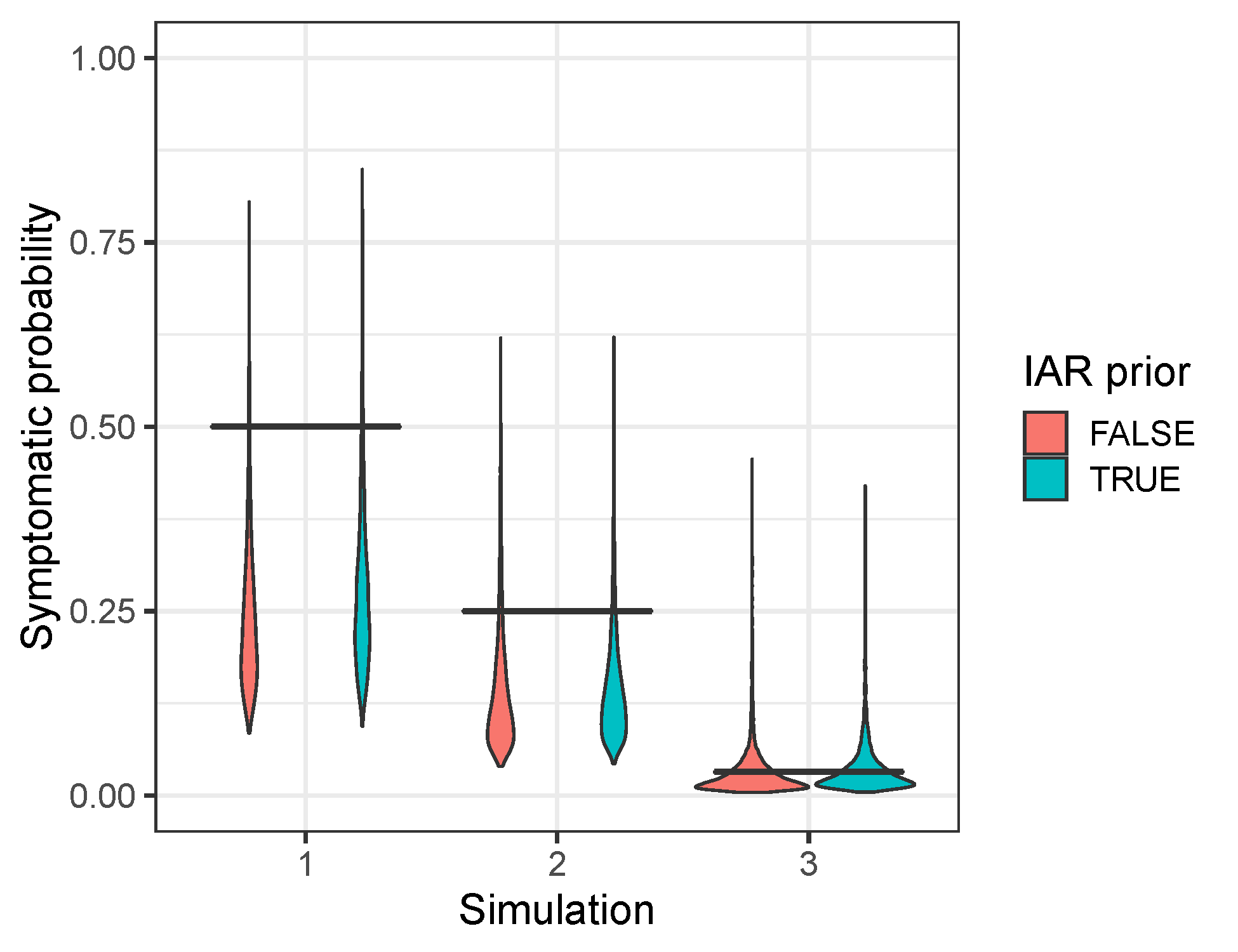

Supplement: S39 Fig — Simulation numbers are the three different simulated datasets with different ρZ values. Blue values are posterior distributions from model using a Beta(1, 2) prior, and red values are posterior distributions from model with a flat prior. The solid lines are the simulated ρZ value being estimated. (TIF) [file pntd.0008640.s050.tif]

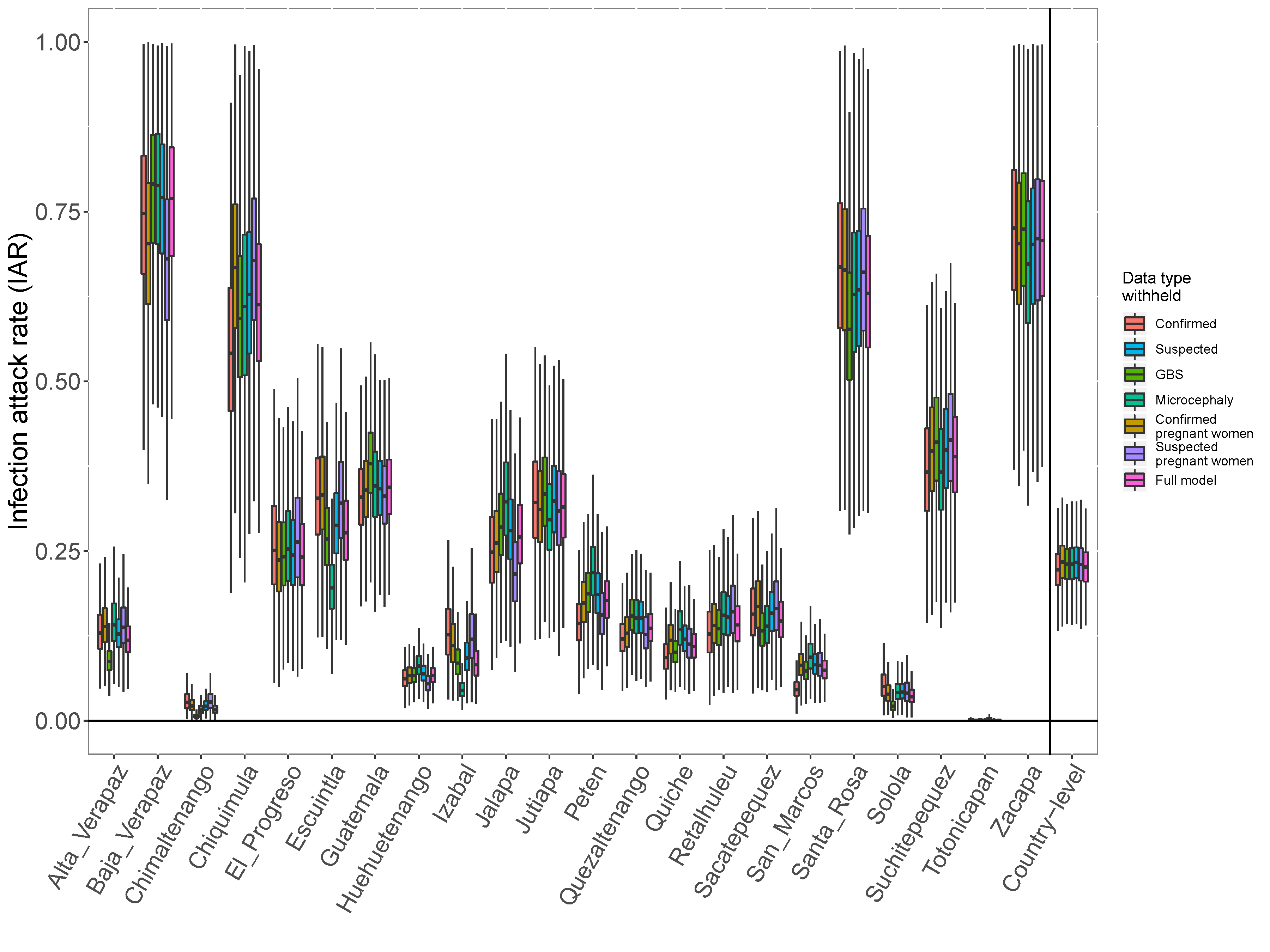

Supplement: S40 Fig — (TIF) [file pntd.0008640.s051.tif]

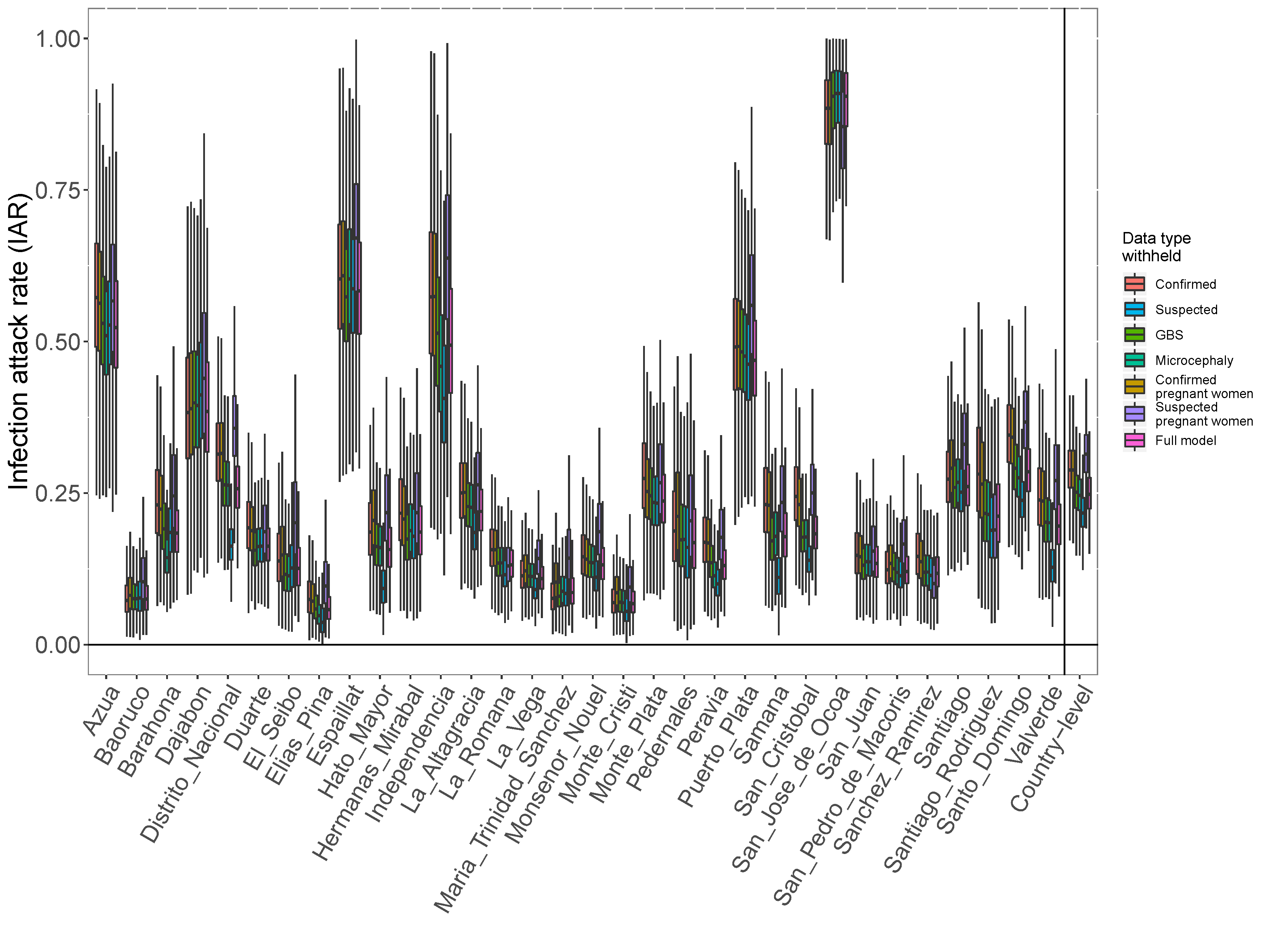

Supplement: S41 Fig — (TIF) [file pntd.0008640.s052.tif]

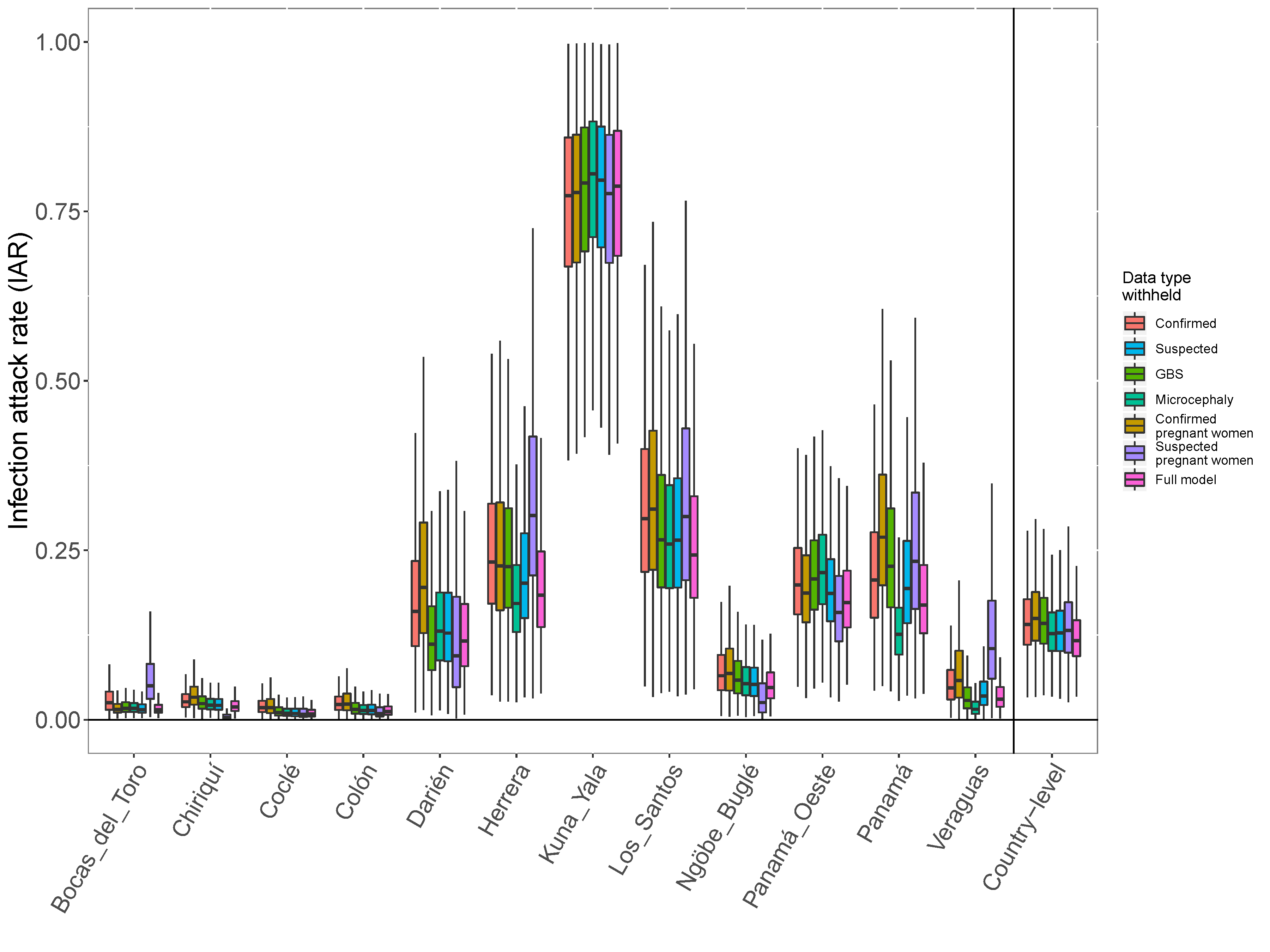

Supplement: S42 Fig — (TIF) [file pntd.0008640.s053.tif]

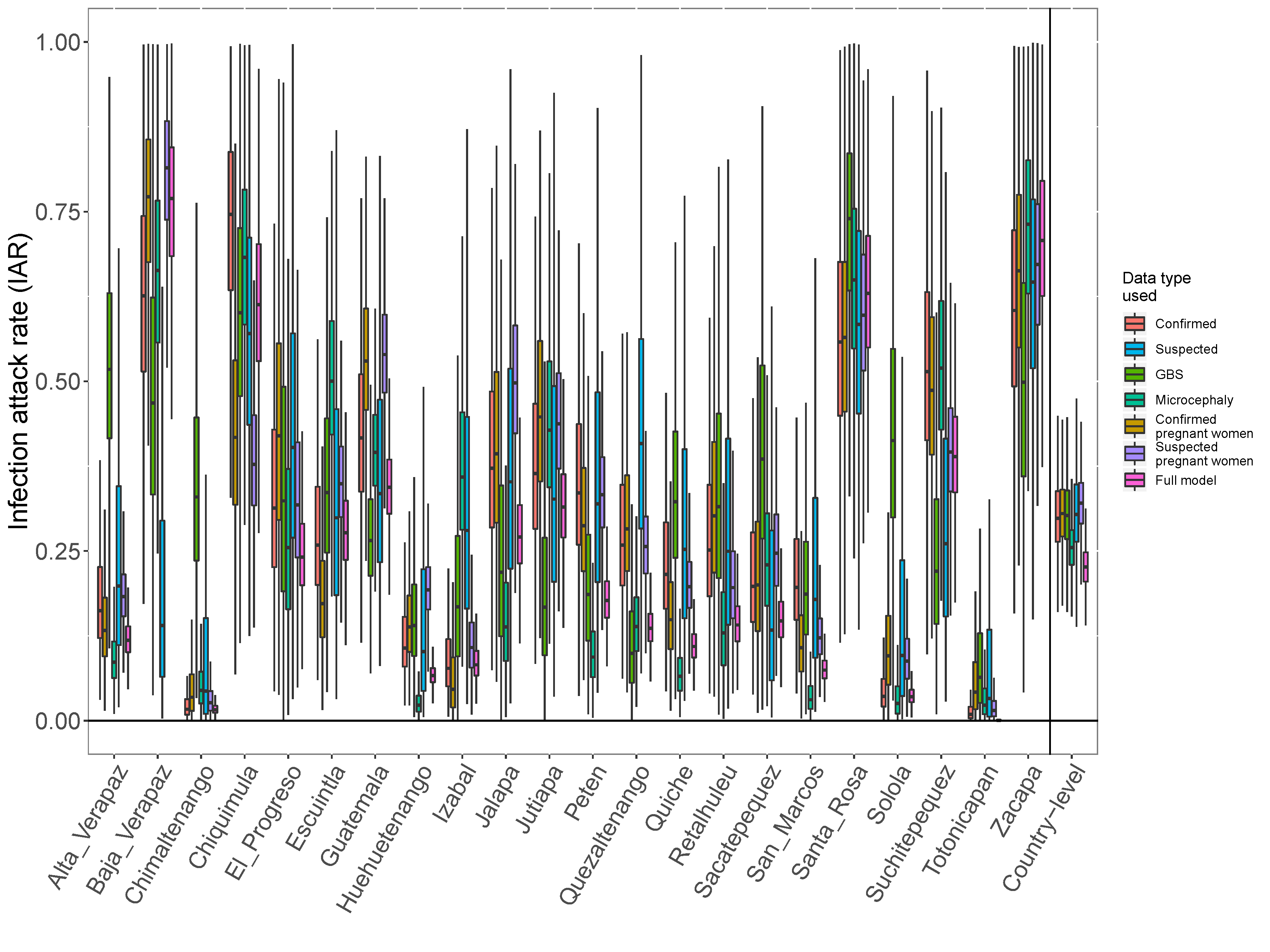

Supplement: S43 Fig — (TIF) [file pntd.0008640.s054.tif]

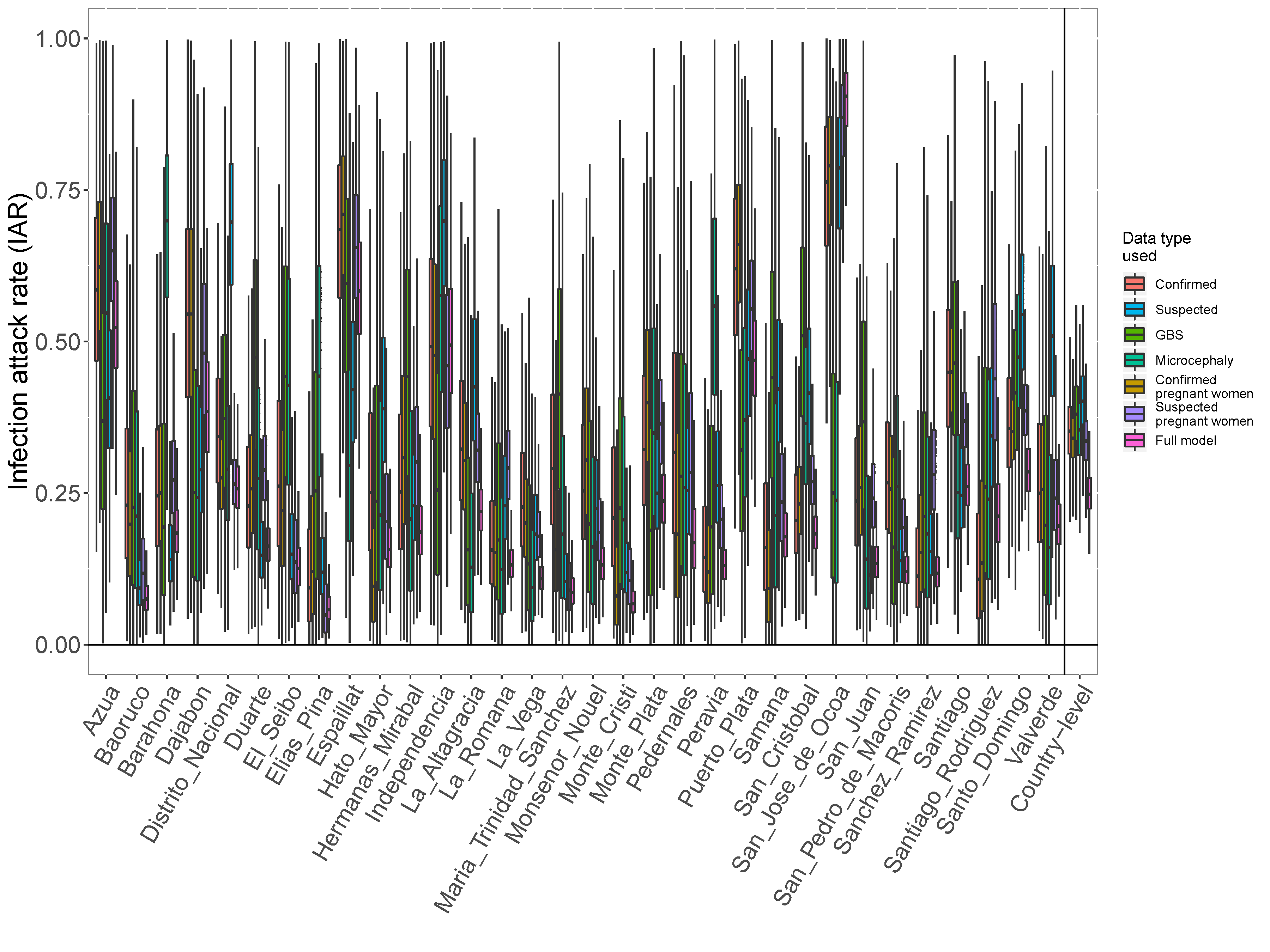

Supplement: S44 Fig — (TIF) [file pntd.0008640.s055.tif]

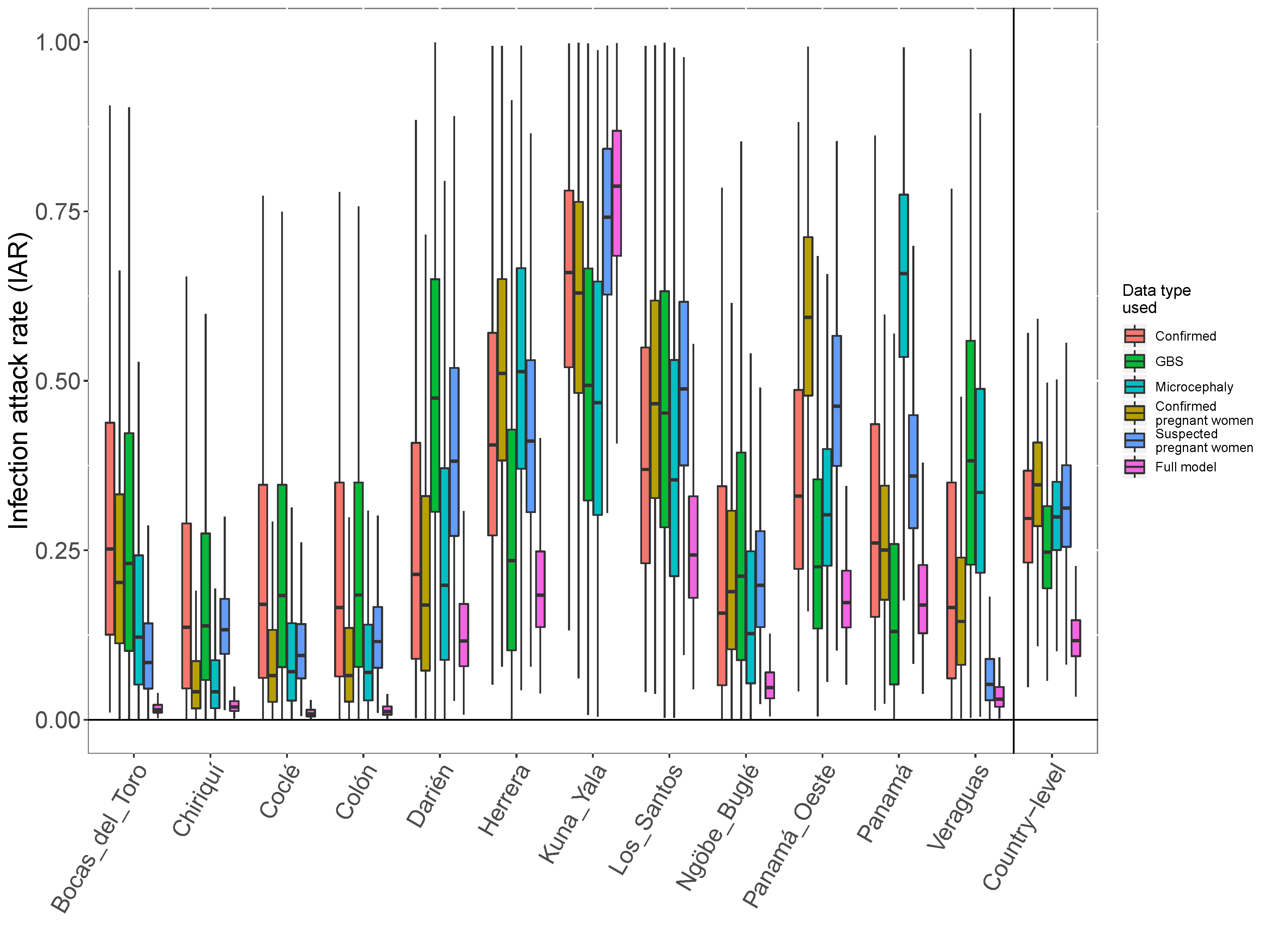

Supplement: S45 Fig — (TIF) [file pntd.0008640.s056.tif]
